# Assessing the Effectiveness of Bisphosphonates for the Prevention of Fragility Fractures: An Updated Systematic Review and Network Meta‐Analyses

**DOI:** 10.1002/jbm4.10620

**Published:** 2022-03-25

**Authors:** Anastasios Bastounis, Tessa Langley, Sarah Davis, Zoe Paskins, Neil Gittoes, Jo Leonardi‐Bee, Opinder Sahota

**Affiliations:** ^1^ Division of Epidemiology & Public Health, School of Medicine University of Nottingham, City Hospital Nottingham UK; ^2^ School of Health and Related Research, Regent Court (ScHARR) University of Sheffield Sheffield UK; ^3^ School of Medicine Keele University Keele UK; ^4^ Haywood Academic Rheumatology Centre Midlands Partnership NHS Foundation Trust Stoke‐on‐Trent UK; ^5^ Centre for Endocrinology, Diabetes and Metabolism (CEDAM) University of Birmingham Birmingham UK; ^6^ Queen Elizabeth Hospital University Hospitals Birmingham NHS Foundation Trust Birmingham UK; ^7^ Queens Medical Centre (QMC), University of Nottingham Nottingham University Hospitals NHS Trust Nottingham UK

**Keywords:** ANTIRESORPTIVES, BISPHOSPHONATES, FRACTURE PREVENTION, FRACTURES, INJURY/FRACTURE HEALING, NETWORK META‐ANALYSIS, OSTEOPOROSIS, SCREENING, SYSTEMATIC REVIEW

## Abstract

Bisphosphonates have been found to be effective in preventing fragility fractures. However, their comparative effectiveness in populations at risk has yet to be defined. In light of recent clinical trials, we aimed to compare four bisphosphonates (alendronate, ibandronate, risedronate, and zoledronate) and to identify which are the most effective for the prevention of fragility fractures. This is an update of a systematic review previously published as part of a NICE HTA report. We conducted a systematic review and network meta‐analysis, updating the estimates regarding the comparative effectiveness of the aforementioned bisphosphonates. Studies identified from published and unpublished sources between 2014 and 2021 were added to the studies identified in the previous review. Screening, data extraction and risk of bias assessment were independently undertaken by two reviewers. Outcomes were fractures, femoral neck bone mineral density (BMD), mortality, and adverse events. We identified 25 additional trials, resulting in a total population of 47,007 participants. All treatments had beneficial effects on fractures versus placebo with zoledronate being the most effective treatment in preventing vertebral fractures (hazard ratio [HR] 0.38; 95% credibility interval [CrI], 0.28–0.49). Zoledronate (HR 0.71; 95% CrI, 0.61–0.81) and risedronate (HR 0.70; 95% CrI, 0.53–0.84) were found to be the most effective treatments in preventing nonvertebral fractures. All treatments were associated with increases in femoral neck BMD versus placebo with zoledronate being the most effective treatment mean difference (MD 4.02; 95% CrI, 3.2–4.84). There was a paucity of data regarding hip and wrist fractures. Depending on its cost‐effectiveness, zoledronate could be considered a first‐line option for people at increased risk of fragility fractures. © 2022 The Authors. *JBMR Plus* published by Wiley Periodicals LLC on behalf of American Society for Bone and Mineral Research.

## Introduction

Bisphosphonates, such as alendronate (ALN), risedronate (RIS), ibandronate (IBN), and zoledronate (ZOL), have been found to be effective in reducing the risk of osteoporotic fragility fractures.^(^ ^1^ ^)^ However, there is no conclusive evidence regarding their comparative effectiveness in specific patient groups, such as patients with low bone mineral density (BMD).^(^ ^2^ ^)^ This can be accounted for by the paucity of comparative trials that would provide insight on how bisphosphonates work through time in the light of adverse events associated with the use of bisphosphonates.^(^ ^2^ ^)^ There is a need, therefore, to undertake a comparative evaluation of bisphosphonates, testing their effectiveness in reducing the risk of fragility fractures.

This is an update of a systematic review that was previously published as part of a National Institute for Health and Care Excellence (NICE) health technology assessment (HTA) report.^(^ ^3^ ^)^ The update of the systematic review is timely given that there are recently published trials which are likely to alter the confidence in findings, providing an opportunity to update estimates to facilitate clinical decision‐making.^(^ ^4^ ^)^ In the current review, five interventions were considered: alendronate 10 mg/daily or 70 mg/weekly (ALN), orally‐administered ibandronate 150 mg/monthly (IBN‐oral), intravenously‐administered ibandronate 3 mg/quarterly (IBN‐iv), risedronate 5 mg/daily or 35 mg/weekly (RIS), and zoledronate 5 mg/annually (ZOL). Supplementary to fractures, this review also investigated the effects of bisphosphonates on femoral neck BMD, health‐related quality‐of‐life (HRQoL), adverse events, and mortality. Within the context of osteoporosis, BMD constitutes a biological surrogate measure of patients' risk to develop fragility fractures,^(^ ^5^ ^)^ although recent evidence has shown that treatment‐induced BMD changes at femoral neck predict lower risk in developing vertebral, nonvertebral, and hip fractures.^(^ ^6^ ^)^ The aim of this systematic review was to provide updated estimates regarding the comparative effectiveness of the aforementioned bisphosphonates, which in turn will inform an economic evaluation regarding bisphosphonates' benefit‐to‐risk ratio.

## Methods

This network meta‐analysis is an update of a systematic review that was previously published as part of a NICE HTA report.^(^ ^3^ ^)^ This study was reported following the Preferred Reporting Items for Systematic Reviews and Meta‐Analyses (PRISMA) Extension Statement for Reporting of Systematic Reviews Incorporating Network Meta‐Analyses of Health Care Interventions checklist (Appendix 12).^(^ ^7^ ^)^ This systematic review and network meta‐analysis has been registered with the PROSPERO database (CRD42020177155).^(^ ^8^ ^)^

### Eligibility criteria

The eligibility criteria of this systematic review have been described.^(^ ^3^ ^)^ Briefly, only studies in which the interventions of interest (ALN, IBN‐iv, IBN‐oral, RIS, and ZOL) have been assessed within their licensed doses for treating osteoporosis were eligible for inclusion. Studies that report data for both licensed and unlicensed dose study groups were considered eligible only if data for the licensed groups were separately reported. Studies reporting comparisons among the interventions of interest were considered eligible for inclusion. Interventions could also be compared with placebo or other nonactive treatments (eg, treatment without the potential to augment bone, calcium/vitamin D). Outcomes consisted of fragility fractures, BMD at femoral neck, mortality, adverse effects, and HRQoL. Only randomized controlled trials (RCTs) were eligible for inclusion.

### Search strategy and information sources

A comprehensive search was undertaken to systematically identify eligible studies regarding the aforementioned bisphosphonates' effects in preventing the occurrence of fragility fractures (Appendix 1). Only studies published in the English language were included at the full‐text stage, given that no relevant studies published in other languages were identified. The search strategy comprised the following main elements: searching of electronic databases (including unpublished data and trial registries), extensive keyword hand‐searching, and scrutiny of bibliographies of retrieved papers. The following databases were searched:

•MEDLINE® In‐Process & Other Non‐Indexed Citations and MEDLINE® (Ovid), including PubMed;

•EMBASE (Ovid);

•Cochrane Database of Systematic Reviews (Wiley Interscience);

•Cochrane Central Register of Controlled Trials (CENTRAL) (Wiley Interscience);

•Cumulative Index to Nursing and Allied Health Literature (CINAHL, EBSCO);

•Database of Abstract of Reviews of Effects (Wiley Online Library);

•Health Technology Assessment Database (CRD Database);

•NHS Economic Evaluation Database (CRD Database);

•OpenGrey;

•Science Citation Index (ISI Web of Knowledge);

•Conference Proceedings Citation Index ‐ Science (Web of Science);

•ClinicalTrials.gov.

Searches of Medline, EMBASE, CINAHL, and CENTRAL covered the period from September 2014, to March 1, 2021. Searches of the rest of databases and trial registries were conducted from 2014 to February 8, 2021. All potentially relevant citations were downloaded to Endnote X8 Reference Manager bibliographic software (version 8.0; Clarivate Analytics, Philadelphia, PA, USA).

### Study selection, data collection process, and data items

Newly‐identified studies were imported into Rayyan online software.^(^ ^9^ ^)^ Two independent reviewers screened studies for relevance based on titles/abstracts and later full‐texts (AB, TL) with disagreements resolved through discussion or by consulting a third reviewer (OS). Two independent reviewers (AB, TL) conducted full‐text screening with a high‐level of agreement (κ = 0.91). A standardized and pilot‐tested data extraction form was used to extract relevant data. One reviewer (AB) extracted data with a second reviewer (TL) independently checking at least of 80% of the extracted records. Where multiple publications of the same study were identified, data extraction was undertaken on the associated publications where relevant data exists. Where different follow‐ups of an eligible study were identified, these were included in the extraction phase where relevant data existed. Data extracted consisted of the following categories: (i) descriptive statistics (eg, number recruited and randomized, participants' characteristics); (ii) baseline data on outcomes of interest (eg, comorbidities, fractures at baseline, alcohol use, number of falls); (iii) moderators of action (eg, glucocorticoids [GC] use, patients with osteoporosis, history of fractures/fractures at baseline); (iv) intervention characteristics (eg, drug‐type, administration mode, concomitant treatments); (v) statistics and relevant data on the main outcome expressed either as continuous or binary outcomes; (vi) data on adverse events (total and by type); and (vii) data on mortality and HRQoL. Authors were contacted when there was lack of data on outcomes of interest and/or further information were needed in order to attest eligibility of relevant studies.

### Geometry of networks

Both treatment‐placebo and treatment‐active comparisons were examined and network plots were created for all outcomes (Appendix 3). Nodes indicate the different treatments included in the analysis and thickness of edges connecting the nodes indicate the number of studies informing each comparison (thicker lines indicate more populated comparisons). For those from the main outcomes with connected networks (ie, femoral neck BMD and vertebral fractures), an additional visual representation of network plots is provided (Appendix 7). Node size indicates the number of studies included in each node and thickness of lines indicate the overall sample size informing each comparison (thicker edges indicate more populated pairwise comparisons).

### Risk of bias within individual studies

The methodological quality of the included RCTs was independently assessed at the study‐level by two reviewers (AB, JLB), using the Cochrane Collaboration risk of bias tool 1.0.^(^ ^10^ ^)^ Any disagreements were resolved through discussion. The Cochrane Collaboration risk of bias tool 1.0 addresses the following specific domains: sequence generation; allocation concealment; blinding of participants and personnel; blinding of outcome assessment; and incomplete outcome data and selective outcome reporting. Studies were rated with a low‐risk of bias in randomization sequence if they provided an explicit statement on how they performed the randomization. Open‐label trials were rated as high risk in the “blinding” category, whereas higher than 20% attrition at 12 months' follow‐up resulted in high‐risk rating in the “incomplete outcome data” category. Risk‐of‐bias plots were created by using the “robvis” tool.^(^ ^11^ ^)^

### Summary measures and methods of analysis

Fractures, mortality, and adverse events were reported in a binary form (number of participants experiencing at least one event out of the total number of participants). The data generation process followed a binomial likelihood, assuming an underlying Poisson process for each trial arm. The complementary log–log link function was used to model the network meta‐analyses (NMAs) for the binary outcomes.^(^ ^12^ ^)^ Log hazard ratios (HRs) were estimated from the median and corresponding 95% credibility intervals (CrIs) from the 2.5th and 97.5th centiles of the posterior distribution. Treatment ranking probabilities for all fracture outcomes are reported. Changes in BMD were reported as percentage changes per arm from baseline (mean percentage difference per arm plus standard error of the mean [SE]). The data generation process followed a normal likelihood. The identity link function was used to model the NMA for BMD change, including study duration as a trial‐level covariate and assuming an equal interaction effect between treatments and reference treatment one.^(^ ^13^ ^)^ The treatment effects represent the mean difference between the percentage change in the treatment group and the comparator group. Mean percentage difference plus 95% CrI were estimated from the posterior distribution. Treatment ranking probabilities and surface under the cumulative ranking (SUCRA) are reported for the BMD data.^(^ ^14^ ^)^

Two different modeling strategies were considered for the treatment effects: (i) a standard, independent random (treatment)‐effects model^(^ ^15^ ^)^ was fitted for assessing the comparative effectiveness of bisphosphonates in increasing femoral neck BMD; and (ii) exchangeable treatment‐effects models (ie, effects model where the treatment effects are assumed to arise from a common distribution according to the class of drug)^(^ ^16^, ^17^ ^)^ were fitted for assessing the comparative effectiveness of bisphosphonates in preventing fractures, deaths, and adverse events, given the relative paucity of data in the aforementioned variables. For BMD changes, the model was completed by using conventional reference prior distributions: (i) trial‐specific baseline, μ_i_ ∼ N(0,100^2^); (ii) treatment effects relative to reference treatment, d_1k_ ∼ N(0,100^2^); and (iii) between‐study standard deviation (SD) of treatment effects, τ ∼ U(0,100). Where there were sufficient data for binary outcomes, conventional reference prior distributions were used: (i) trial‐specific baseline, μ_i_ ∼ N(0, 100^2^); (ii) treatment effects relative to reference treatment, d_1k_ ∼ N(0, 100^2^); and (iii) between‐study SD of treatment effects, τ ∼ U(0, 5). Due to the paucity of data, we used a weakly informative prior distribution for the between‐study SD (ie, τ ∼ HN(0,0.32^2^)) for the NMAs of hip and wrist fractures, and specific‐type adverse events (ie, influenza‐like symptoms, myalgia, nasopharyngitis, and headache). Based on clinical plausibility, a weakly informative prior distribution for the between‐study SD (ie, τ ∼ HN(0,0.32^2^)) was used for the NMA of mortality data.

All analyses were conducted using OpenBUGS (MRC Biostatistics Unit, Cambridge, UK)^(^ ^18^ ^)^ and R Studio (R version 4.0.3),^(^ ^19^ ^)^ using the “gemtc”^(^ ^20^, ^21^ ^)^ and “rjags”^(^ ^22^ ^)^ packages. Convergence to the target posterior distributions was assessed using the Gelman–Rubin statistic for three independent chains with different initial values. For all outcomes, results were based on three independent chains of initial values and 105,000 iterations after a burn‐in of 50,000 iterations. Most of NMAs exhibited moderate correlation between successive iterations of the Markov chain, so were thinned by retaining every 10th sample.

### Assessment of inconsistency

Consistency of evidence was assessed by using the node‐splitting method^(^ ^23^, ^24^, ^25^ ^)^ in OpenBUGS and RStudio (R version 4.0.3). Differences between direct and indirect evidence in all network loops were calculated with *p* values <0.05 indicating the presence of significant inconsistency. In the case of fracture data, inconsistency was assessed for vertebral fractures only. For nonvertebral fractures, no indirect evidence was available. For hip fractures, an assessment of inconsistency was not performed because the direct evidence between ALN and RIS was provided by one small and, unbalanced in terms of sample size, study^(^ ^26^ ^)^ with zero events in one arm. For wrist fractures, an assessment of inconsistency was not performed because the direct evidence between ALN and RIS was provided by the same small study and the only direct evidence between ALN and oral IBN‐oral was provided by the only three‐arm study included in the NMA.^(^ ^27^ ^)^ For BMD data, the assessment of inconsistency was performed after excluding an outlier study,^(^ ^28^ ^)^ which was the only study informing the direct relationship between ZOL and ALN, and the three‐arm study,^(^ ^27^ ^)^ which was the only study providing direct evidence for the relationship between RIS and IBN‐oral. For the overall adverse events outcome, an assessment of inconsistency was not formally performed because the fit of the model with the data was poor. For myalgia, headache, and pyrexia, assessment of inconsistency was not performed because there was no indirect evidence. For influenza‐like symptoms, an assessment of inconsistency was not performed because there was only one small study with zero events in the control arm informing the direct relationship between IBN‐oral and placebo and three small studies with zero events in control arms informing the direct relationship between ZOL and placebo.

### Credibility of the findings/risk of bias across studies

A post hoc assessment of methodological quality of the included studies was undertaken at outcome level. A more liberal assessment was applied to the categories of “blinding” and “incomplete outcome data,” taking into account that the NMAs assessed pharmacological treatment effects on objective outcomes. When attrition was comparable between arms (≤10%) at follow‐up, a low risk rating was applied. Our aim was to appropriately evaluate the credibility of results obtained from the NMA of RCTs with different endpoints. The assessment of the credibility of findings was conducted by following the Confidence in Network Meta‐Analysis (CINeMA) approach,^(^ ^29^ ^)^ where the credibility of findings is accounted for by the assessment of: (i) within‐study bias, (ii) reporting bias, (iii) indirectness, (iv) imprecision, (v) heterogeneity, and (vi) incoherence.^(^ ^29^ ^)^ Conventional levels of HR (0.8, 1.25) and mean difference (MD) 2.71 (1/2 SD of baseline control arms) were used to indicate clinical significance for fractures and BMD outcomes, respectively. The assessment of credibility of findings was conducted using CINeMA's freely available web application.^(^ ^30^ ^)^

### Additional analyses

Sensitivity analysis was conducted on the main outcomes (vertebral and nonvertebral fractures and BMD at femoral neck). Studies with an overall high risk of bias, studies in which patients were switched to different treatment doses, and a single study that was an independent substudy of an included trial were excluded in the sensitivity analysis of vertebral and nonvertebral fractures. For BMD outcome, two sensitivity analyses were conducted. The first sensitivity analysis assessed the comparative effectiveness of bisphosphonates after excluding those studies with an overall high‐risk rating in the risk of bias assessment and the one study that was an independent substudy of an included trial. The second sensitivity analysis was conducted after excluding those studies in which BMD data was extracted from graphs.

Heterogeneity in treatment effects was explored by considering potential treatment effect modifiers.^(^ ^13^ ^)^ A set of subgroup meta‐regressions were conducted on the main outcomes, testing the effects of the following three covariates: (i) proportion of patients with osteoporosis ≥75%, (ii) proportion of patients with increased risk of fractures ≥75%, and (iii) mode of administration (oral versus intravenous). In all subgroup analyses, we assumed a common interaction effect that applies to relative effects of all the treatments relative to the reference treatment one.^(^ ^13^ ^)^ For BMD changes, study duration was included in meta‐regression as a trial‐level continuous covariate (centered). For both fractures and BMD outcomes, additional meta‐regressions were run, adjusting for participants' baseline‐risk, where the interaction term indicates the change in the treatment effect (eg, log‐HR for fracture data and change in mean difference between treatments for BMD data) per unit change in the baseline risk/response.

## Results

### Study selection

**Fig. 1:**
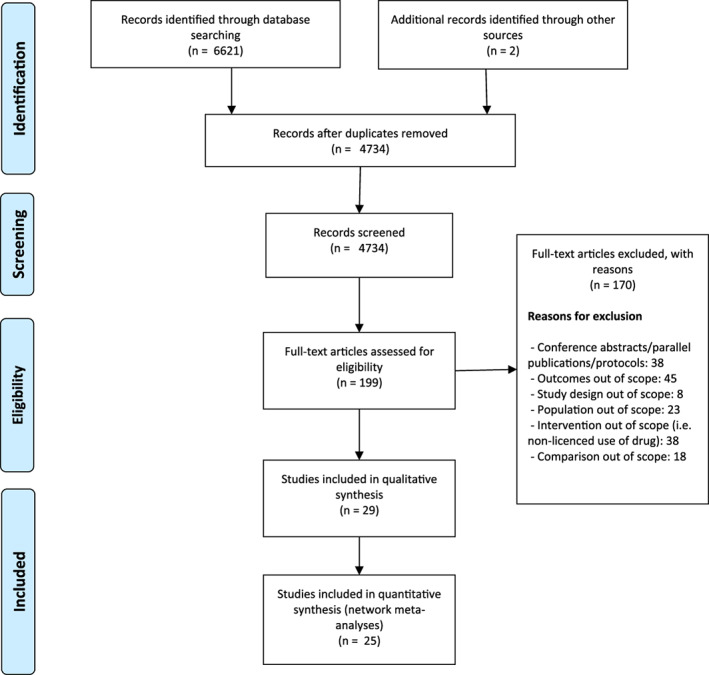
PRISMA flow diagram of the selected studies in the updated review. PRISMA = Preferred Reporting Items for Systematic reviews and Meta‐Analyses.

A PRISMA flow diagram shows the selection of papers for inclusion and exclusion in the updated systematic review (Fig. 1). A total of 6623 articles were retrieved, of which 1889 were duplicates. Overall, 4535 studies were excluded following title and abstract screening, and 170 were excluded following full‐text screen (Appendix 10). Data from 25 newly identified trials obtained from 29 published reports were added to the data obtained from 43 trials identified in the previous review,^(^ ^3^ ^)^ resulting in a total of 68 trials of 47,007 participants (Appendices 9 & 11).

### Network structures and geometry

Network graphs comparing bisphosphonates for the prevention of fragility fractures are presented for all outcomes (Appendix 3). Four networks were created for fractures data. Data for vertebral and hip fractures provided us with one closed loop of evidence. Data for nonvertebral fractures did not provide us with a closed loop of evidence, and the indirect effects were drawn from a single study. Similarly, data for wrist fractures provided us with a single loop after removing the only three‐arm study of the network. Data for BMD provided us with five closed loops after removing the single three‐arm study, whereas three of the loops were accounted for by single studies. A total of 28,340 (nstudies = 27) participants received bisphosphonates (ntreatments = 5) to prevent vertebral fractures. The most commonly studied treatments were ZOL (*n* = 10) and RIS (*n* = 10). Placebo was used as the comparator arm in 24 studies. The most frequently used comparisons were ZOL versus placebo (*n* = 9) and RIS versus placebo (*n* = 8). A total of 26,435 (nstudies = 19) received bisphosphonates (ntreatments = 5) for preventing nonvertebral fractures. The drug that was more commonly studied was ZOL (*n* = 7). Placebo was used as the comparator arm in 18 studies. The most commonly studied comparisons were ZOL versus placebo (*n* = 7) and ALN versus placebo (*n* = 6). A total of 28,570 (nstudies = 44) participants received bisphosphonates (ntreatments = 5) providing us with data for femoral neck BMD. Data was drawn from 43 two‐arm studies and one three‐arm study. The studied medications were more commonly ALN (nstudies = 23) and RIS (nstudies = 16). Placebo was used as the comparator arm in 37 studies. The most commonly studied comparisons were ALN versus placebo (*n* = 17 studies) and RIS versus placebo (*n* = 11 studies). No trials testing IBN‐iv against any of the aforementioned bisphosphonates were identified.

### Characteristics of studies and risk of bias within individual studies

Twenty‐five new trials of 6318 participants were identified from 29 published reports, covering the period from 2014 to 2021. Overall, 10 studies were conducted in China,^(^ ^28^, ^31^, ^32^, ^33^, ^34^, ^35^, ^36^, ^37^, ^38^, ^39^ ^)^ five studies were conducted in Europe,^(^ ^27^, ^40^, ^41^, ^42^, ^43^ ^)^ three were conducted in the United States,^(^ ^44^, ^45^, ^46^ ^)^ three were conducted in Oceania,^(^ ^47^, ^48^, ^49^ ^)^ one in Japan,^(^ ^50^ ^)^ one in South Korea,^(^ ^51^ ^)^ and two were conducted internationally.^(^ ^52^, ^53^ ^)^ Four extensions of original trials^(^ ^54^, ^55^, ^56^, ^57^ ^)^ and one ancillary substudy of a main trial^(^ ^43^ ^)^ were available, accounting for the total number of eligible studies identified. In two cases,^(^ ^40^, ^47^ ^)^ trials published before 2014 were deemed eligible for inclusion and included in the updated review after receiving clinicians' feedback. The sample sizes of the trials identified in the updated review ranged from 30 to 2000 participants. A full list of included studies' characteristics are reported in Appendix 2. Overall, 19 trials recruited exclusively female participants.^(^ ^27^, ^28^, ^32^, ^34^, ^36^, ^37^, ^38^, ^40^, ^41^, ^42^, ^43^, ^44^, ^45^, ^46^, ^47^, ^49^, ^51^, ^52^, ^53^ ^)^ In nine trials, most of participants had received a diagnosis of osteoporosis before entering the study,^(^ ^28^, ^31^, ^32^, ^33^, ^34^, ^36^, ^37^, ^41^, ^43^ ^)^ participants in nine trials fulfilled the criteria for secondary causes of osteoporosis,^(^ ^28^, ^41^, ^42^, ^45^, ^46^, ^48^, ^51^, ^53^, ^54^ ^)^ participants in four trials received the treatments of interest postoperation,^(^ ^31^, ^33^, ^35^, ^37^ ^)^ whereas the majority of participants had a history of fractures or were recruited on the basis of fractures at baseline in six trials.^(^ ^32^, ^33^, ^35^, ^37^, ^50^, ^54^ ^)^ Overall, 15 trials identified in the updated review provided us with data regarding the occurrence of fractures,^(^ ^27^, ^31^, ^32^, ^33^, ^37^, ^38^, ^39^, ^43^, ^45^, ^47^, ^48^, ^49^, ^50^, ^51^, ^54^ ^)^ whereas 13 trials provided data regarding percentage BMD change at femoral neck^(^ ^27^, ^28^, ^36^, ^38^, ^40^, ^43^, ^44^, ^45^, ^46^, ^50^, ^51^, ^52^, ^54^ ^)^ and three provided data regarding absolute BMD changes^(^ ^33^, ^34^, ^41^ ^)^ (Appendix 2). All but two of the newly identified trials^(^ ^36^, ^41^ ^)^ reported prevalence of adverse events (Appendix 2). In total, the overall risk of bias was high in 12 trials^(^ ^27^, ^31^, ^32^, ^33^, ^35^, ^37^, ^38^, ^40^, ^41^, ^44^, ^51^, ^53^ ^)^ (Appendix 6). Most of the high‐risk ratings were observed in the “blinding of participants and personnel” and “incomplete outcome data” domains.

### Synthesis of results on the main outcomes

#### Primary outcome: vertebral fractures

Data were available from 27 RCTs (Appendix 3). The network provided six direct treatment comparisons. Three contrasts were checked for inconsistency with none of the comparisons showing significant evidence of inconsistency (*p* > 0.1) (Appendix 8). The model fitted the data relatively well (data points: 54; total residual deviance [Dres]: 56.34; deviance information criterion [DIC]: 298.5). The between‐study SD was estimated to be 0.18 (95% CrI, 0.01–0.46), whereas the between‐treatment SD was estimated to be 0.19 (95% CrI, 0.01–0.46). All treatments were associated with beneficial treatment effects relative to placebo and all treatment effects were statistically significant (*p* < 0.05) (Table 1). ZOL, ALN, and RIS were also found to exert clinically significant effects. ZOL was associated with the greatest effect (HR 0.38; 95% CrI, 0.28–0.49) and it was most likely to be the most effective treatment (probability: 0.55) (Appendix 4).

**Table 1:** League Table Presenting Network Meta‐Analysis Estimates (Lower Triangle) and Direct Estimates (Upper Triangle) of Efficacy of Bisphosphonates

(i) %BMD change at femoral neck					(ii) Vertebral fractures					(iii) Nonvertebral fractures				
ZOL				3.8 (2.7, 4.8)	ZOL				0.33 (0.23, 0.43)	RIS				–
1.15 (0.24, 2.08)	ALN			3.1 (2.4, 3.8)	0.88 (0.58, 1.21)	ALN			0.43 (0.33, 0.53)	0.98 (0.82, 1.35)	ZOL			–
1.31 (−0.08, 2.73)	0.15 (−1, 1.32)	IBNor		2.3 (0.21, 4.3)	0.87 (0.37, 1.82)	0.99 (0.47, 2.18)	IBNor		–	0.95 (0.5, 1.33)	0.98 (0.55, 1.36)	IBNor		–
1.76 (0.82, 2.74)	0.6 (−0.09, 1.31	0.45 (−0.8, 1.72)	RIS	2.4 (1.5, 3.3)	0.76 (0.5, 1.07)	0.88 (0.6, 1.22)	0.91 (0.37, 1.82)	RIS	0.54 (0.39, 0.69)	0.92 (0.65, 1.11)	0.93 (0.74, 1.11)	0.99 (0.63. 1.5)	ALN	–
4.02 (3.2, 4.84)	2.86 (2.37, 3.36)	2.7 (1.56, 3.86)	2.25 (1.61, 2.87)	PLB	0.38 (0.28, 0.49)	0.44 (0.33, 0.57)	0.44 (0.2, 0.94)	0.5 (0.37, 0.66)	PLB	0.70 (0.53, 0.84)	0.71 (0.61, 0.81)	0.75 (0.51, 1.26)	0.77 (0.63, 0.91)	PLB

From left to right: (i) % BMD change at femoral neck, (ii) vertebral fractures, and (iii) nonvertebral fractures. Posterior mean differences (95% CI) are presented for percentage BMD change at femoral neck and posterior median HRs (95% CI) for vertebral and nonvertebral fractures. Treatments are reported in order of relative ranking for efficacy. Comparisons between treatments should be read from left to right, and their HR is in the cell in common between the column‐defining treatment and the row‐defining treatment. HRs <1 favor the column‐defining treatment for the network estimates and the row‐defining treatment for the direct estimates.

ALN = alendronate; HR = hazard ratio; IBNor = ibandronate 150 mg; PLB = placebo; RIS = risedronate; ZOL = zoledronate.

#### Outcome: nonvertebral fractures

Data were available from 19 RCTs (Appendix 3). The model fitted the data well (data points: 38; Dres: 28.57; DIC: 224.8). The between‐study SD was estimated to be 0.08 (95% CrI, 0.06–0.24), whereas the between‐treatment SD was estimated to be 0.21 (95% CrI, 0.005–0.99). All treatments were associated with beneficial treatment effects relative to placebo, with RIS, ALN, and ZOL being statistically significant (*p* < 0.05) (Table 1). RIS was associated with the greatest effect (HR 0.7; 95% CrI, 0.53–0.84) and was most likely to be the most effective treatment (probability: 0.44) (Appendix 4). ZOL was found to be comparably effective, showing more precise effects (HR 0.71; 95% CrI, 0.61–0.81).

#### Primary outcomes: hip fractures and wrist fractures

Data on the occurrence of hip fractures were available from 14 RCTs. The model fitted the data well (data points: 28; Dres: 22.22; DIC: 144.8). The between‐study SD was estimated to be 0.1 (95% CrI, 0–0.33), whereas the between‐treatment SD was estimated to be 0.36 (95% CrI, 0–1.8). All treatments were associated with beneficial treatment effects relative to placebo, whereas ZOL, ALN, and RIS were found to exert statistically significant treatment effects (*p* < 0.05). ZOL (HR 0.61; 95% CrI, 0.47–0.79) and ALN (HR 0.61; 95% CrI, 0.4–0.86) were associated with the greatest effects, with the effects of the former being clinically significant.

Data on the occurrence of wrist fractures were available from 10 RCTs with one RCT comparing three treatments. The model fitted the data well (data points: 21; Dres: 21.83; DIC: 95.26). The between‐study SD was estimated to be 0.29 (95% CrI, 0–0.68), whereas the between‐treatment SD was estimated to be 0.44 (95% CrI, 0.01–1.8). All treatments were associated with beneficial treatment effects relative to placebo, although the treatment effects were not statistically significant (*p* > 0.05). ZOL was associated with the greatest effect, with HR 0.54 (95% CrI, 0.04–1.36), and was most likely to be the most effective treatment (probability: 0.47) (Appendix 4).

#### Primary outcome: percentage change in femoral neck BMD

Data were available from 44 RCTs with one RCT comparing three treatments.^(^ ^27^ ^)^ The model's fit with the data was moderate (data points: 89; Dres: 92.21; DIC: 173.4), whereas none of the seven comparisons showed significant evidence of inconsistency (*p* > 0.1) (Appendix 8). The between‐study SD was 0.93 (95% CrI, 0.64–1.34). The interaction term for duration of study was 0.78 (95% CrI, 0.3–1.24), implying that longer study duration predicts BMD increases for treatment arms. All treatments were associated with beneficial effects relative to placebo (Table 1), and all treatment effects were statistically significant (*p* < 0.05). ZOL was associated with the greatest effect (MD 4.02; 95% CrI, 3.2–4.84), and was most likely to be the most effective treatment (probability: 0.96; SUCRA %: 99) (Appendix 4). ZOL was also found to exert clinically‐significant effects. Additional analysis was performed on BMD data by undertaking two separate NMAs for 12‐month and 24‐month to 36‐month data (Appendix 5). Both models fitted the data well with ZOL being the most effective treatment at both time points (MD 12‐month: 3.05; 95% CrI, 2.25–3.85, *p* < 0.05; MD 24‐36 months: 4.11; 95% CrI, 2.84–5.52, *p* < 0.05). In those studies where BMD changes were reported as absolute difference from baseline,^(^ ^33^, ^34^, ^41^ ^)^ statistically significant increases in BMD at femoral neck were observed in treatment groups at 12‐month follow‐up.

### Outline of results on the secondary outcomes

Eleven NMAs were conducted on secondary outcomes (Appendix 5). ZOL was found to be significantly worse compared to placebo on overall adverse events (HR 1.52; 95% CrI, 1.19–1.96), arthralgia (HR 1.95; 95% CrI, 1.17–3.01), headache (HR 2.76; 95% CrI, 2.32–3.29), influenza‐like symptoms (HR 6.05; 95% CrI, 3.07–10.86), myalgia (HR 5.21; 95% CrI, 4.35–6.3), and pyrexia symptoms (HR 9.37; 95% CrI, 7.11–15.56). The model fit with the data was: poor on overall adverse‐events outcome (Dres: 91.23; data points: 77), good on arthralgia outcome (Dres: 31.98; data points: 32), moderate on headache outcome (Dres: 25.46; data points: 22), poor on influenza‐like symptoms outcome (Dres: 35.93; data points: 24), relatively good on myalgia outcome (Dres: 24.69; data points: 22), and moderate on pyrexia outcome (Dres: 27.27; data points: 24). Additional information regarding the analysis of secondary outcomes is provided in Appendix 5.

### Risk of bias across studies and credibility of findings

Risk of bias assessment at outcome level was undertaken for all studies conferring data to vertebral fractures and BMD. For vertebral fractures, most of major concerns were detected in the comparisons of RIS versus placebo (>70%) and ALN versus RIS (>40%) with the former being informed by eight direct comparisons and the latter by one direct comparison (Appendix 7). From mixed‐treatment comparisons, findings drawn from two treatment‐placebo comparisons were rated as highly credible (ALN versus placebo; ZOL versus placebo). Findings drawn from RIS versus placebo and RIS versus ZOL comparisons were considered of moderate credibility, with the latter being informed by only one direct pairwise comparison. Findings drawn from ALN versus IBNor and ALN versus RIS comparisons were considered of low credibility with the former comparison being informed by a small study of zero events in the control group. From indirect comparisons, evidence drawn from the treatment‐placebo comparison (placebo versus IBN‐oral) and one active comparison (ALN versus ZOL) were both rated as highly credible, whereas the rest of indirect comparisons produced evidence of low credibility.

For percentage BMD change, most of major concerns were detected in the active comparison of ALN versus RIS (marginally >10%) with four studies providing evidence (Appendix 7). Proportion of evidence drawn from studies with major concerns were <10% in the rest of comparisons. Apart from two active comparisons (ALN versus ZOL; IBNor versus ZOL), all the comparisons provided us with highly credible findings. With regard to the two comparisons providing us with evidence of low credibility, the direct evidence for the comparison of ALN versus ZOL were drawn from a single, outlier study.^(^ ^28^ ^)^

### Results of additional analysis

Heterogeneity of effects was explored by undertaking separate sensitivity analyses for each of the main outcomes and using risk of bias assessment as a moderator variable (Appendix 5). For vertebral fractures, data were available from 22 two‐arm studies. The model had a good fit with the data with a total residual deviance of 43.47 (data points: 44). The between‐study SD was estimated to be 0.23 (95% CrI, 0.01–0.53), implying mild heterogeneity in treatment effects between RCTs. The direction of the findings remained the same compared to the main analysis whereas only minimal differences were detected in the magnitude of observed effects. All treatment effects were different compared to placebo (*p* < 0.05). ZOL was found to have the most beneficial effects compared to placebo (HR 0.41; 95% CrI, 0.3–0.55). For nonvertebral fractures, data were available from 16 two‐arm studies. The model had a good fit with the data with a total residual deviance of 23.96 (total number of data points: 32). The between‐study SD was estimated to be 0.08 (95% CrI, 0.004–0.24), implying only minimal heterogeneity in treatment effects between RCTs. The direction of findings remained the same compared to the main analysis whereas the larger deviations were detected in the observed effect sizes of ALN and IBN‐oral. Similar to the main analysis, only the treatment effects related to IBN‐oral were not statistically significant compared to placebo (*p* > 0.05). RIS was found to have the most beneficial effects compared to placebo (HR 0.64; 95% CrI, 0.42–0.84). For percentage BMD change, data were available from 33 two‐arm studies (Appendix 5). The model had a good fit with the data with a total residual deviance of 61.49 (data points: 66). The between‐study SD was estimated to be 0.75 (95% CrI, 0.5–1.09), implying high heterogeneity in treatment effects between RCTs with reasonable uncertainty. The direction of the findings remained the same compared to the main analysis and all treatment effects were statistically significant compared to placebo (*p* < 0.05). ZOL was found to have the most beneficial effects compared to placebo (MD 3.69; 95% CrI, 2.91–4.45). Additional information regarding sensitivity analyses are provided in Appendix 5.

Heterogeneity was also explored by undertaking a set of four meta‐regressions on the main fracture outcomes (Appendix 5). None of the tested effect modifiers were found to significantly interact with the treatment effects apart from participants' osteoporotic status on vertebral fractures. For vertebral fractures, the model fit of the meta‐regression on the osteoporotic status of participants was good with a total residual deviance of 52.59 (data points: 54). The between‐study SD was estimated to be 0.12 implying mild heterogeneity in treatment effects between RCTs. Treatment effects were found to vary according to the type of participants, with larger treatment effects found to be associated with osteoporotic status, providing an interaction term of −0.61 (95% CrI, −1.07 to −0.17). The model fit was improved by including participants' osteoporosis status as an effect modifier. Additional information regarding subgroup analyses are provided in Appendix 5.

## Discussion

This is an update of a systematic review that was previously published as part of a NICE HTA report. Overall, 44 trials provided data for femoral neck BMD, whereas 27 and 19 trials provided data for vertebral and nonvertebral fractures, respectively. Only 14 and 10 trials provided data for hip and wrist fractures, respectively. ZOL was found to be the most effective treatment in preventing the occurrence of vertebral fractures and increasing femoral neck BMD. ZOL was also found to be comparably effective to RIS and ALN in preventing nonvertebral fractures and hip fractures respectively. ZOL's effects in preventing hip and vertebral fractures, and increasing femoral neck BMD were found to be clinically significant. In addition, treatment effects in preventing vertebral fractures were found to be stronger in people with osteoporosis compared to placebo. Uptake of ZOL was also found to be accompanied by more frequently reported adverse events; however, these events are likely to be short‐lived. Based on these updated estimates, ZOL could be considered as the first‐line treatment for people who experience or are at increased risk of fragility fractures.

These findings arguably have important implications for clinical decision‐making in terms of the preferred therapeutic approach for people with varying fracture risk. It has recently been suggested that anabolic treatments should be preferred as the first‐line treatment for people who are at high risk for developing osteoporotic fractures.^(^ ^58^ ^)^ Although recent evidence has shown that anabolic treatment is more effective than bisphosphonates in reducing fracture risk in females who are at high risk to develop fractures,^(^ ^59^, ^60^ ^)^ their effectiveness has only been tested against oral bisphosphonates. There is an urgent need therefore, for future comparative studies to test the effectiveness of anabolic treatments versus ZOL in reducing the fracture risk in high‐risk populations. This becomes more apparent when the imminent fracture risk and the need to expedite clinical decision‐making^(^ ^61^, ^62^ ^)^ are taken into account. Based on our findings, ZOL seems a promising treatment that could decrease the imminent fracture risk for high‐risk populations within 24 months after administration. Future studies should investigate whether ZOL or anabolic treatments are more effective in reducing imminent fracture risk in high‐risk populations.

### Strengths and limitations

These network meta‐analyses provide updated estimates regarding bisphosphonates' effect in preventing the occurrence of fractures. This updated systematic review has several strengths. First, this review includes a robust search strategy with clearly‐demarcated eligibility criteria, covering a wide range of databases, trial registries, and gray literature. Second, this review employed gold‐standard methods in analyzing, reporting, and assessing the quality of findings, which in turn facilitates clinical decision‐making. Inevitably, this review has also some limitations. First, treatment networks for hip and wrist fractures were sparse, something that might limit the generalization of our conclusions regarding bisphosphonates' effects on those outcomes. Second, none of the included studies had tested IBN‐iv against any other bisphosphonate or placebo, preventing the provision of updated estimates regarding IBN‐iv effectiveness. Third, there was scarcity of data regarding bisphosphonates' effects on male populations and populations with exposure to glucocorticoids.

## Conclusions

ZOL was found to be the most effective bisphosphonate compared to ALN, RIS, and IBN‐oral for reducing the risk of fragility fracture. Depending on its cost‐effectiveness, ZOL could be considered as a first‐line option for people at increased risk of subsequent fractures.

## Author contributions

**Anastasios Bastounis:** Conceptualization; formal analysis; investigation; methodology; project administration; writing – original draft; writing – review and editing. **Tessa Langley:** Conceptualization; funding acquisition; investigation; methodology; project administration; writing – review and editing. **Sarah Davis:** Conceptualization; funding acquisition; investigation; methodology; writing – review and editing. **Zoe Paskins:** Conceptualization; funding acquisition; investigation; writing – review and editing. **Neil Gittoes:** Conceptualization; funding acquisition; investigation; writing – review and editing. **Jo Leonardi‐Bee:** Conceptualization; formal analysis; funding acquisition; investigation; methodology; project administration; writing – review and editing. **Opinder Sahota:** Conceptualization; funding acquisition; investigation; project administration; writing – review and editing.

## Conflicts of interest

All other authors have nothing to declare.

### Peer review

The peer review history for this article is available at https://publons.com/publon/10.1002/jbm4.10620.

## Supporting information


File S1
Appendix 1
Appendix 2
Appendix 3
Appendix 4
Appendix 5
Appendix 6
Appendix 7
Appendix 8
Appendix 9
Appendix 10
Appendix 11
Appendix 12

## Data Availability

Data are from published research and therefore are mostly in the public domain. Extracted data are provided in the appendices.
