# Supplementary material for: Assessing the Effectiveness of Bisphosphonates for the Prevention of Fragility Fractures: An Updated Systematic Review and Network Meta‐Analyses
Source: JBMR Plus. 2022 Mar 25;6(5):e10620. doi: 10.1002/jbm4.10620 (PMC9059468; doi:10.1002/jbm4.10620)
Supplement: Supplementary file 1 — File S1 Appendix 1 Appendix 2 Appendix 3 Appendix 4 Appendix 5 Appendix 6 Appendix 7 Appendix 8 Appendix 9 Appendix 10 Appendix 11 Appendix 12 [file JBM4-6-e10620-s001.docx]

**Supplementary appendix**

Title: Assessing the effectiveness of bisphosphonates for the prevention of fragility fractures: an updated systematic review and network meta-analyses.

Authors’ list: Bastounis, A., Langley, T., Davis, S., Paskins, Z., Gittoes, N., Leonardi-Bee, J., & Sahota, O.

**CONTENTS**

Appendix 1. Search strategy

Appendix 2. Studies characteristics tables

Appendix 3. Network plots

Appendix 4. Treatment ranking probabilities

Appendix 5. Analysis of hip & wrist fractures, analysis of secondary outcomes, sensitivity analysis and meta-regressions

Appendix 6. Risk of bias assessment

Appendix 7. Quality of evidence

Appendix 8. Assessment of inconsistency

Appendix 9. Data used to populate the network meta-analyses of the main outcomes

Appendix 10. List of the excluded studies

Appendix 11. References of studies drawn from the original review

Appendix 12. PRISMA NMA Checklist

**Appendix 1: Search Strategy**

Database: [Ovid MEDLINE(R)](https://nottingham-uk.userservices.exlibrisgroup.com/view/action/uresolver.do;jsessionid=31D2470FFBB009AADA514CC483C0A4A4.app01.eu00.prod.alma.dc03.hosted.exlibrisgroup.com:1801?operation=resolveService&package_service_id=5823275300005561&institutionId=5561&customerId=5560) (date range: 2014-2021)

Date of original search: 1 April 2020; Date of the updated search: 8 February 2021; Date of the final scoping search (MEDLINE, PubMed, EMBASE): 01 March 2021

**Table 2. Main search strategy (database: Medline)**

| **Searches** | **Search terms** |
| --- | --- |
| #1 | exp osteoporosis/ |
| #2 | osteoporo$.tw. |
| #3 | bone diseases, metabolic/ |
| #4 | exp Bone Density/ |
| #5 | (bone adj3 densit$).tw. |
| #6 | exp fractures, bone/ |
| #7 | fractures, cartilage/ |
| #8 | fracture$.ti,ab. |
| #9 | (bone$ adj2 fragil$).tw. |
| #10 | bone mineral densit$.tw. |
| #11 | bone loss.tw. |
| #12 | bmd.tw. |
| #13 | or/1-12 |
| #14 | (alendron$ or fosomax or fosavance).mp. |
| #15 | 121268-17-5.rn. |
| #16 | (ibandron$ or boniva or bondronat or bonviva or adronil).mp. |
| #17 | 114084-78-5.rn. |
| #18 | (risedron$ or actonel or atelvia or benet).mp. |
| #19 | 105462-24-6.rn. |
| #20 | (zoledron$ or zometa or zomera or aclasta or reclast).mp. |
| #21 | 118072-93-8.rn. |
| #22 | or/14-21 |
| #23 | 13 and 22 |
| #24 | Randomized controlled trials as Topic/ |
| #25 | Randomized controlled trial/ |
| #26 | Random allocation/ |
| #27 | randomized controlled trial.pt. |
| #28 | Double blind method/ |
| #29 | Single blind method/ |
| #30 | Clinical trial/ |
| #31 | exp Clinical Trials as Topic/ |
| #32 | controlled clinical trial.pt. |
| #33 | clinical trial$.pt. |
| #34 | multicenter study.pt. |
| #35 | or/24-34 |
| #36 | (clinic$ adj25 trial$).ti,ab. |
| #37 | ((singl$ or doubl$ or treb$ or tripl$) adj (blind$ or mask$)).tw. |
| #38 | Placebos/ |
| #39 | Placebo$.tw. |
| #40 | (allocated adj2 random).tw. |
| #41 | or/36-40 |
| #42 | 35 or 41 |
| #43 | Case report.tw. |
| #44 | Letter/ |
| #45 | Historical article/ |
| #46 | 43 or 44 or 45 |
| #47 | exp Animals/ |
| #48 | Humans/ |
| #49 | 47 not (47 and 48) |
| #50 | 46 or 49 |
| #51 | 42 not 50 |
| #52 | 23 and 51 |
| #53 | limit 52 to yr=“2014 –Current” |
| #54 | meta-analysis as topic/ |
| #55 | (meta analy$ or metaanaly$).tw. |
| #56 | Meta-Analysis/ |
| #57 | (systematic adj (review$1 or overview$1)).tw. |
| #58 | “Review Literature as Topic”/ |
| #59 | or/54-58 |
| #60 | (cochrane or embase or psychlit or psyclit or psychinfo or psycinfo or cinahl or cinhal or science  citation index or bids or cancerlit).ab. |
| #61 | ((reference adj list$) or bibliograph$ or hand-search$ or (relevant adj journals) or (manual adj  search$)).ab. |
| #62 | ((selection adj criteria) or (data adj extraction)).ab. |
| #63 | “review”/ |
| #64 | 62 and 63 |
| #65 | comment/ or editorial/ or letter |
| #66 | Animals/ |
| #67 | Humans/ |
| #68 | 66 not (66 and 67) |
| #69 | 65 or 68 |
| #70 | 59 or 60 or 61 or 64 |
| #71 | 70 not 69 |
| #72 | 23 and 71 |
| #73 | limit 72 to yr=“2014 –Current” |

**Table 3. Supplementary search strategy for adverse events (database: Medline)**

| **searches** | **Search terms** |
| --- | --- |
| #1 | (alendron$ or fosomax or fosavance).mp. |
| #2 | 121268-17-5.rn. |
| #3 | (ibandron$ or boniva or bondronat or bonviva or adronil).mp. |
| #4 | 114084-78-5.rn. |
| #5 | (risedron$ or actonel or atelvia or benet).mp. |
| #6 | 105462-24-6.rn. |
| #7 | (zoledron$ or zometa or zomera or aclasta or reclast).mp. |
| #8 | 118072-93-8.rn. |
| #9 | or/1-8 |
| #10 | (ae or to or po or co).fs. |
| #11 | (safe or safety).ti,ab. |
| #12 | side effect$.ti,ab. |
| #13 | ((adverse or undesirable or harms$ or serious or toxic) adj3 (effect$ or reaction$ or event$ or outcome  $)).ti,ab. |
| #14 | (toxicity or complication$ or noxious or tolerability).ti,ab. |
| #15 | or/10-14 |
| #16 | 9 and 15 |
| #17 | MEDLINE.tw. |
| #18 | systematic review.tw. |
| #19 | meta analysis.pt. |
| #20 | or/17-19 |
| #21 | 16 and 20 |

**Table 4. Supplementary search strategy for Quality of Life (QoL) (database: Medline)**

| **Searches** | **Search terms** |
| --- | --- |
| 1# | exp osteoporosis/ |
| 2# | bone diseases, metabolic/ |
| 3# | osteoporo$.tw. |
| 4# | or/1-3 |
| 5# | (bone adj6 densit$).tw. |
| 6# | bone density/ |
| 7# | bmd.ti,ab. |
| 8# | (bone or bones).mp. |
| 9# | exp densitometry/ |
| 10# | tomography, x-ray computed/ |
| 11# | densit$.tw. |
| 12# | 10 and 11 |
| 13# | 9 or 12 |
| 14# | 8 and 13 |
| 15# | 5 or 6 or 7 or 14 |
| 16# | exp fractures, bone/ |
| 17# | fractures, cartilage/ |
| 18# | fracture$.ti,ab. |
| 19# | or/16-18 |
| 20# | 15 or 19 |
| 21# | 4 and 20 |
| 22# | (euroqol or euro qol or eq5d or eq 5d).mp. |
| 23# | 21 and 22 |
| 24# | limit 23 to yr=“2014 –Current” |

**Table 5. Supplementary search strategy (database: PubMed)**

| Database: PubMed | |
| --- | --- |
|  | ((osteoporosis[mesh major topic]) OR (fractures[mesh major topic])) AND (osteoporosis [tiab] or osteopenia [tiab] or osteoporo* [tiab] or osteopeni* [tiab] or osteopaen* [tiab] or fragil* [tiab] or fractures [tiab] or bone density [tiab] or bmd [tiab] bone density conservation agents [tiab] or bisphosphon* [tiab] or alendron* [tiab] or fosamax [tiab] or ibandron* [tiab] or avlasta [tiab] or zoledron* [tiab] or risedron* [tiab] or actonel [tiab] or etidron* [tiab] or pamidron* [tiab] or zometa [tiab] or zomer a [tiab] or boneva [tiab] or bonviva [tiab] or bondronat [tiab] or fosavance [tiab] or andronic [tiab] or bisphosphonates [tiab] or alendronate [tiab] or pamidronate [tiab] or ibandronate [tiab]) |

**Table 6. Main search strategy (database: EMBASE)**

| **Searches** | **Search terms** |
| --- | --- |
| #1 | exp osteoporosis/ |
| #2 | osteoporo$.tw. |
| #3 | bone diseases, metabolic/ |
| #4 | exp Bone Density/ |
| #5 | (bone adj3 densit$).tw. |
| #6 | exp fractures, bone/ |
| #7 | fractures, cartilage/ |
| #8 | fracture$.ti,ab. |
| #9 | (bone$ adj2 fragil$).tw. |
| #10 | bone mineral densit$.tw. |
| #11 | bone loss.tw. |
| #12 | bmd.tw. |
| #13 | or/1-12 |
| #14 | (alendron$ or fosomax or fosavance).mp. |
| #15 | 121268-17-5.rn. |
| #16 | (ibandron$ or boniva or bondronat or bonviva or adronil).mp. |
| #17 | 114084-78-5.rn. |
| #18 | (risedron$ or actonel or atelvia or benet).mp. |
| #19 | 105462-24-6.rn. |
| #20 | (zoledron$ or zometa or zomera or aclasta or reclast).mp. |
| #21 | 118072-93-8.rn. |
| #22 | or/14-21 |
| #23 | 13 and 22 |
| #24 | Randomized controlled trials as Topic/ |
| #25 | Randomized controlled trial/ |
| #26 | Random allocation/ |
| #27 | randomized controlled trial.pt. |
| #28 | Double blind method/ |
| #29 | Single blind method/ |
| #30 | Clinical trial/ |
| #31 | exp Clinical Trials as Topic/ |
| #32 | controlled clinical trial.pt. |
| #33 | clinical trial$.pt. |
| #34 | multicenter study.pt. |
| #35 | or/24-34 |
| #36 | (clinic$ adj25 trial$).ti,ab. |
| #37 | ((singl$ or doubl$ or treb$ or tripl$) adj (blind$ or mask$)).tw. |
| #38 | Placebos/ |
| #39 | Placebo$.tw. |
| #40 | (allocated adj2 random).tw. |
| #41 | or/36-40 |
| #42 | 35 or 41 |
| #43 | Case report.tw. |
| #44 | Letter/ |
| #45 | Historical article/ |
| #46 | 43 or 44 or 45 |
| #47 | exp Animals/ |
| #48 | Humans/ |
| #49 | 47 not (47 and 48) |
| #50 | 46 or 49 |
| #51 | 42 not 50 |
| #52 | 23 and 51 |
| #53 | limit 52 to yr=“2014 –Current” |
| #54 | meta-analysis as topic/ |
| #55 | (meta analy$ or metaanaly$).tw. |
| #56 | Meta-Analysis/ |
| #57 | (systematic adj (review$1 or overview$1)).tw. |
| #58 | “Review Literature as Topic”/ |
| #59 | or/54-58 |
| #60 | (cochrane or embase or psychlit or psyclit or psychinfo or psycinfo or cinahl or cinhal or science  citation index or bids or cancerlit).ab. |
| #61 | ((reference adj list$) or bibliograph$ or hand-search$ or (relevant adj journals) or (manual adj  search$)).ab. |
| #62 | ((selection adj criteria) or (data adj extraction)).ab. |
| #63 | “review”/ |
| #64 | 62 and 63 |
| #65 | comment/ or editorial/ or letter |
| #66 | Animals/ |
| #67 | Humans/ |
| #68 | 66 not (66 and 67) |
| #69 | 65 or 68 |
| #70 | 59 or 60 or 61 or 64 |
| #71 | 70 not 69 |
| #72 | 23 and 71 |
| #73 | limit 72 to yr=“2014 –Current” |

**Table 7. Supplementary search – Adverse events (database: EMBASE)**

| **searches** | **Search terms** |
| --- | --- |
| #1 | (alendron$ or fosomax or fosavance).mp. |
| #2 | 121268-17-5.rn. |
| #3 | (ibandron$ or boniva or bondronat or bonviva or adronil).mp. |
| #4 | 114084-78-5.rn. |
| #5 | (risedron$ or actonel or atelvia or benet).mp. |
| #6 | 105462-24-6.rn. |
| #7 | (zoledron$ or zometa or zomera or aclasta or reclast).mp. |
| #8 | 118072-93-8.rn. |
| #9 | or/1-8 |
| #10 | (ae or to or po or co).fs. |
| #11 | (safe or safety).ti,ab. |
| #12 | side effect$.ti,ab. |
| #13 | ((adverse or undesirable or harms$ or serious or toxic) adj3 (effect$ or reaction$ or event$ or outcome  $)).ti,ab. |
| #14 | (toxicity or complication$ or noxious or tolerability).ti,ab. |
| #15 | or/10-14 |
| #16 | 9 and 15 |
| #17 | EMBASE.tw. |
| #18 | systematic review.tw. |
| #19 | meta analysis.pt. |
| #20 | or/17-19 |
| #21 | 16 and 20 |

**Table 8. Supplementary search strategy for Quality of Life (QoL) (database: EMBASE)**

| **Searches** | **Search terms** |
| --- | --- |
| 1# | exp osteoporosis/ |
| 2# | bone diseases, metabolic/ |
| 3# | osteoporo$.tw. |
| 4# | or/1-3 |
| 5# | (bone adj6 densit$).tw. |
| 6# | bone density/ |
| 7# | bmd.ti,ab. |
| 8# | (bone or bones).mp. |
| 9# | exp densitometry/ |
| 10# | tomography, x-ray computed/ |
| 11# | densit$.tw. |
| 12# | 10 and 11 |
| 13# | 9 or 12 |
| 14# | 8 and 13 |
| 15# | 5 or 6 or 7 or 14 |
| 16# | exp fractures, bone/ |
| 17# | fractures, cartilage/ |
| 18# | fracture$.ti,ab. |
| 19# | or/16-18 |
| 20# | 15 or 19 |
| 21# | 4 and 20 |
| 22# | (euroqol or euro qol or eq5d or eq 5d).mp. |
| 23# | 21 and 22 |
| 24# | limit 23 to yr=“2014 –Current” |

| **Appendix 2: Studies characteristics tables** | | | | | | | | | |
| --- | --- | --- | --- | --- | --- | --- | --- | --- | --- |
| **Table 9. Studies characteristics** | | | | | | | | | |
| **Studies ID^a^** | **Study design/**  **Country** | **N^b^/**  **Gender (%)/**  **Age(mean, SD)** | **Population/**  **T-score FN, SD (g/cm^2^, baseline)/**  **BMI (mean, SD)** | **% patients with prior and/or baseline incidence of fractures** | **Concomitant medication^c^** | **Drugs** | **Dose/mode/**  **frequency** | **% GC users** | **% patients with (PM) OP** |
| Black et al., 2015  Ext.: Black et al., 2012; Black et al., 2007 | RCT/  International | 190/  F(100)/  ZOL:78(4.71); PLB: 78.1(4.85) | PM/  ZOL: -2.44(0.72); PLB: -2.43(0.6)/  ZOL: 24.6(4.13); PLB: 25(3.98) | P(ZOL: 57.9; PLB: 54.8) | P(hormone  therapy, raloxifene, calcitonin, tibolone, tamoxifen,  dehydroepiandrosterone, ipriflavone, & medroxyprogesterone) | ZOL; PLB | 5mg/IV/annually | NR | P(ZOL:46.3; PLB: 44.2) |
| Cesareo et al., 2015 | RCT/Italy | 30/  F(100)/  ALN(+chol.): 59(5); PLB: 57(5) | PM with hyperparathyroidism/  ALN(+chol.): -2.3(0.2); PLB: -2.3(0.2)/ NR | NR | NR | ALN; PLB | 70mg/oral/weekly | NR | P(100) |
| Cheung et al., 2020 | RCT/Australia | 76/F(0)/ZOL: 68.8(15.57); PLB: 67.5(13.64) | Cancer patients on ADT therapy/NR/ZOL: 28.8(8.79); PLB:27.9(10.04) | NR | ADT therapy with concurrent radiotherapy | ZOL; PLB | 5mg/IV/annually | NP | NP |
| Cosman et al., 2016 | RCT/USA | 175/F(100)/ALN: 66.9 (7.5); PLB: 67.8 (7.8) | PM/  ALN: −2.1 (0.6); PLB: −2.1 (0.6)/  NR | P(NR) | NP | ALN; PLB | 70mg/oral/weekly | NP | P(NR) |
| Eastell et al., 2011  Ext.: Eastell et al., 2014  (OCEAN study) | RCT/European countries | 114/F(100)/NR | PM/NR/ALN: 25.4(3.58); PLB:25.2(3.51) | NP | NR | ALN; PLB | 70mg/oral/weekly | NR | P(82.5) |
| Greenspan, Perrera et al., 2015  (ZEST trial) | RCT/USA | 181/F(100)/ZOL: 85.4(5.66); PLB: 85.5(4.8) | PM with cognitive impairment/  ZOL: -2.3(0.94); PLB: -2.1(0.96)/  ZOL: 28.2(5.66); PLB: 26.9(4.8) | P(ZOL: 52; PLB: 41) | P(Anticonvulsant; glucocorticoids) | ZOL (single infusion in 2 years); PLB | 5mg/IV/once in two year | P(NR) | P(ZOL: 48; PLB: 45) |
| Greenspan, Vujevich et al., 2015 | RCT/USA | 109/F(100)/RIS: 65(7.42); PLB: 64(7.35) | PM with hormone-receptorpositive  breast cancer/  RIS: -1.01(0.11); PLB: -0.95(0.16) /  RIS: 31(3); PLB: 31(3)/ | NR | P (AI: anastrozole: 77%; letrozole:15%; exemestane: 8%) | RIS; PLB | 35mg/oral/weekly | NP | NR |
| Grey et al., 2012  Ext.: Grey et al., 2014; Grey et al., 2017 | RCT/New Zealand | 90/F(100)/ZOL: 66(8) ; PLB: 65(9) | PM osteopenic/NR/ NR | P (ZOL: 6; PLB: 8) | NP | ZOL (single infusion in 5 years);PLB | 5mg/IV/annually | NP | NP |
| Hu et al., 2020 | RCT/China | 242/F(48.34)/ZOL: 62.6(7.2); PLB: 67.45(4.12) | OVCF receiving PVP/NR/ZOL: 26.15(3.21); PLB: 26.79(5.49) | P(NR)/P(100) | NP | ZOL; PLB | 5mg/IV/annually | NP | P(100) |
| Li et al., 2018 | RCT/China | 100/F(57)/ALN: 68.9(6.4); PLB: 68.5(6.6) | OP patients/NR/NR | P(100) | NR | ALN;PLB | 70mg/oral/weekly | NP | P(100) |
| Li et al., 2016 | RCT/China | 60/F(60)/ZOL: 74.99 (4.81); PLB: 73.96 (5.8) | OP patients with hip fracture after receiving internal fixation operation / NR /  ZOL: 25.37 (2.84); PLB: 25.20 (2.61) | P(100) | NP | ZOL; PLB | 5mg/IV/annually | NP | P(100) |
| Liang et al., 2017 | RCT/China | 285/F(100)/ ZOL: 57.22 (2.81); PLB: 57.48 (3.18) | PM osteoporotic/NR / ZOL: 22.65 (1.90); PLB: 23.07 (2.17) | NR | NP | ZOL; PLB | 5mg/IV/annually | NP | P(100) |
| Liu et al., 2019 | RCT/China | 482/F(10.58)/ZOL: 75.41(12.54); PLB: 73.25 (13.75) | Patients with senile  osteoporotic femoral intertrochanteric fractures/NR /ZOL: 23.54 (8.24); PLB: 22.26 (9.55) | P(100) | NR | ZOL; PLB | 5mg/IV/annually | NP | NP |
| Livi et al., 2019 (BONADIUV trial) | RCT/Italy | 171/F(100)/IBN: 60.6(9.57); PLB: 60.5(10.64) | BC patients treated with AI/NR/NR | NP | AI (letrozole: 78%; anastrozole: 20.5%; exemestane: 8.7%) | IBN; PLB | 150mg/oral/monthly | NR | NP |
| McClung et al., 2014 | RCT/ International | 103/F(100)/ALN: 67.1(5.8); PLB: 67(6.5) | PM female/ALN: −1.91(0.61); PLB: −1.76(0.56)/NR | NP | NR | ALN; PLB | 70mg/oral/weekly | NP | P(NR) |
| Nakamura et al., 2017 (ZoNE study) | RCT/Japan | 665/F(93.94)/ZOL: 74 (5.4); PLB: 74.3 (5.4) | OP patients/ZOL: 69.6 with ≤ -2.5; PLB: 71.7 with ≤ -2.5/ZOL: 23.36 (3.22); PLB: 23.26 (3.47) | P(ZOL: 91.2; PLB: 89.4) | NR | ZOL; PLB | 5mg/IV/annually | NR | P(ZOL: 69.6; PLB:71.7) |
| Paggiosi et al., 2014  (TRIO study) | RCT/UK | 172/F(100)/ALN: 67.8 (7.8); IBN: 66.9 (7.2); RIS: 66.8 (6.7) | PM OP/NR/ALN: 25.9 (3.7); IBN: 26.4 (4.0); RIS: 26.8 (3.8) | P(ALN: 23; IBN: 9; RIS: 7) | NR | ALN; IBN; RIS | 70 mg; 150 mg; 35 mg/oral/weekly; monthly; weekly | NR | P(NR) |
| Popp et al., 2014 (ancillary trial of HORIZON study) | RCT/ Switzerland | 110/F(100)/ZOL: 76.5  (5); PLB: 77(5.2) | PM OP/ZOL: −2.6  (0.52); PLB: −2.7  (0.47)/ ZOL: 24.6  (3.6); PLB: 24.4  (3.7) | P(ZOL:41.8; PLB: 52.7) | NR | ZOL; PLB | 5mg/IV/annually |  | P(100) |
| Reid et al., 2018 | RCT/New Zealand | 2000/F(100)/ZOL: 71(5); PLB: 71(5.1) | PM Osteopenic/ZOL: −1.64(0.47); PLB: −1.63(0.47)/ZOL: 26.8(4.6); PLB: 26.9(4.7) | P for vertebral fractures (ZOL: 13.7; PLB: 12.6); P for non-vertebral fractures (ZOL: 23.7; PLB: 23.8) | NR | ZOL: PLB | 5mg/IV/(18m interval) | NR | NP |
| Sestak et al., 2014 (IBIS-II trial) | RCT/International | 303/F(100)/RIS: 60.46(5.8); PLB: 59.99(6.13) | PM  osteopenic women at increased risk of breast cancer/RIS: -1.64(0.57); PLB: -1.41(0.56)/RIS: 26.55(3.86);PLB: 27.06(5.12) | NP | P (anastrozole) | RIS; PLB | 35mg/oral/weekly | NR | NP |
| Shi et al., 2017 | RCT/China | 156/F(100)/ALN: 59.8 (4.7); PLB: 59.4 (4.5) | PM OP women/ALN: −1.91 (0.65); PLB: −1.83 (0.61)/ALN: 22.8 (3.2); PLB: 23 (3.2) | P(NR) | NP | ALN;PLB | 70mg/oral/weekly | NP | P(100) |
| Shin et al., 2017 | RCT/South Korea | 157/F(100)/IBN: 54.5(9.3); PLB: 55.1(8.6) | Osteopenic women/IBN: -1.53(0.83); PLB: -1.28(0.94)/IBN: 22.9(4.1); PLB: 23.8(3.5) | NR | P(prednisolone) | IBN;PLB | 150mg/oral/monthly | P(100) | NP |
| Tan et al., 2016 | RCT/China | 105/F(100)/ALN: 68(8.55); ZOL: 68.1(9.02) | PM OP/NR/ALN: 22(4.71); ZOL: 22(4.94) | NR | P(PTH) | ALN; ZOL | 70mg; 5mg/oral; IV/weekly; annually | NP | P(100) |
| Zhang et al., 2019 | RCT/China | 101/F(100)/ZOL: 64.6 (6.7); PLB: 63.98 (7.51) | PM OP women after undertaking PKP/NR/ZOL: 26.13 (1.81); PLB: 26.15 (2.23) | P(100) | NR | ZOL(plus calcitriol and calcium carbonate D3); PLB(calcitriol and calcium carbonate D3) | 5mg/IV/annually | NR | P(100) |
| Zhang et al., 2015 | RCT/China | 208/F(100)/ALN: 65.6 (8.0); PLB: 64.8 (7.4) | PM women/NR/ALN: 23.0 (3.5); PLB: 22.7 (2.9) | P(NR) | NR | ALN(plus vitamin D3); PLB(calcitriol 0.25 μg  Daily) | 70mg/oral/weekly | NR | P(NR) |
| Zhou et al., 2020 | RCT/China | 123/F(25.2)/ALN: 83.16 (3.09); PLB: 83.92 (2.85) | Osteopenic patients/NR/ALN: 23.95 (2.93); PLB: 24.2 (3.22) | NR | NR | ALN(plus 600 mg/d of calcium  carbonate, 0.5 μg/d of alfacalcidol); PLB (600 mg/d of calcium carbonate and 0.5 μg/d of alfacalcidol) | 70mg/oral/weekly | NP | NP |
| ***Note***. ADT: Androgen deprivation therapy; AI: Aromatase-inhibitors; ALN: Alendronate; BC: Breast-cancer; Ext.: Extension; GC: Glucocorticoids; IBN: Ibandronate; IV: Intravenously; NP: Not present; NR: Not reported; OP: Osteoporosis; OVCF: osteoporotic vertebral compression fracture; P: Present; PFT: Pivotal Fracture Trial; PKP: percutaneous Kyphoplasty; PLB: Placebo; PM: Post-menopausal; PTH: Parathyroid Hormone; PVP: percutaneous vertebroplasty; RA: Rheumatoid arthritis; RIS: Risedronate; ZOL: Zoledronate  ^a^Different colours indicate different pairwise comparisons (purple: ZOL vs PLB; orange: ALN vs PLB; green: IBN150mg vs PLB; and red: Risedronate versus PLB). No-coloured rows indicate trials with active treatment comparisons.  ^b^Total number of participants included in the analysis  ^c^Concomitant medication category provides information regarding co-medications other than generic calcium and vitamin supplements. | | | | | | | | | |

| **Table 10. Femoral neck BMD, number of fractures and deaths** | | | |
| --- | --- | --- | --- |
| **Study ID** | **Fractures** | **FNBMD** | **Mortality** |
| Black et al., 2015  Ext.: Black et al., 2012; Black et al., 2007 | Morphometric vertebral(n/N), OR(95%CI),p value  ZOL: 3/68  PLB: 5/69; OR = 0.58(0.13, 2.55), p=0.461  Clinical fractures(n/N), HR(95%CI),p value  ZOL: 10/95, HR=1.11(0.45, 2.73), p=0.821  PLB: 9/95 | Mean(%) difference (between-groups)  7 years:  (95%CI)  ZOL: 0.46(-0.75, 1.67)  PLB: Ref.  Mean % diff. 8 years (95%CI)  ZOL: 0.88(-0.53, 2.3)  PLB: Ref.  Mean % diff. 9 years (95%CI)  ZOL: 0.06(-0.41, 1.53)  PLB: Ref. | Mortality(n/N), HR(95%CI)  ZOL: 1/92, HR=0.2(0.02, 1.74),  PLB: 5/95 |
| Cesareo et al., 2015 | NR | Absolute change (%)  ALN: 0.017; (2.6)  PLB: 0.011; (−1.7) | NR |
| Cheung et al., 2020 | Non-vertebral (n/N)  ZOL: 1/39  PLB: 0/37 | NR | Mortality (n/N)  ZOL: 0/39  PLB: 0/37 |
| Cosman et al., 2016 | NR | % change, SE (12 months) *  ALN: -0.06(0.454)  PLB: -1.23(0.438) | NR |
| Eastell et al., 2011  Ext.:  Eastell et al., 2014 | NR | % change, SE (12 months) *  ALN: 2.61(0.45)  PLB: 0.141(0.43)  % change, SE (24 months) *  ALN: 2.847(0.39)  PLB: -0.543(0.46) | NR |
| Greenspan, Perrera et al., 2015 | Total number of fractures (n/N)  ZOL: 18/89  PLB: 15/92  Vertebral fractures (n/N)  ZOL: 6/89  PLB: 8/92 | % change, SE (12 months) *  ZOL: 2.31(0.79)  PLB: -1.28(0.65)  % change, SE (24 months) *  ZOL: 0.18(0.63)  PLB: -3(0.78) | Mortality(n/N)  ZOL: 14/89  PLB: 12/92 |
| Greenspan, Vujevich et al., 2015 | NR | % change, SE (12 months) *  RIS: -0.33(0.48)  PLB: -1.24(0.55)  % change, SE (24 months) *  RIS: 0.37(0.64)  PLB: -2.15(0.64) | Mortality(n/N)  RIS: 1/55  PLB: 0/54 |
| Grey et al., 2012  Ext.: Grey et al., 2014  Grey et al., 2017 | Fractures (12 months)  ZOL: 1/45 (tibia)  PLB: 2/45 (metatarsal)  Fractures(24 months)  ZOL: 1/43 (finger)  PLB: 1/43 (forearm)  Fractures(60 months)  ZOL: 3/41(toe; hand; forearm)  PLB: 2/34 (humerus; rib) | NR | NR |
| Hu et al., 2020 | Vertebral fractures (n/N)  ZOL: 2/121  PLB: 13/121 | NR | NR |
| Li et al., 2018 | NR | Absolute value (SD), Baseline  ALN: 0.55 (0.06)  PLB: 0.54 (0.06)  Absolute value (SD), 12 months  ALN: 0.65 (0.05)  PLB: 0.59 (0.07) | NR |
| Li et al., 2016 | Fractures (n/N)  ZOL: 0/30  PLB: 4/30 (two hip and two distal radius) | Absolute value (SD), Baseline  ZOL: 0.65 (0.13)  PLB: 0.69 (0.11)  Absolute value (SD), 12 months  ZOL: 0.76 (0.14)  PLB: 0.7 (0.10) | NR |
| Liang et al., 2017 | NR | NR | NR |
| Liu et al., 2019 | Intertrochanteric fractures (n/N)  ZOL: 21/353  PLB: 11/129 | NR | NR |
| Livi et al., 2019  (BONADIUV trial) | NR | NR | Mortality  IBN: 6/89  PLB: 2/82 |
| McClung et al., 2014 | NR | % change (SE)  ALN: 1.2(0.459)  PLB: -1.1(0.459) | Mortality (n/N)  ALN: 0/51  PLB: 1/50 |
| Nakamura et al., 2017 | Morphometric vertebral fractures  ZOL: 10/330  PLB: 29/327  Clinical vertebral fractures  ZOL: 6/330  PLB: 18/331  Non-vertebral fractures  ZOL: 20/330  PLB: 37/331  Hip fractures  ZOL: 2/330  PLB: 3/331 | % change 12 months, (SE)*  ZOL: 3.06(0.301)  PLB: 0.79(0.31)  % change 24 months, (SE)*  ZOL: 3.58(0.322)  PLB: -0.46(0.398) | Mortality (n/N)  ZOL:2/333  PLB: 3/332 |
| Paggiosi et al., 2014 (TRIO study) | Fractures (data provided by the authors)  ALN: 3 (2 wrist; 1 metatarsal)  IBN: 1/57(scaphoid)  RIS: 0 | % change 12 months, (SE)  ALN: 2.13 (0.485)  IBN: 3.12 (0.611)  RIS: 1.78 (0.595)  % change 24 months, (SE)  ALN: 3.97 (0.611)  IBN: 3 (0.513)  RIS: 1.91 (0.659) | NR |
| Popp et al., 2014 | Morphometric vertebral fractures(n/N)  ZOL: 1/55  PLB: 5/55  Non-vertebral fracture(n/N)  ZOL: 8/55  PLB: 7/55 | % change 12 months, (SE)*  ZOL: 2.38(0.61)  PLB: -0.05(0.6)  % change 24 months, (SE)*  ZOL: 2.73(0.92)  PLB: 0.6(0.84)  % change 36 months, (SE)*  ZOL: 2.76(0.897)  PLB: -0.98(0.852) | NR |
| Reid et al., 2018 | Vertebral fractures (n/N)  ZOL: 23/1000  PLB: 49/1000  Non-vertebral fractures (n/N)  ZOL: 101/1000  PLB: 148/1000  Hip fractures (n/N)  ZOL: 8/1000  PLB: 12/1000  Wrist/forearm fractures (n/N)  ZOL: 36/1000  PLB: 63/1000 | NR | Mortality(n/N)  ZOL: 27/1000  PLB: 41/1000 |
| Sestak et al., 2014  Ext.  Sestak et al., 2019 (IBIS-II study) | NR | NR | Mortality(n/N)  RIS: 2/253  PLB: 2/247 |
| Shi et al., 2017 | NR | % change 6months (SD)*  ALN: 1.474 (0.49)  PLB: -0.112(0.17)  % change 12 months (SD)*  ALN: 1.637(0.63)  PLB: -0.15(0.18) | NR |
| Shin et al., 2017 | Wrist fractures (n/N)  IBNor: 0/81  PLB: 1/76 | % change 6months (SD)  IBNor: 1.7, (5.3)  PLB: 0.4(5.3)  % change 12 months (SD)  IBNor: 1.7(5.4)  PLB: -1.2(7.5) | NR |
| Tan et al., 2016 | NR | % change 12months (SD)  ALN: 2.1(1.1)  ZOL: 7.7(1.5)  % change 24months (SD)  ALN: 4.2(1.8)  ZOL: 11.5(5.1)  % change 36months (SD)  ALN: 6.3(2.3)  ZOL: 13.5(6.8) | NR |
| Zhang et al., 2019 | Vertebral fractures (n/N)  ZOL: 0/50  PLB: 5/51 | NR | NR |
| Zhang et al., 2015 | Non-vertebral fractures (n/N)  ALN: 1/107  PLB: 2/108  Vertebral fractures (n/N)  ALN: 0  PLB: 2/108 | % change 6 months (95%CI)  ALN: 2.8 (1.2, 4.3)  PLB: 0.8 (-0.7, 2.2)  % change 12 months (95%CI)  ALN: 3.3 (1.9, 4.7)  PLB: 0.4 (-0.9, 1.8) | NR |
| Zhou et al., 2020 | Overall fragility fractures (n/N)  ALN: 5/62  PLB: 12/60 | NR | Mortality (n/N)  ALN: 0/62  PLB: 1/61 |
| ***Note.*** ALN: Alendronate; CI: Confidence interval; HR: Hazard ratio; IBN-or: Ibandronate 150mg; n: number of patients; N: overall sample; NR: Not reported; OR: Odds-ratio; PLB: Placebo; RIS: Risedronate; SD: Standard deviation; SE: Standard error; ZOL: Zoledronate  *Data extracted from graphs. | | | |
|  |  |  |  |

| **Table 11. Adverse events, serious adverse events and health-related quality of life (HRQoL)** | | | |
| --- | --- | --- | --- |
| **Study ID** | **AEs (overall & by type) (n/N)** | **SAEs (overall & by type)** | **HRQoL** |
| Black et al., 2015  Ext.: Black et al., 2007; Black et al., 2012 | Overall number of subjects with any AE  ZOL: 80/92  PLB: 80/95  AEs-specific (<3days after infusion, 1-60 months)  Pyrexia  ZOL: 2/92  PLB: 0/95  Myalgia  ZOL: 1/92  PLB: 2/95  Influenza-like illness  ZOL: 1/92  PLB: 0/95  Headache  ZOL: 1/92  PLB: 0/95  Atrial fibrillation  ZOL: 5/92  PLB: 1/95  Atrioventricular block first degree  ZOL:2/92  PLB: 0/95  Bundle branch block left  ZOL: 2/92  PLB:0/95  Bundle branch block right  ZOL: 2/92  PLB:0/95  Arrhythmia  ZOL: 1/92  PLB:1/95  Sinus bradycardia  ZOL: 1/92  PLB:0/95  Supraventricular extrasystoles  ZOL: 0/92  PLB:1/95  Tachycardia  ZOL: 0/92  PLB:1/95  Ventricular extrasystoles  ZOL: 0/92  PLB:1/95 | Overall number of subjects with any SAE  ZOL: 24/92  PLB: 28/95  SAEs-specific (<3days after infusion, 1-60 months)  Atrial fibrillation  ZOL: 1/92  PLB: 1/95  Arrhythmia  ZOL: 0/92  PLB: 1/95  Palpitations  ZOL: 0/92  PLB: 1/95 | NR |
| Cesareo et al., 2015 | NR | NR | NR |
| Cheung et al., 2020 | Overall number of subjects with any AE  ZOL: 27/38  PLB: 22/36  Flu-like symptoms (<7days)  ZOL: 17/38  PLB: 8/36  Anterior uveitis (<7days)  ZOL: 1/38  PLB: 0/36  Dermatitis(<7days)  ZOL: 0/38  PLB: 1/36  Tiredness(<7days)  ZOL: 11/38  PLB: 8/36  Hepatitis(<7days)  ZOL: 0/38  PLB:1/36  Leg swelling(<7days)  ZOL: 4/38  PLB:1/36  Cardiovascular(<7days)  ZOL: 3/38  PLB: 4/36  Prostate cancer progression(<7days)  ZOL: 1/38  PLB: 2/36  Autoimmune disease(<7days)  ZOL: 2/38  PLB: 1/36  Osteoporosis(<7days)  ZOL: 2/38  PLB: 0/36  Other malignancy(<7days)  ZOL: 0/38  PLB: 2/36 | Overall number of subjects with any SAE  ZOL: 11/38  PLB: 11/36 | NR |
| Cosman et al., 2016 | Overall number of subjects with any AE  ALN: 55/84  PLB: 65/88 | Overall number of subjects with any SAE  ALN: 1/84  PLB: 6/88 | NR |
| Eastell et al., 2014  Ext.:  Eastell et al., 2011 | Overall number of subjects with any AE  ALN: 49/57  PLB: 50/57  Abdominal pain upper abdominal pain  ALN: 8/57  PLB: 2/57  Constipation  ALN: 1/57  PLB: 2/57  Dyspepsia  ALN: 3/57  PLB: 6/57  Nasopharyngitis  ALN: 9/57  PLB: 9/57  Hypercholesterolemia  ALN: 2/57  PLB: 6/57  Arthralgia  ALN: 10/57  PLB: 6/57  Back pain  ALN: 7/57  PLB: 13/57  Osteoarthritis  ALN: 7/57  PLB: 2/57  Dizziness  ALN: 1/57  PLB: 4/57  Headache  ALN: 1/57  PLB: 7/57  Hypertension  ALN: 7/57  PLB: 6/57  Rash  ALN: 1/57  PLB: 0/57 | Overall number of subjects with any SAE  ALN: 6/57  PLB: 6/57 | NR |
| Greenspan, Perrera et al., 2015 | Overall number of subjects with any AE  ZOL: 87/89  PLB: 88/92  Cardiac disorders  ZOL: 28/89  PLB: 25/92  Atrial fibrillation  ZOL: 5/89  PLB: 5/92  ***<3 days after infusion***  Headache  ZOL: 14/89  PLB: 6/92  Pyrexia  ZOL: 7/89  PLB: 0  Fatigue  ZOL: 21/89  PLB: 14/92  Arthralgia  ZOL: 10/89  PLB: 6/92  Myalgia  ZOL: 7/89  PLB: 3/92  Influenza-like symptoms  ZOL: 6/89  PLB: 2/92 | Overall number of subjects with any SAE  ZOL: 60/89  PLB: 55/92 | NR |
| Greenspan, Vujevich et al., 2015 | Overall number of subjects with any AE  RIS: 52/55  PLB: 50/54  Musculoskeletal  RIS: 27/55  PLB: 33/54  Respiratory  RIS: 4/55  PLB: 10/54  Cardiovascular  RIS: 8/55  PLB: 11/54  Gastrointestinal  RIS: 4/55  PLB: 13/54  Breast related  RIS: 5/55  PLB: 4/54 | Overall number of subjects with any SAE  RIS: 10/55  PLB: 16/54 | NR |
| Grey et al., 2012  Ext.: Grey et al., 2014; Grey et al., 2017 | Iritis  ZOL: 1/45  PLB: 0/45  Fever  ZOL: 11/45  PLB: 2/45  General  ZOL: 32/45  PLB: 12/45  Musculoskeletal  ZOL: 27/45  PLB: 6/45  Gastrointestinal  ZOL: 13/45  PLB: 5/45  Any of the above  ZOL: 36/45  PLB: 16/45 | NR | NR |
| Hu et al., 2020 | Bone cement leakage  ZOL: 8/121  PLB: 10/121  Fever  ZOL: 21/121  PLB: 0/121  Flu-like symptoms  ZOL:17/121  PLB: 0/121  Myalgia  ZOL: 9/121  PLB: 0/121 | NR | NR |
| Li et al., 2018 | Gastrointestinal  ALN: 3/50  PLB: 0/50 | NR | NR |
| Li et al., 2016 | Overall number of subjects with any AE  ZOL: 6/30 (4 experienced fever and influenza and 2 emesis)  PLB: NR | NR | HRQoL (OQOLS scores, SD)  ZOL: 83.30 (9.4)  PLB: 78.26 (9.8) |
| Liang et al., 2017 | Headache  ZOL: 21/155  PLB: 2/95  Pyrexia  ZOL: 43/155  PLB: 3/95  Myalgia  ZOL: 34/155  PLB: 4/95  Arthralgia  ZOL: 29/155  PLB: 11/95  Back pain  ZOL: 24/155  PLB: 14/95 | NR | NR |
| Liu et al., 2019 | Overall number of subjects with any AE  ZOL: 149/353  PLB: NR  Gastrointestinal  ZOL: NR  PLB: 15/129 | NR | Quality of life per group is reported at 12 and 24 months (SF-36) |
| Livi et al., 2019 ( BONADIUV trial) | Overall number of subjects with any AE  IBN:36/72  PLB: 39/72  Cardiac events  IBN:4/72  PLB:1/72  Arthralgia/bone pain/myalgia  IBN:31/72  PLB:32/72  Dyspepsia  IBN:13/72  PLB:14/72  Flu-like symptoms  IBN:8/72  PLB:3/72  Hot flashes  IBN:5/72  PLB:3/72  Vaginal dryness  IBN:4/72  PLB: 0  Mood disorders  IBN:4/72  PLB:3/72  Dizziness  IBN:3/72  PLB:1/72  Insomnia  IBN:3/72  PLB:3/72  Diarrhoea  IBN:2/72  PLB:2/72  Weight gain  IBN:2/72  PLB:1/72  Breast pain  IBN:2/72  PLB:3/72  Headache  IBN:2/72  PLB:3/72  Thromboembolic event  IBN:1/72  PLB:1/72  Anaemia  IBN:1/72  PLB:1/72  Uterine polyps  IBN:1/72  PLB:1/72 | Overall number of subjects with any SAE  IBN:7/72  PLB: 7/72 | NR |
| McClung et al., 2014 | Overall number of subjects with any AE  ALN: 44/51  PLB: 45/50  Nasopharyngitis  ALN: 3/51  PLB: 7/50  Arthralgia  ALN: 5/51  PLB: 4/50  Pain in extremity  ALN: 2/51  PLB: 2/50  Back pain  ALN: 5/51  PLB: 3/50  Gastroenteritis  ALN: 2/51  PLB: 3/50  Headache  ALN: 4/51  PLB: 8/50  Cough  ALN: 4/51  PLB: 2/50  Constipation  ALN: 3/51  PLB: 2/50  Bronchitis  ALN: 1/51  PLB: 2/50  Urinary tract infection  ALN: 4/51  PLB:0/50  Fatigue  ALN: 2/51  PLB: 2/50  Musculoskeletal pain  ALN: 2/51  PLB: 2/50 | Overall number of subjects with any SAE  ALN: 4/51  PLB: 7/50 | NR |
| Nakamura et al., 2017 | Overall number of subjects with any AE  ZOL: 315/333  PLB: 306/332  Pyrexia  ZOL: 131/333  PLB: 11/332  Nasopharyngitis  ZOL: 116/333  PLB: 90/332  Arthralgia  ZOL: 54/333  PLB: 24/332  Osteoarthritis  ZOL: 44/333  PLB: 39/332  Myalgia  ZOL: 36/333  PLB: 6/332  Eczema  ZOL: 31/333  PLB: 24/332  Constipation  ZOL: 30/333  PLB: 29/332  Malaise  ZOL: 30/333  PLB: 10/332  Fall  ZOL: 29/333  PLB: 29/332  Periarthritis  ZOL: 29/333  PLB: 21/332  Contusion  ZOL: 28/333  PLB: 43/332  Headache  ZOL: 25/333  PLB: 13/332  Blood calcium decreased  ZOL: 24/333  PLB: 2/332  Influenza-like illness  ZOL: 23/333  PLB: 0  Back pain  ZOL: 21/333  PLB: 18/332  Upper respiratory tract inflammation  ZOL: 21/333  PLB: 18/332  Protein urine present  ZOL: 21/333  PLB: 4/332  Dermatitis contact  ZOL: 18/333  PLB: 16/332  Spinal osteoarthritis  ZOL: 17/333  PLB: 14/332 | Overall number of subjects with any SAE  ZOL: 58/333  PLB: 44/332 | NR |
| Paggiosi et al., 2014 | Overall number of subjects with any AE  ALN: 2/57  IBN: 7/57  RIS: 1/58  Atrial fibrillation  ALN: 2/57  IBN: 1/57  RIS: 1/58  Conjunctivitis  ALN: 0/57  IBN: 2/57  RIS: 2/58  Dyspepsia  ALN: 7/57  IBN: 4/57  RIS: 5/58 | Overall number of subjects with any SAE  ALN: 2/57  IBN: 7/57  RIS: 8/58 | NR |
| Popp et al., 2014 | Overall number of subjects with any AE  ZOL: 55/55  PLB: 54/55 | Overall number of subjects with any SAE  ZOL: 27/55  PLB: 28/55 | NR |
| Reid et al., 2018 | Myocardial infarction  ZOL: 24/1000  PLB: 39/1000  Coronary-artery revascularization  ZOL: 21/1000  PLB: 30/1000  Stroke  ZOL: 17/1000  PLB: 20/1000  Composite of vascular events  ZOL: 53/1000  PLB: 69/1000  Transient ischemic attack  ZOL: 23/1000  PLB: 14/1000  Cancer  ZOL: 84/1000  PLB: 121/1000  Atrial fibrillation  ZOL: 54/1000  PLB: 55/1000 | Overall number of subjects with any SAE  ZOL: 400/1000  PLB: 443/1000 | NR |
| Sestak et al., 2014, (IBIS-II study( | Overall number of subjects with any AE  RIS: 86/145  PLB: 113/158  Arthralgia  RIS: 59/145  PLB: 73/158  Hot flush  RIS: 10/145  PLB: 12/158  Alopecia  RIS: 6/145  PLB: 5/158  Abdominal pain  RIS: 3/145  PLB: 6/158  Essential hypertension  RIS: 1/145  PLB: 1/158  Cataract  RIS: 1/145  PLB: 2/158  Arthritis  RIS: 1/145  PLB: 2/158  Amnesia  RIS: 1/145  PLB: 1/158  Anxiety  RIS: 0/145  PLB: 1/158  Back pain  RIS: 1/145  PLB: 3/158  Abdominal distension  RIS: 1/145  PLB: 2/158  Constipation  RIS: 0/145  PLB: 1/158  Cystitis  RIS: 0/145  PLB: 2/158  Dyspepsia  RIS: 0/145  PLB: 1/158  Emotional disorder  RIS: 0/145  PLB: 1/158  Gynaecological events  RIS: 2/145  PLB: 0/158 | NR | NR |
| Shi et al., 2017 | NR | NR | NR |
| Shin et al., 2017 | Overall number of subjects with any AE  IBN: 58/81  PLB: 52/76  Gastrointestinal disorders  IBN: 27/81  PLB: 19/76  Dyspepsia  IBN: 6/81  PLB: 6/76  Nausea  IBN: 6/81  PLB: 3/76  Abdominal pain, upper  IBN: 4/81  PLB: 2/76  Constipation  IBN: 4/81  PLB: 2/76  Diarrhoea  IBN: 5/81  PLB: 1/76  Gastritis  IBN: 4/81  PLB: 1/76  Vomiting  IBN: 1/81  PLB: 1/76  Musculoskeletal & connective tissue disorders  IBN: 20/81  PLB: 21/76  Arthralgia  IBN: 6/81  PLB: 5/76  Myalgia  IBN: 4/81  PLB: 3/76  Back pain  IBN: 1/81  PLB: 5/76  Pain in extremity  IBN: 3/81  PLB: 3/76  Infections & infestations  IBN: 17/81  PLB: 15/76  Nasopharyngitis  IBN: 8/81  PLB: 6/76  Upper respiratory tract infection  IBN: 1/81  PLB: 3/76  Tinea pedis  IBN: 1/81  PLB: 2/76  General disorders & administration site conditions  IBN: 10/81  PLB: 7/76  Fatigue  IBN: 4/81  PLB: 2/76  Face oedema  IBN: 2/81  PLB: 2/76  Respiratory, thoracic & mediastinal disorder  IBN: 8/81  PLB: 8/76  Nervous system disorders  IBN: 6/81  PLB: 8/76  Skin & subcutaneous tissue disorders  IBN: 6/81  PLB: 8/76  Metabolism & nutrition disorders  IBN: 5/81  PLB: 9/76 | Overall number of subjects with any SAE  IBN: 5/81  PLB: 15/76  Musculoskeletal & connective tissue disorder  IBN: 3/81  PLB: 4/76  Spinal column stenosis  IBN: 2/81  PLB: 1/76  Arthralgia  IBN: 0/81  PLB: 2/76  Back pain  IBN: 0/81  PLB: 1/76  Neck pain  IBN: 0/81  PLB: 1/76  Other  IBN: 1/81  PLB: 0/76  Injury ,poisoning & procedural complications  IBN: 0/81  PLB: 5/76  Contusion  IBN: 0/81  PLB: 2/76  Ligament sprain  IBN: 0/81  PLB: 1/76  Joint injury  IBN: 0/81  PLB: 1/76  Wrist fracture  IBN: 0/81  PLB: 1/76  Multiple fractures  IBN: 0/81  PLB: 1/76  Other  IBN: 0/81  PLB: 1/76  Infections & infestations  IBN: 1/81  PLB: 2/76  Gastrointestinal disorders  IBN: 1/81  PLB: 1/76  Nervous system disorders  IBN: 1/81  PLB: 1/76 | NR |
| Tan et al., 2016 | Pyrexia/flu-like symptoms  ALN: NR  ZOL: 21/52  Gastrointestinal events (12-month, 24-month, 36-month)  ALN: 14/53, 23/53, 29/53  ZOL: NR, NR, NR | NR | NR |
| Zhang et al., 2019 | Overall number of subjects with any AE  ZOL: 18/50  PLB: 0/51  Fever  ZOL: 10/50  PLB: 0/51  Flu-like symptoms  ZOL: 3/50  PLB: 0/51  Arthralgia  ZOL: 3/50  PLB: 0/51  Myalgia  ZOL: 2/50  PLB: 0/51 | NR | NR |
| Zhang et al., 2015 | Overall number of subjects with any AE  ALN: 64/107  PLB: 74/108  Nasopharyngitis  ALN: 10/107  PLB: 12/108  Abdominal pain, upper  ALN: 9/107  PLB: 3/108  Urine calcium increased  ALN: 8/107  PLB: 12/108  Upper respiratory tract infection  ALN: 7/107  PLB: 6/108  Arthralgia  ALN: 6/107  PLB: 5/108  Diarrhoea  ALN: 4/107  PLB: 6/108  Vitamin D deficiency  ALN: 0  PLB: 6/108  One or more incidents of hypercalcemia  ALN: 0  PLB: 1/108  One or more incidents of hypercalciuria  ALN: 9/107  PLB: 15/108 | NR | NR |
| Zhou et al., 2020 | Upper abdominal discomfort  ALN: 5/62  PLB: 0/61  Tooth extraction  ALN: 2/62  PLB: 0/61  Severe pneumonia  ALN: 0  PLB: 1/61  Cardiac cancer  ALN: 1/62  PLB: 1/61  Severe constipation  ALN: 0  PLB: 1/61  Hypercalcemia  ALN: 2/62  PLB: 2/61  Hypercalciuria  ALN: 5/62  PLB: 5/61 | NR | NR |
| ***Note.*** AE: Adverse events; ALN: Alendronate; HRQoL: Health-related quality of life; IBN: Ibandronate 150mg; NR: Not reported; PLB: Placebo; RIS: Risedronate; SAEs: Serious adverse events; ZOL: Zoledronate | | | |

| **Appendix 3: Network plots**  **Table 12. Network plots for all outcomes included in the analyses** | |
| --- | --- |
|  | |
| **Outcomes** | **Network plots*** |
| a) Vertebral fractures | 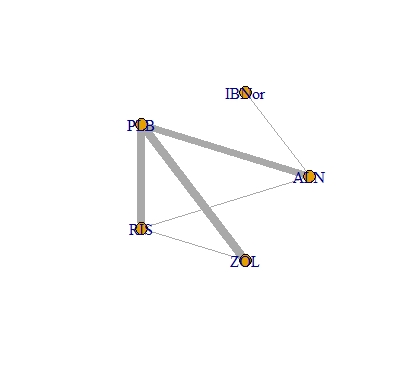 |
| b) Non-vertebral fractures | 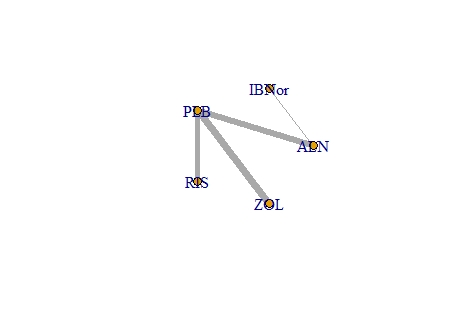 |
| c) Hip fractures | 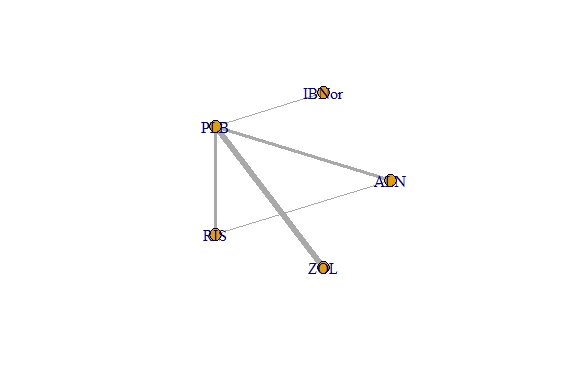 |
| d) Wrist fractures | 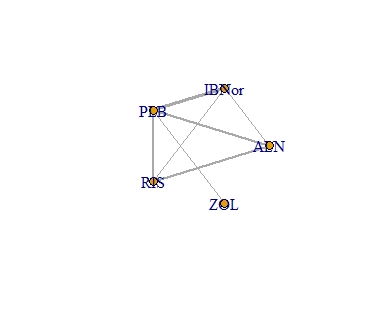 |
| e) % change in BMD – femoral neck | 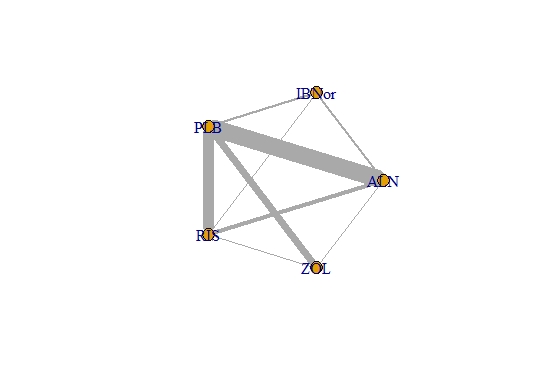 |
| f) Mortality | 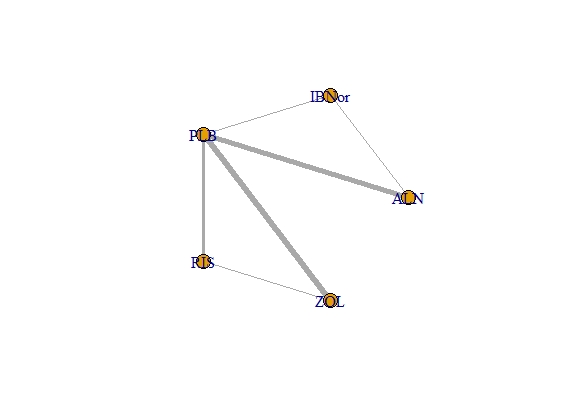 |
| g) Adverse events | 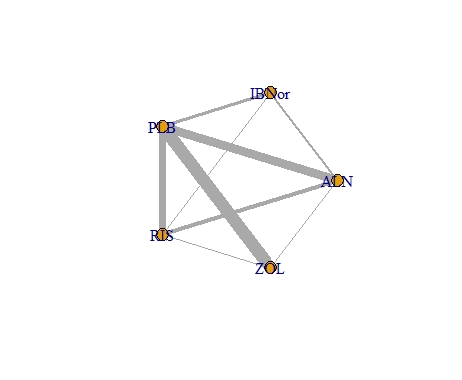 |
| h) Arthralgia | 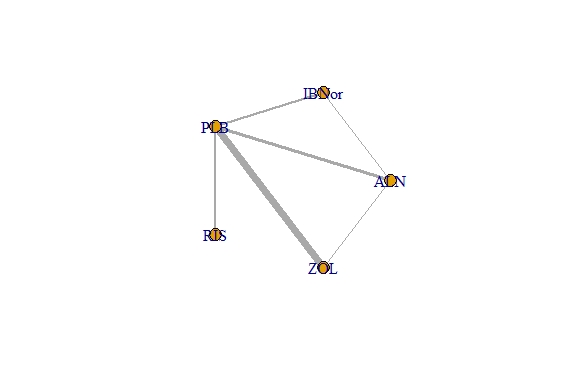 |
| i) Back pain | 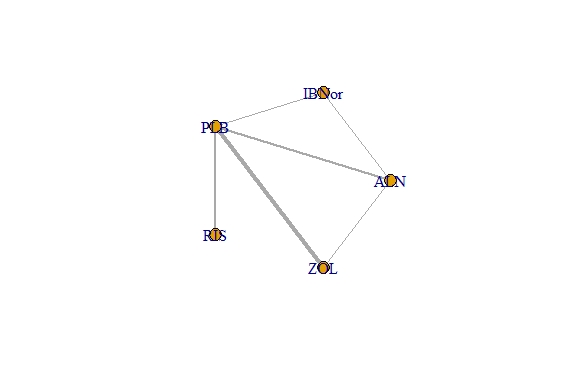 |
| j) Dyspepsia | 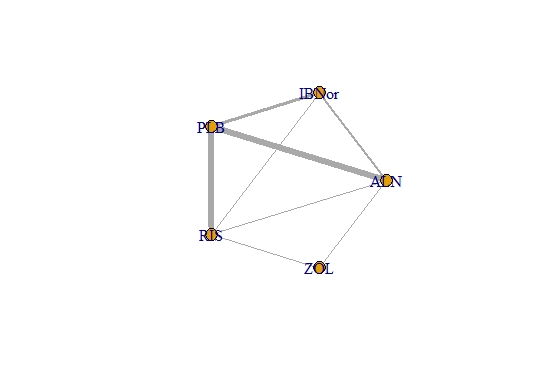 |
| k) Gastrointestinal | 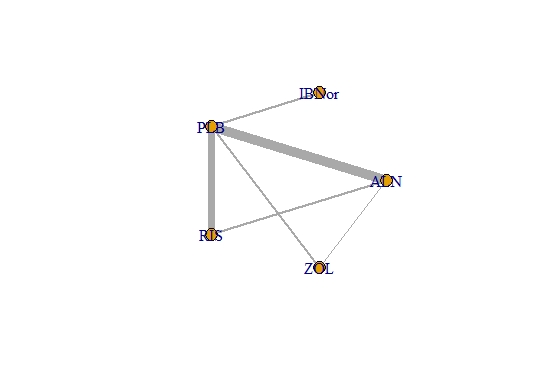 |
| l) Headache | 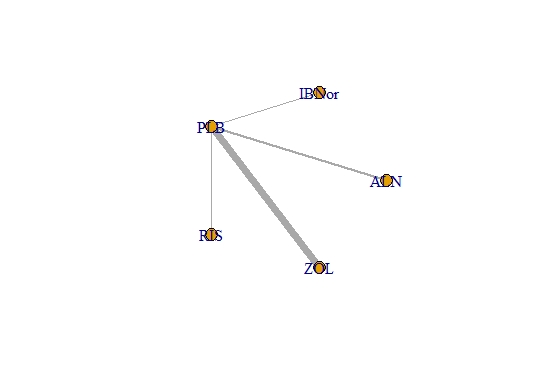 |
| m) Influenza-like symptoms | 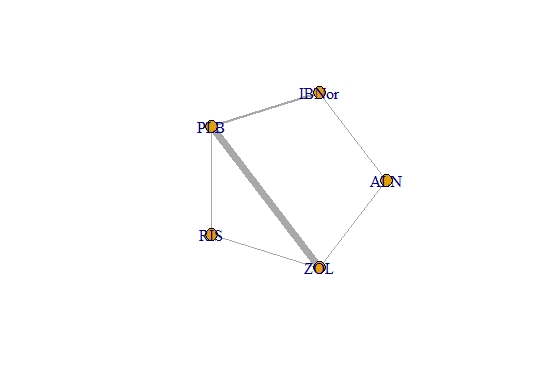 |
| n) Myalgia | 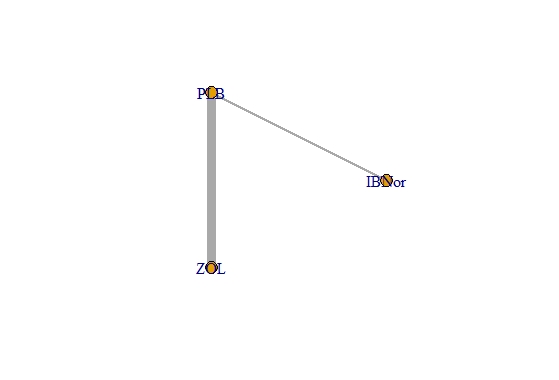 |
| o) Nasopharyngitis | 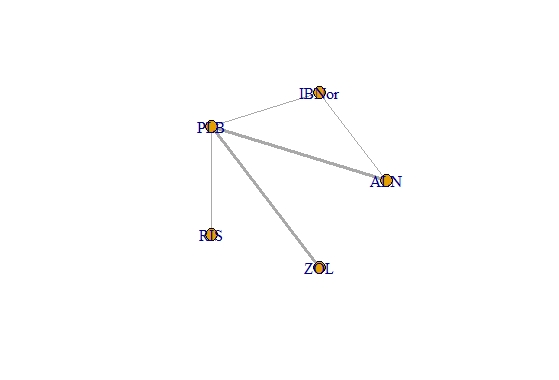 |
| p) Pyrexia | 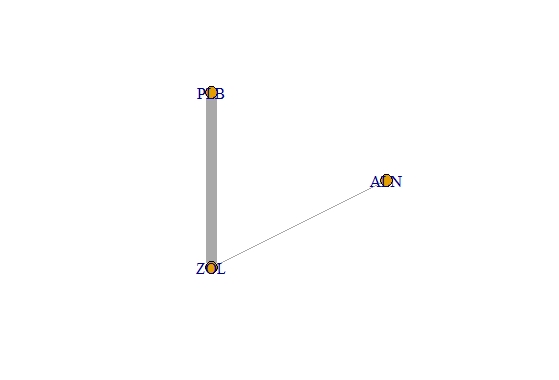 |
| ***Note***. ALN = Alendronate; IBN-or = Ibandronate 150mg; PLB = Placebo; RIS = Risedronate; ZOL = Zoledronate  * Treatment nodes indicate the study treatments, whereas edges’ thickness indicates the number of studies supporting each comparison. Proportionate sizing of nodes was not preferred, given the large divergences in the numbers of patients and studies across interventions. The only multi-arm trial (3-arm) included in the analysis provided data for BMD at femoral neck, wrist fractures, and adverse events. | |

| **Appendix 4. Treatment ranking probabilities** | |
| --- | --- |
| **Table 13a. Outcome: percentage change in femoral neck BMD** | |
| ZOL |  |
| ALN |  |
| IBNor |  |
| RIS |  |
| PLB |  |
| Femoral neck BMD: standard random-effects model. Probability of treatment rankings. Note that the most efficacious = 1 and the least efficacious = 5. Mean ranks: Zoledronate (ZOL) = 1.041; Alendronate (ALN) = 2.428, Ibandronate 150mg (IBNor) = 2.814, Risedronate (RIS) = 2.428, Placebo (PLB) = 5 | |

| **Table 13b: Outcome: Percentage change in femoral neck BMD** | |
| --- | --- |
| SUCRA | 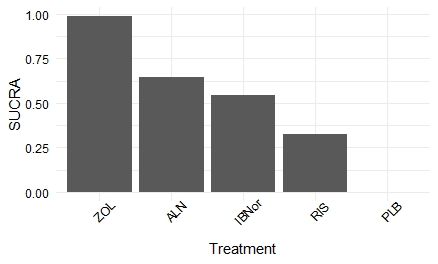 |
| Treatment ranking probabilities | 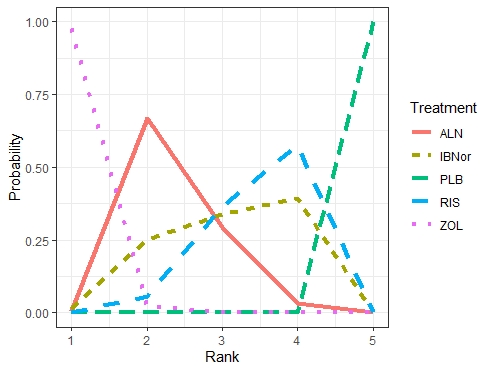 |
| ***Note.*** Femoral neck BMD: standard random-effects model. Treatment probabilities and SUCRA scores. Note that higher SUCRA values indicate more effective treatments. Zoledronate (ZOL): .99; Alendronate(ALN): .64; Ibandronate 150mg/oral(IBNor): .54; Risedronate(RIS): .32; Placebo(PLB): 0 | |

| **Table 14. Outcome: Vertebral fractures** | |
| --- | --- |
| ZOL |  |
| ALN |  |
| IBNor |  |
| RIS |  |
| PLB |  |
| Vertebral fractures: class-effects model. Probability of treatment rankings. Note that the most efficacious = 1 and the least efficacious = 5. Mean ranks: Zoledronate (ZOL) = 1.622; Alendronate (ALN) = 2.516, Ibandronate 150 mg/oral (IBNor) = 2.525, Risedronate (RIS) =3.357, Placebo (PLB) = 4.98 | |

| **Table 15. Outcome: Non-vertebral fractures** | |
| --- | --- |
| RIS |  |
| ZOL |  |
| IBNor |  |
| ALN |  |
| PLB |  |
| Non-Vertebral fractures: class-effects model. Probability of treatment rankings. Note that the most efficacious = 1 and the least efficacious = 5. Mean ranks: Risedronate (RIS) = 1.965; Zoledronate (ZOL) =2.197; Ibandronate 150 mg (IBNor) =2.804; Alendronate (ALN) =3.123, Placebo (PLB) =4.911 | |

| **Table 16. Outcome: Hip fractures** | |
| --- | --- |
| ZOL |  |
| ALN |  |
| IBNoral |  |
| RIS |  |
| PLB |  |
| Hip fractures: class-effects model. Probability of treatment rankings. Note that the most efficacious = 1 and the least efficacious = 5. Mean ranks: Zoledronate (ZOL) = 2.256; Alendronate (ALN) = 2.262; Ibandronate 150 mg (IBNor) = 2.711; Risedronate (RIS) = 2.92; Placebo (PLB) = 4.85 | |

| **Table 17. Outcome: Wrist fractures** | |
| --- | --- |
| ZOL |  |
| IBN-or |  |
| RIS |  |
| ALN |  |
| PLB |  |
| Wrist fractures: class-effects model. Probability of treatment rankings. Note that the most efficacious = 1 and the least efficacious = 5. Mean ranks: ; Zoledronate (ZOL) = 2.062; Ibandronate 150 mg (IBNor) = 2.422; Risedronate (RIS) = 2.533; Alendronate (ALN) = 3.374 ; Placebo (PLB) = 4.61 | |

| **Table 18. Outcome: Mortality** | |
| --- | --- |
| RIS |  |
| ZOL |  |
| IBNor |  |
| ALN |  |
| PLB |  |
| Mortality: class-effects model. Probability of treatment rankings. Note that the most efficacious = 1 and the least efficacious = 5. Mean ranks: Risedronate (RIS) = 2.241; Zoledronate (ZOL) = 2.565; Ibandronate 150 mg (IBNor) = 2.909 ; Alendronate (ALN) = 3.069 ; Placebo (PLB) = 4.216 | |

**Appendix 5. Analysis of hip & wrist fractures, analysis of secondary outcomes, sensitivity analyses and meta-regressions**

**Table 19.** **Analysis of secondary outcomes.** **Posterior median hazard ratios (95% CrI) for the treatment effects of bisphosphonates relative to placebo, heterogeneity parameter *σ* and model fit statistics**

|  | **Mortality*** | **Hip fractures*** | **Adverse events (overall)** |  | **Wrist fractures*** |
| --- | --- | --- | --- | --- | --- |
| *PLB - RIS* | .8(.36, 1.28) | .67(.5, .97) | 1.03(.8, 1.31) | *PLB - RIS* | .64(.34, 1.1) |
| *PLB - ALN* | .89(.62, 1.31) | .6166(.4, .86) | 1.004(.79, 1.26) | *PLB - ALN* | .79(.44, 1.27) |
| *PLB - ZOL* | .85(.65, 1.09) | .6162(.47, .79) | 1.52(1.19, 1.96) | *PLB - IBNor* | .61(.17, 1.42) |
| *PLB – IBNor* | .87(.47, 1.79) | .64(.27, 2.56) | 1.11(.77, 1.59) | *PLB - ZOL* | .54(.04, 1.36) |
| *σ* | .19 | .1 | .38 | *σ* | .29 |
| *D_res_* | 32.07 | 22.22 | 91.23 | *D_res_* | 21.83 |
| *pD* | 21.65 | 16.64 | 69.86 | *pD* | 13.62 |
| *DIC* | 175 | 144.8 | 539.8 | *DIC* | 95.26 |
| *Data points* | 34 | 28 | 77 | *Data points* | 21 |
| ***Note.*** ALN = Alendronate; CrI: Credible intervals; D_res_: total residual deviance; DIC: deviance information criterion; IBNor: Ibandronate 150mg oral; pD : effective number of parameters; PLB: Placebo; RIS: Risedronate; ZOL: Zoledronate; σ: between-study standard deviation  *Informative, half-normal prior distributions were used in order to ensure numerical stability, taking into account empirical plausibility. | | | | | |

| **Table 20. Additional analysis on specific adverse-events outcomes. Posterior median hazard ratios (95% CrI) for the treatment effects of bisphosphonates relative to placebo, heterogeneity parameter σ and model fit statistics** | | | | | | | | | | | |
| --- | --- | --- | --- | --- | --- | --- | --- | --- | --- | --- | --- |
|  | **Arthralgia** | **Back pain** | **Dyspepsia** | **Gastrointestinal** | **Headache*^,^**** | **Influenza-like symptoms*** | **Nasopharyngitis** |  | **Myalgia*^,^**** |  | **Pyrexia** |
| *PLB - RIS* | .93(.42, 1.97) | 1.22(.38, 4.23) | .9(.52, 1.5) | .93(.58, 1.42) | 3.6(.91, 24.49) | 1.09(.44, 2.93) | 1.06(.48, 2.11) | *PLB - ZOL* | 5.21(4.35, 6.3) | PLB - ALN | 1.96(.21, 9.97) |
| *PLB - ALN* | .96(.52, 1.88) | .41(.19, 1.35) | 1.44(.85, 2.6) | 1.01(.67, 1.5) | .36(.12, .89) | .86(.35, 2.49) | .92(.55, 1.44) | *PLB - IBNor* | 1.93(.67, 5.53) | PLB - ZOL | 9.37(7.11, 15.56) |
| *PLB - ZOL* | 1.95(1.17, 3.01) | 1.33(.66, 2.42) | .71(.22, 1.66) | 1.26(.72, 2.99) | 2.76(2.32, 3.29) | 6.05(3.7, 10.86) | 1.23(.83, 1.78) | *NA* | - | NA | - |
| *PLB – IBNor* | 1.15(.56, 2.38) | .5(.17, 1.68) | 1.11(.58, 2.06) | 1.13(.6, 2.64) | .72(.13, 3.02) | 1.56(.65, 4.58) | 1.08(.6, 1.79) | *NA* | - | NA | - |
| *σ* | .51 | .52 | .58 | .68 | - | .37 | .19 | *σ* | - | *σ* | .29 |
| *Dres* | 31.98 | 24.13 | 41.86 | 49.77 | 25.46 | 35.93 | 18.76 | *D_res_* | 24.69 | *D_res_* | 27.27 |
| *pD* | 26.98 | 19 | 32.39 | 43.91 | 14.2 | 18.22 | 13.27 | *pD* | 12.75 | *pD* | 17.27 |
| *DIC* | 199 | 137.1 | 240 | 346.8 | 130.1 | 140.8 | 112.1 | *DIC* | 124.9 | *DIC* | 142.9 |
| *Data points* | 32 | 22 | 39 | 48 | 22 | 24 | 18 | *Data points* | 22 | *Data points* | 24 |
| ***Note.*** ALN = Alendronate; CrI: Credible intervals; D_res_: total residual deviance; DIC: deviance information criterion; IBNor: Ibandronate 150mg oral; pD : effective number of parameters; RIS: Risedronate; ZOL: Zoledronate; σ: between-study standard deviation  *Informative, half-normal priors were used in order to ensure numerical stability.  **Estimates from the fixed-effect models were chosen to be reported for headache and myalgia symptoms. | | | | | | | | | | | |

**Synthesis of results on the secondary outcomes**

Outcome 1: Mortality

Data were available from 17, 2-arm, RCTs. The network provided six direct treatment comparisons (Appendix 3). Six treatment comparisons were assessed for consistency. The model had a good fit with the data with a total residual deviance of 32.07 (data points: 34; DIC=175). Six contrasts were checked for inconsistency with none of them showing significant evidence of inconsistency, as assessed using Bayesian p-values (*p*>0.1). The between-study SD was estimated to be 0.19 (95%CrI: 0.02, 0.35), implying mild heterogeneity in treatment effects between RCTs. The between-treatment SD was estimated to be 0.31 (95%CrI: 0.008, 1.29), which is indicative of mild-to-moderate heterogeneity in treatment effects between treatments but with considerable uncertainty. All treatments were associated with beneficial treatment effects relative to placebo, although the treatment effects were not statistically significant (*p*> 0.05). Risedronate was associated with the greatest effect, with HR = 0.8 (95%CrI: 0.36, 1.28).

Outcome 2: Overall adverse events (assessed as the total number of subjects experiencing at least an adverse event)

Data were available from 38 RCTs from which one study was a 3-arm trial. After removing the only 3-arm trial, the network provided eight direct treatment comparisons (plot g; Appendix 3). The model had a poor fit with the data with a total residual deviance of 91.23 (data points: 77; DIC=539.8); as such, a formal assessment of inconsistency was not performed. The between-study SD was estimated to be 0.38 (95%CrI: 0.26, 0.54), implying moderate heterogeneity in treatment effects between RCTs. The between-treatment SD was estimated to be 0.45 (95%CrI: 0.07, 1.75), which is indicative of moderate-to-high heterogeneity in treatment effects between treatments with substantial uncertainty. All treatments were associated with detrimental effects relative to placebo with the effects of zoledronate being significantly worse compared to placebo HR = 1.52 (95%CrI: 1.19, 1.96, *p*<.05).

Outcome 3: Arthralgia

Data were available from 16, 2-arm, trials. The model had a good fit with the data with a total residual deviance of 31.98 (data points: 32; DIC=199). Two connected networks were checked for inconsistency with evidence for inconsistency being evident in the relationship between alendronic acid and zoledronic acid (*p*<0.05). However, this direct relationship was informed by one small study. The between-study SD was estimated to be 0.51 (95%CrI: 0.23, 0.91), implying moderate-to-high heterogeneity in treatment effects between RCTs while the between-treatment SD was estimated to be 0.82 (95%CrI: 0.07, 3.23) implying high heterogeneity in treatment effects between treatments. All treatments were associated with detrimental effects relative to placebo with the effects of zoledronate being significantly worse compared to placebo HR = 1.95 (95%CrI: 1.17, 3.01, *p*<.05).

Outcome 4: Back pain symptoms

Data were available from 11, 2-arm, trials. The model had a relatively good fit with the data with a total residual deviance of 24.13 (data points: 22; DIC=137.1). Two network loops were tested for inconsistency with inconsistency being present in the relationship between alendronic acid and placebo (*p*<0.05); however, this relationship was informed by two small trials. The between-study SD was estimated to be 0.52 (95%CrI: 0.02, 1.41), implying moderate-to-high heterogeneity in treatment effects between RCTs while the between-treatment SD was estimated to be 1.3 (95%CrI: 0.11, 4.92) implying substantial heterogeneity in treatment effects between treatments. The treatment effects were not significantly different compared to placebo in any case (*p*>0.05). Risedronate and zoledronate were associated with the most detrimental effects compared to placebo.

Outcome 5: Dyspepsia

Data were available from 11 trials with one of them being a 3-arm trial. The model had a relatively good fit with the data with a total residual deviance of 41.86 (data points: 39; DIC=240). Three contrasts were checked for inconsistency with none of them showing significant evidence of inconsistency, as assessed using Bayesian *p*-values (*p*>0.05). The between-study SD was estimated to be 0.58 (95%CrI: 0.25, 1.05), implying high heterogeneity in treatment effects between RCTs. The between-treatment SD was estimated to be 0.79 (95%CrI: 0.004, 3.28), which is indicative of substantial heterogeneity in treatment effects between treatments. None of the treatment effects were statistically significant compared to placebo (*p*>0.05). Alendronate was found to have the most detrimental effects compared to placebo HR = 1.44 (95%CrI: .85, 2.06).

Outcome 6: Gastrointestinal symptoms

Data were available from 24, 2-arm trials. The model had a relatively good fit with the data with a total residual deviance of 49.77 (data points: 48; DIC=346.8). Five contrasts were checked for inconsistency. Evidence of inconsistency was present in the network loop of alendronate with zoledronate and placebo (*p*<.05), and more specifically in the relationships between alendronate versus zoledronate and zoledronate versus placebo. The former relationship was informed by one small trial while the latter was informed by two small trials. The between-study SD was estimated to be 0.68 (95%CrI: 0.46, 0.99), implying high heterogeneity in treatment effects between RCTs with reasonable uncertainty. The between-treatment SD was estimated to be 0.51 (95%CrI: 0.01, 2.27), which is indicative of moderate-to-high heterogeneity in treatment effects between treatments. None of the treatment effects were statistically significant different compared to placebo (*p*>0.05). Zoledronate was found to have the most detrimental effects compared to placebo HR = 1.26 (95%CrI: 0.72, 2.99).

Outcome 7: Headache symptoms

Data were available from 11, 2-arm, trials. The model had a relatively good fit with the data with a total residual deviance of 25.46 (data points: 22; DIC=130.1). Alendronate and zoledronate were found to be significantly different compared to placebo (*p*<0.05). Zoledronate was found to have the most detrimental effects compared to placebo HR = 2.76 (95%CrI: 2.32, 3.29, *p*<.05), while alendronate was to found to have beneficial effects compared to placebo HR = .36 (95%CrI: .11, .98, *p*<.05).

Outcome 8: Influenza-like symptoms

Data were available from 13, 2-arm, studies. The model had a poor fit with the data with a total residual deviance of 35.93 (data points: 24; DIC=140.8). Zoledronate was found to have the most detrimental effects compared to placebo HR = 6.05 (95%CrI: 3.7, 10.86, *p*<.05).

Outcome 9: Nasopharyngitis symptoms

Data were available from nine, 2-arm studies. None of the treatment-effects were significantly different compared to placebo (*p*>.05) with zoledronate being associated with the most detrimental effects compared to placebo HR = 1.23 (95%CrI: .83, 1.78).

Outcome 10: Myalgia symptoms

Data were available from 11, 2-arm, studies. The model had a relatively good fit with the data with a total residual deviance of 24.69 (data points: 22; DIC=124.9). Zoledronate was found to have the most detrimental effects compared to placebo HR = 1.93 (95%CrI: 0.67, 5.53, *p*<.05).

Outcome 11: Pyrexia symptoms

A NMA was used to compare the effects of zoledronate and alendronate versus placebo. Data were available from 12, 2-arm, studies. The model had a moderate fit with the data with a total residual deviance of 27.27 (data points: 24; DIC=142.9). Zoledronate was found to have the most detrimental effects compared to placebo HR = 9.37 (95%CrI: 7.11, 15.56, *p*<.05).

Outcome 12: Health-Related Quality of Life (HRQoL)

Two trials provided data about participants’ HRQoL both comparing ZOL versus PLB. In the first study^33^, participants’ quality of life was assessed at 12 months by using the Osteoporosis Quality of Life Scale (OQOLS). Participants in the ZOL group showed a statistically significant increase in OQOLS scores compared to participants in the PLB group (83.3 ± 9.4 vs 78.26 ± 9.8, p = 0.04). In the second study^35^, participants’ quality of life was assessed at 12 and 24 months by using the SF-36, targeting eight domains. Participants in the ZOL group showed statistically significant higher scores in the body pain, vitality, and mental health scores at 24 months compared to participants in the PLB group.

**Table 21. Sensitivity analysis on the main outcomes (% change in BMD of femoral neck, vertebral and non-vertebral fractures). Posterior mean differences (95%CrI) and posterior median hazard ratios (95%CrI) are reported for percentage changes in BMD of femoral neck and fracture outcomes respectively.**

|  | **Sensitivity 1^a^**  (outcome: % change FN BMD) | **Sensitivity 2**^b^  (outcome: % change FN BMD) | **Sensitivity (post-hoc)**  (outcome: % change FN BMD) |  | **Sensitivity^c^**  (outcome: vertebral fractures) | **Sensitivity^c^**  (outcome: non-vertebral fractures) |
| --- | --- | --- | --- | --- | --- | --- |
| *PLB - ALN* | 2.99(2.5, 3.48) | 2.98(2.34, 3.61) | 2.99(2.58, 3.41) | *PLB - RIS* | .50(.36, .67) | .66(.51, .82) |
| *PLB - RIS* | 2.35(1.78, 2.93) | 2.45(1.68, 3.19) | 2.24(1.72, 2.75) | *PLB - ALN* | .43(.26, .67) | .64(.42, .84) |
| *PLB - ZOL* | 3.69(2.91, 4.45) | 4.51(3.41, 5.68) | 3.49(2.78, 4.18) | *PLB - ZOL* | .41(.3, .55) | .69(.58, .79) |
| *PLB - IBNor* | 2.64(1.42, 3.87) | 2.8(1.53, 4.07) | 2.75(1.82, 3.68) | *PLB - IBNor* | .44(.18, .96) | .67(.39, 1.1) |
| *σ* | .75 | 1.07 | .71 | *σ* | .23 | .08 |
| *Dres* | 61.49 | 70.89 | 80.27 | *D_res_* | 43.47 | 23.96 |
| *pD* | 55.19 | 59.6 | 69.2 | *pD* | 28.98 | 19.4 |
| *DIC* | 100.3 | 141 | 154 | *DIC* | 239.8 | 183.6 |
| *Data points* | 66 | 73 | 87 | *Data points* | 44 | 32 |
| ***Note.*** ALN = Alendronate; BMD: Bone mineral density; CrI: Credible intervals; D_res_: total residual deviance; DIC: deviance information criterion; FN: Femoral neck; IBNor: Ibandronate 150mg oral; pD : effective number of parameters; RIS: Risedronate; ZOL: Zoledronate; σ: between-study standard deviation.  ^a^Studies with an overall high-risk of bias and the only independent ancillary sub-study were excluded in the sensitivity analysis of FN BMD.  ^b^Studies whose data were extracted from graphs were excluded from the analysis.  ^c^Studies with an overall high-risk of bias, studies in which patients were switched to different treatment doses, and the only independent sub-study were excluded in the sensitivity analysis of vertebral and non-vertebral fractures. | | | | | | |

**Table 22: Additional analysis on the BMD femoral neck at 12 months and 24-36 months. Mean differences with 95%CrI, heterogeneity parameters and model-fit statistics are reported.**

|  | **Time point 1** | **Time point 2*** |
| --- | --- | --- |
| *PLB - ALN* | 1.85(1.35, 2.36) | 3.38(2.33, 4.4) |
| *PLB - RIS* | 1.41(0.79, 2.03) | 2.92(1.64, 4.1) |
| *PLB - ZOL* | 3.05(2.25, 3.85) | 4.11(2.84, 5.52) |
| *PLB - IBNor* | 2.18(1.01, 3.37) | 3.32(1.04, 5.41) |
| *σ* | 1.004 | 1.6 |
| *Dres* | 81.31 | 48.07 |
| *pD* | 75.21 | 43.14 |
| *DIC* | 146.6 | 103.6 |
| *SD between treatments* | N/A | 1.36 |
| *b* | N/A | 0.91 |
| *Data points* | 87 | 47 |
| ***Note.*** ALN = Alendronate; BMD: Bone mineral density; CrI: Credible intervals; D_res_: total residual deviance; DIC: deviance information criterion; IBNor: Ibandronate 150mg; N/A: Not-applicable; pD : effective number of parameters; RIS: Risedronate; ZOL: Zoledronate; σ: between-study standard deviation  *Trial duration was imported as trial-level covariate to the class random-effect model. | | |

**Table 23: Meta-regressions on the standard random-effects model. Outcome: % change in BMD of femoral neck. Mean differences (95%CrI), heterogeneity parameter, and model-fit statistics are reported.**

|  | **Meta-regression 1 (baseline risk)** | **Meta-regression 2 (mode of administration)^a^** | **Meta-regression 3 (OP patients)^b^** | **Meta-regression 4 (patients on increased risk of fractures)^c^** |
| --- | --- | --- | --- | --- |
| *b* | -.26(-.72, .16) | -2.33(-4.68, -0.1) | .06(-1.24, 1.37) | -.28(-2.17, 1.59) |
| *PLB - ALN* | 3.05(2.45, 3.66) | 2.99(2.4, 3.58) | 2.89(2.22, 3.55) | 2.91(2.3, 3.5) |
| *PLB - RIS* | 2.18(1.44, 2.9) | 2.5(1.75, 3.23) | 2.32(1.55, 3.07) | 2.35(1.58, 3.1) |
| *PLB - ZOL* | 4.4(3.43, 5.39) | 6.09(4.11, 8.14) | 4.29(3.31, 5.27) | 4.35(3.31, 5.41) |
| *PLB - IBNor* | 2.53(1.18, 3.87) | 2.57(1.24, 3.9) | 2.5(1.21, 3.87) | 2.51(1.14, 3.86) |
| *σ* | 1.31 | 1.18 | 1.22 | 1.22 |
| *Dres* | 102.5 | 86.48 | 89.08 | 88.98 |
| *pD* | 85.17 | 77.99 | 78.32 | 78.25 |
| *DIC* | 207.1 | 170.2 | 174.6 | 174.4 |
| *Data points* | 96 | 89 | 89 | 89 |
| ***Note.*** ALN = Alendronate; CrI: Credible intervals; D_res_: total residual deviance; DIC: deviance information criterion; IBNor: Ibandronate 150mg; OP: Osteoporosis; pD : effective number of parameters; PLB: Placebo; RIS: Risedronate; ZOL: Zoledronate; σ: between-study standard deviation  ^a^Coding: 0 = oral; 1 = intravenous  ^b^Coding: 0 = % of patients with OP < 75%; 1 = % of patients with OP > 75%  ^c^Coding: 0 = % patients with increased risk of fractures < 75%; 1 = % patients with increased risk of fractures > 75% | | | | |

| **Table 24: Meta-regressions on the class-effect models of vertebral and non-vertebral fractures. Median hazard ratios (95%CrI), heterogeneity parameter and model-fit statistics are reported.** | | | | | | | | |
| --- | --- | --- | --- | --- | --- | --- | --- | --- |
|  | **Vertebral fractures** | | | | **Non-vertebral fractures** | | | |
|  | **Meta-regression 1 (baseline risk)*** | **Meta-regression 2 (mode of administration)^a^** | **Meta-regression 3 (OP patients)^b^** | **Meta-regression 4 (patients on increased risk of fractures)^c^** | **Meta-regression 1 (baseline risk)*** | **Meta-regression 2 (mode of administration)^a^** | **Meta-regression 3 (OP patients)^b^** | **Meta-regression 4 (patients on increased risk of fractures)^c^** |
| *b* | -.006(-.18, .17) | -.53(-1.68, .23) | -.61(-1.07, -.17) | .13(-.33, .52) | .007(-.24, .23) | -.006(-1.03, 1.13) | .06(-.24, .39) | -.1(-.42, .2) |
| *PLB - RIS* | .49(.35, .66) | .53(.4, .69) | .54(.43, .69) | .46(.33, .68) | .68(.52, .83) | .67(.5, .85) | .69(.52, .85) | .72(.54, .89) |
| *PLB - ALN* | .42(.31, .54) | .46(.34, .61) | .47(.37, .57) | .43(.32, .57) | .75(.61, .9) | .78(.63, .92) | .77(.62, .93) | .77(.64, .92) |
| *PLB - ZOL* | .37(.26, .48) | .53(.27, 1.85) | .45(.33, .58) | .36(.26, .5) | .71(.59, .82) | .71(.24, 1.99) | .7(.57, .84) | .73(.61, .86) |
| *PLB - IBNor* | .41(.18, .81) | .49(.2, 1.08) | .48(.24, .94) | .41(.20, .9) | .73(.5, 1.24) | .77(.45, 1.47) | .74(.51, 1.29) | .76(.52, 1.27) |
| *σ* | .18 | .17 | .12 | .19 | .1 | .07 | .1 | .09 |
| *Dres* | 56.17 | 56 | 52.59 | 57.12 | 31.06 | 31.03 | 31.69 | 31.68 |
| *pD* | 34.77 | 34.47 | 32.83 | 35.11 | 24.32 | 23.84 | 25.54 | 24.28 |
| *DIC* | 299.9 | 298.1 | 293 | 299.8 | 232.3 | 231.8 | 231.8 | 231.5 |
| *Data points* | 57 | 54 | 54 | 54 | 39 | 38 | 38 | 38 |
| ***Note.*** ALN = Alendronate; CrI: Credible intervals; D_res_: total residual deviance; DIC: deviance information criterion; IBNor: Ibandronate 150mg oral; pD : effective number of parameters; RIS: Risedronate; ZOL: Zoledronate; σ: between-study standard deviation  ^a^Coding: 0 = oral; 1 = intravenous  ^b^Coding: 0 = % of patients with OP < 75%; 1 = % of patients with OP > 75%  ^c^Coding: 0 = % patients with increased risk of fractures < 75%; 1 = % patients with increased risk of fractures > 75%  *Informative, half-normal priors were used in order to achieve numerical stability. | | | | | | | | |

**Supplementary information on post-hoc sensitivity analyses**

A sensitivity analysis was conducted on femoral neck BMD, exploring the treatment effects after removing the studies from which data was extracted from graphs (Sensitivity analysis 2 Table 21). Data were available from 36 studies one from which was a 3-arm trial for this post-hoc analysis. The model had a good fit with the data with a total residual deviance of 70.89 (data-points: 73). The direction of treatment effects and the treatment ranking remained the same, although the effects of ZOL were substantially increased MD = 4.51 (95%CrI: 3.41, 5.68). A post-hoc sensitivity analysis was conducted after removing a single outlier study^28^ (Sensitivity post-hoc 2; Table 21). Data were available from 43 studies. The model had a good fit with the data with a total residual deviance of 80.27 (data points: 87; DIC: 154). The direction of treatment effects and the treatment ranking remained the same, although the effects of ZOL were decreased MD = 3.49 (95%CrI: 2.78, 4.18). Albeit high, the between-study SD was considerably decreased after removing this single study (SD = .71). A second, post-hoc, sensitivity analysis was also performed after removing the only study which was an independent ancillary sub-study^43^ of the HORIZON trial^52^. Data were available from 43 trials. The model had a relatively good fit with the data with a total residual deviance of 90.23 (data-points: 87; DIC: 166.9). The direction of treatment effects and the treatment ranking remained the same with the effects of ZOL showing a slight increase MD = 4.075 (95%CrI: 3.23, 4.93).

A post-hoc sensitivity analysis was performed on both vertebral and non-vertebral fractures after removing the only trial which was an independent ancillary sub-study^43^ of the HORIZON trial^52^. Data were available from 26 trials for vertebral fractures. The model had a relatively good fit with the data with a total residual deviance of 54.96 (data points: 52). The direction of treatment effects and the treatment ranking remained the same with ZOL being the most effective treatment with median HR = 0.39 (95%CrI: 0.28, 0.5). Data were available from 18 trials for non-vertebral fractures. The model had a relatively good fit with the data with a total residual deviance of 29.42 (data points: 36). The direction of treatment effects and the treatment ranking remained the same with RIS being the most effective treatment with median HR = 0.69 (95%CrI: 053, 0.86) and it was followed by ZOL with median HR = 0.71 (95%CrI: 0.6, 0.81).

**Supplementary information on subgroup analysis of the main outcomes**

For vertebral fractures, the model fit of the meta-regression on the baseline risk was good with a total residual deviance of 56.17 (data-points: 57). The between-study SD was estimated to be 0.18 implying mild heterogeneity in treatment effects between RCTs. There was no evidence that treatment effect varied according to baseline risk, with the interaction term estimated to be –0.006 (95% CrI: 0.18, 0.17) while including baseline risk as an effect modifier did not improve the model fit. The model fit of the meta-regression on the mode of administration was relatively good with a total residual deviance of 56 (data-points: 54). The between-study SD was estimated to be 0.18 implying mild heterogeneity in treatment effects between RCTs. There was no evidence that treatment effect varied according to the mode of administration, with the interaction term estimated to be –0.53 (95% CrI: –1.68, 0.23) while including mode of administration as an effect modifier only slightly improved the model fit. The model fit of the meta-regression on the patients at increased risk of fractures was moderate with a total residual deviance of 57.12 (data points: 54). The between-study SD was estimated to be .19 implying mild heterogeneity in treatment effects between RCTs. There was no evidence that treatment effect varied according to baseline risk, with the interaction term estimated to be 0.13 (95% CrI: –0.33, 0.52). The model fit was not improved by including fracture risk as an effect modifier.

For non-vertebral fractures, the model fit of the meta-regression on the baseline risk was good with a total residual deviance of 31.06 (data points: 39). The between-study SD was estimated to be 0.1 implying mild heterogeneity in treatment effects between RCTs. There was no evidence that treatment effect varied according to baseline risk, with the interaction term estimated to be 0.007 (95% CrI: –0.24, 0.23) while including baseline risk as an effect modifier did not improve the model fit. The model fit of the meta-regression on the mode of administration was good with a total residual deviance of 31.03 (data-points: 38). The between-study SD was estimated to be .07 implying minimal heterogeneity in treatment effects between RCTs. There was no evidence that treatment effect varied according to mode of administration, with the interaction term estimated to be -0.006 (95% CrI: –1.04, 1.13) while including mode of administration as an effect modifier did not improve the model fit. The model fit of the meta-regression on osteoporotic status of participants was good with a total residual deviance of 31.69 (data-points: 38). The between-study SD was estimated to be 0.1 implying minimal heterogeneity in treatment effects between RCTs. There was no evidence that treatment effect varied according to osteoporosis status, with the interaction term estimated to be 0.06 (95% CrI: –0.24, 0.39) while including osteoporotic status as an effect modifier did not improve the model fit. The model fit of the meta-regression on participants’ fracture risk was good with a total residual deviance of 31.68 (data-points: 38). The between-study SD was estimated to be 0.09 implying minimal heterogeneity in treatment effects between RCTs. There was no evidence that treatment effect varied according to fractures risk, with the interaction term estimated to be -0.1 (95% CrI: –0.42, 0.2) while including fractures risk as an effect modifier did not improve the model fit.

For percentage BMD change, the model fit of the meta-regression on the baseline risk was poor with a total residual deviance of 102.5 (data-points: 96). The between-study SD was estimated to be 1.31 implying substantial heterogeneity in treatment effects between RCTs. There was no evidence that treatment effect varied according to baseline risk, with the interaction term estimated to be -0.26 (95% CrI: –0.72, 0.16) while including baseline risk as an effect modifier did not improve the model fit. The model fit of the meta-regression on the mode of administration was good with a total residual deviance of 86.48 (data-points: 89). The between-study SD was estimated to be 1.18 implying substantial heterogeneity in treatment effects between RCTs. There was evidence that the treatment effect varied according to mode of administration, with the interaction term estimated to be -2.33 (95% CrI: –4.68, -0.1) while including mode of administration as an effect modifier slightly improved the model fit. The model fit of the meta-regression on osteoporotic participants’ status was good with a total residual deviance of 89.08 (data-points: 89). The between-study SD was estimated to be 1.22 implying substantial heterogeneity in treatment effects between RCTs. There was no evidence that the treatment effect varied according to osteoporosis status, with the interaction term estimated to be 0.6 (95% CrI: –1.24, 1.37) while including osteoporosis status as an effect modifier did not improve the model fit. The model fit of the meta-regression on fractures risk was good with a total residual deviance of 88.98 (data-points: 89). The between-study SD was estimated to be 1.22 implying substantial heterogeneity in treatment effects between RCTs. There was no evidence that the treatment effect varied according to fractures risk, with the interaction term estimated to be -0.28 (95% CrI: –2.17, 1.59) while including fracture risk as an effect modifier did not improve the model fit

| **Appendix 6. Risk of bias assessment** |
| --- |
| **Figure 2. Risk of bias of individual trials included in the systematic review** |
| 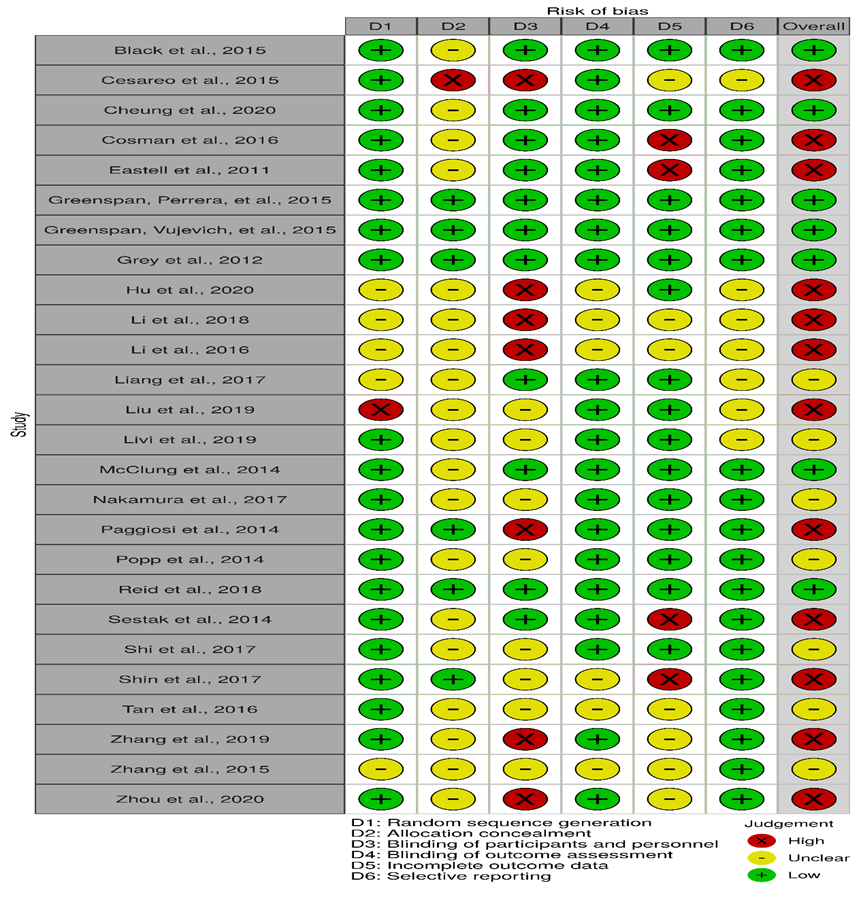 |

**Figure 3. Summary of risk of bias assessment**


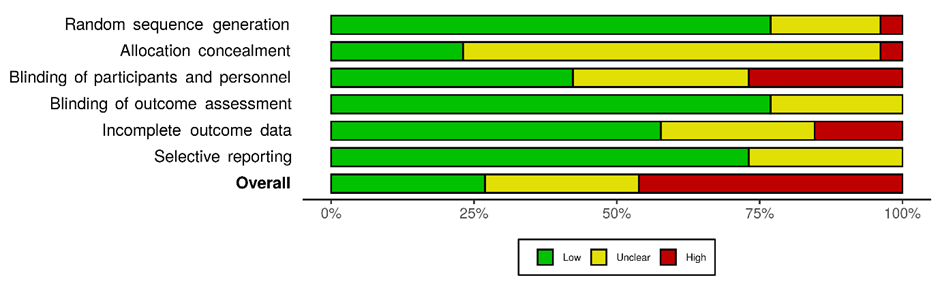


| **Appendix 7. Quality of evidence** | | | | | | | | |
| --- | --- | --- | --- | --- | --- | --- | --- | --- |
| **Table 25: Rating the quality of evidence. Outcome: Vertebral fractures** | | | | | | | | |
| **Comparisons (mixed evidence)** | **n of studies** | **Within-study bias** | **Reporting bias** | **Indirectness** | **Imprecision** | **Heterogeneity** | **Incoherence** | **Confidence rating*** |
| ALN vs IBNor | 1 | SC | undetected | NC | MC | NC | NC | Low |
| ALN vs PLB | 7 | NC | undetected | NC | NC | NC | SC | High |
| ALN vs RIS | 1 | MC | undetected | NC | SC | NC | NC | Low |
| PLB vs RIS | 8 | MC | undetected | NC | NC | NC | SC | Moderate |
| PLB vs ZOL | 9 | NC | undetected | NC | NC | NC | SC | High |
| RIS vs ZOL | 1 | NC | undetected | NC | SC | NC | NC | Moderate |
| Comparisons (indirect evidence) |  |  |  |  |  |  |  |  |
| ALN vs ZOL | - | NC | undetected | NC | SC | NC | NC | High |
| IBNor vs PLB | - | SC | undetected | NC | NC | NC | NC | High |
| IBNor vs RIS | - | SC | undetected | NC | MC | NC | NC | Low |
| IBNor vs ZOL | - | NC | undetected | NC | MC | NC | NC | Low |
| ***Note.*** ALN: Alendronate; IBNor: Ibandronate 150mg; MC: Major concerns; NC: No concerns; PLB: Placebo; RIS: Risedronate; SC: Some concerns; ZOL: Zoledronate  *Clinical significance was indicated by conventional thresholds of .8 and 1.25 in HRs for vertebral fractures and 1/2 SD of control arms at baseline for BMD femoral neck. | | | | | | | | |

| **Table 26: Rating the quality of evidence. Outcome: % change in Femoral neck BMD** | | | | | | | | |
| --- | --- | --- | --- | --- | --- | --- | --- | --- |
| **Comparisons (mixed evidence)** | **n of studies** | **within-study bias** | **Reporting bias** | **Indirectness** | **Imprecision** | **Heterogeneity** | **Incoherence** | **Confidence rating*** |
| ALN vs IBNor | 2 | NC | undetected | NC | NC | NC | NC | High |
| ALN vs PLB | 17 | SC | undetected | NC | NC | NC | NC | High |
| ALN vs RIS | 4 | NC | undetected | NC | NC | NC | NC | High |
| ALN vs ZOL | 1 | NC | undetected | NC | NC | SC | MC | Low |
| IBNor vs PLB | 2 | NC | undetected | NC | NC | NC | NC | High |
| IBNor vs RIS | 1 | NC | undetected | NC | NC | NC | NC | High |
| PLB vs RIS | 11 | SC | undetected | NC | NC | NC | NC | High |
| PLB vs ZOL | 7 | NC | undetected | NC | NC | NC | SC | High |
| RIS vs ZOL | 1 | NC | undetected | NC | NC | SC | NC | High |
| Comparisons (indirect evidence) | |  |  |  |  |  |  |  |
| IBNor vs ZOL | - | NC | undetected | NC | NC | SC | MC | Low |
| ***Note.*** ALN: Alendronate; IBNor: Ibandronate 150mg; MC: Major concerns; NC: No concerns; PLB: Placebo; RIS: Risedronate; SC: Some concerns; ZOL: Zoledronate  *Clinical significance was indicated by conventional thresholds of .8 and 1.25 in HRs reported and 0.5 SD of control arms at baseline for continuous outcomes. | | | | | | | | |

| **Table 27: Network plots and post-hoc risk of bias summaries of those trials accounting for the quality of evidence rating (outcomes: BMD femoral neck and vertebral fractures)** | | |
| --- | --- | --- |
| **Outcomes** | **Network plots** | **Risk of bias bar chart** |
| BMD femoral neck | **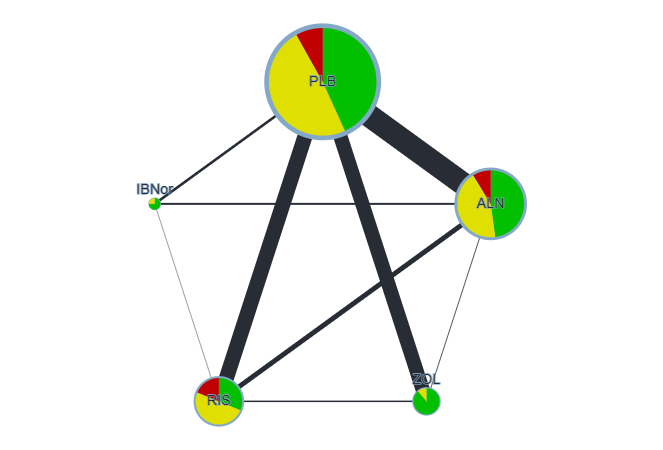** | **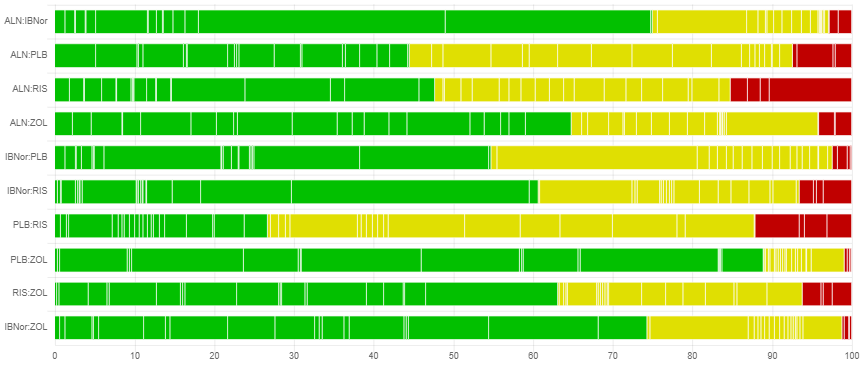** |
| Vertebral fractures | **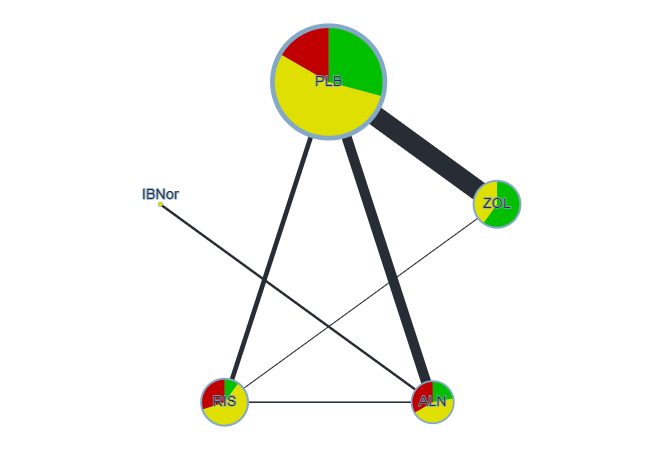** | **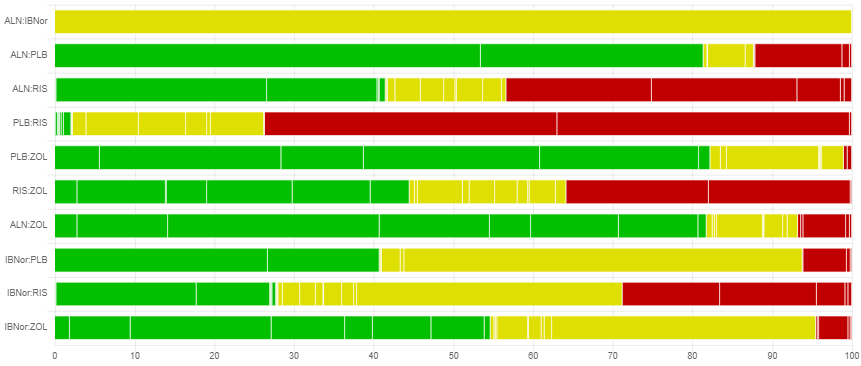** |
| ***Note.*** ALN: Alendronate; IBN-or: Ibadrondronate 150mg; RIS: Risedronate; ZOL: Zoledronate.  Network plots: nodes’ size is proporionate to the number of studies informing each comparison while edges’ thinckness is proportionate to the sample size informing each comparison. | | |

**Appendix 8. Assessment of inconsistency**

**Table 28. Assessment of inconsistency on the main outcomes (vertebral fractures and % change in BMD femoral neck).**

| **% change BMD femoral neck** | | **Vertebral fractures** | |
| --- | --- | --- | --- |
| **Comparisons** | **Mean(95%CrI)** | **Comparisons** | **Mean(95%CrI)** |
| ALN - IBNor |  | PLB - RIS |  |
| - direct | -0.19 (-2.2, 1.8) | - direct | -0.61 (-0.93, -0.34) |
| - indirect | -0.88 (-2.7, 0.98) | - indirect | -1.4 (-2.9, 0.18) |
| - network | -0.55 (-1.9, 0.78) | - network | -0.64 (-0.96, -0.38) |
| *p* = .6 | | *p* = .33 | |
| ALN - PLB |  | PLB - ZOL |  |
| - direct | -3.1 (-3.7, -2.5) | - direct | -1.1 (-1.4, -0.81) |
| - indirect | -3. (-4.3, -1.7) | - indirect | -0.32 (-1.8, 1.2) |
| - network | -3.1 (-3.6, -2.6) | - network | -1.1 (-1.4, -0.79) |
| *p* = .89 | | *p* = .304 | |
| ALN - RIS |  | RIS - ZOL |  |
| - direct | -0.73 (-2.0, 0.56) | - direct | 0.29 (-1.2, 1.8) |
| - indirect | -0.59 (-1.5, 0.32) | - indirect | -0.48 (-0.89, -0.068) |
| - network | -0.63 (-1.4, 0.096) | - network | -0.43 (-0.81, -0.025) |
| *p* = .85 | | *p* = .315 | |
| IBNor - PLB |  |  |  |
| - direct | -2.2 (-4.0, -0.45) |  |  |
| - indirect | -2.9 (-5., -0.86) |  |  |
| - network | -2.5 (-3.8, -1.2) |  |  |
| *p* = .61 | |  |  |
| PLB - RIS |  |  |  |
| - direct | 2.4 (1.7, 3.2) |  |  |
| - indirect | 2.4 (1.2, 3.7) |  |  |
| - network | 2.4 (1.8, 3.1) |  |  |
| *p* = .98 | |  |  |
| PLB - ZOL |  |  |  |
| - direct | 3.8 (2.8, 4.7) |  |  |
| - indirect | 3.4 (1.2, 5.6) |  |  |
| - network | 3.7 (2.9, 4.6) |  |  |
| *p* = .755 | |  |  |
| RIS - ZOL |  |  |  |
| - direct | 1.0 (-1.1, 3.2) |  |  |
| - indirect | 1.4 (0.21, 2.5) |  |  |
| - network | 1.3 (0.28, 2.3) |  |  |
| *p* = .751 | |  |  |
| ***Note.*** ALN = Alendronate; CrI: Credible intervals; D_res_: total residual deviance; DIC: deviance information criterion; IBNor: Ibandronate 150mg oral; pD : effective number of parameters; RIS: Risedronate; ZOL: Zoledronate; σ: between-study standard deviation | | | |

**Appendix 9. Data used to populate the network meta-analyses of the main outcomes**

**Table 29. Summary of trials included in the NMA of vertebral fractures**

| Author and year of study  publication | Study  duration  (years) | Treatments^a^ | | Events | | Number of participants | |
| --- | --- | --- | --- | --- | --- | --- | --- |
|  |  | Arm 1 | Arm 2 | Arm 1 | Arm 2 | Arm 1 | Arm 2 |
| Cohen et al., 1999 | 1 | 1 | 2 | 5 | 2 | 35 | 34 |
| Fogelman et al., 2000 | 2 | 1 | 2 | 17 | 8 | 125 | 112 |
| Harris et al., 1999 | 3 | 1 | 2 | 93 | 61 | 678 | 696 |
| Reginster et al., 2000 | 3 | 1 | 2 | 89 | 53 | 346 | 344 |
| Hooper et al., 2005 | 2 | 1 | 2 | 10 | 10 | 125 | 129 |
| Reid et al., 2000 | 1 | 1 | 2 | 9 | 3 | 60 | 60 |
| Boonen et al., 2009 | 2 | 1 | 2 | 0 | 1 | 80 | 191 |
| Ringe et al., 2006 | 1 | 1 | 2 | 20 | 8 | 158 | 158 |
| Liberman et al., 1995 | 3 | 1 | 3 | 22 | 5 | 355 | 175 |
| Orwoll et al., 2000 | 2 | 1 | 3 | 7 | 1 | 94 | 146 |
| Black et al., 1996 | 3 | 1 | 3 | 192 | 83 | 965 | 981 |
| Cummings et al., 1998 | 4 | 1 | 3 | 78 | 43 | 2077 | 2057 |
| Dursun et al., 2001 | 1 | 1 | 3 | 14 | 12 | 35 | 38 |
| Carfora et al., 1998 | 2.5 | 1 | 3 | 4 | 1 | 34 | 34 |
| Boonen et al., 2012 | 2 | 1 | 4 | 28 | 9 | 574 | 533 |
| Black et al., 2007 | 3 | 1 | 4 | 84 | 19 | 3861 | 3875 |
| Lyles et al., 2007 | 3 | 1 | 4 | 39 | 21 | 1062 | 1065 |
| Muscoso et al., 2004 | 1 | 2 | 3 | 0 | 2 | 100 | 1000 |
| Reid et al., 2009 | 1 | 2 | 4 | 3 | 5 | 381 | 378 |
| Miller et al., 2008 | 1 | 3 | 5 | 5 | 5 | 859 | 874 |
| Greenspan, Perrera, et al., 2015 | 2 | 1 | 4 | 8 | 6 | 92 | 89 |
| Popp et al., 2014 | 3 | 1 | 4 | 5 | 1 | 55 | 55 |
| Zhang et al., 2019 | 1 | 1 | 4 | 5 | 0 | 51 | 50 |
| Zhang et al., 2015 | 1 | 1 | 3 | 2 | 0 | 108 | 107 |
| Nakamura et al., 2017 | 2 | 1 | 4 | 29 | 10 | 327 | 330 |
| Reid et al., 2018 | 6 | 1 | 4 | 49 | 23 | 1000 | 1000 |
| Hu et al., 2020 | 1 | 1 | 4 | 13 | 2 | 121 | 121 |
| ^a^Treatments are coded as 1 = Placebo; 2 = Risedronate; 3 = Alendronate; 4 = Zoledronate; and 5 = Ibandronate 150 mg | | | | | | | |

**Table 30. Summary of trials included in the NMA of non-vertebral fractures**

| Author and year of study  publication | Study  duration  (years) | Treatments^a^ | | Events | | Number of participants | |
| --- | --- | --- | --- | --- | --- | --- | --- |
|  |  | Arm 1 | Arm 2 | Arm 1 | Arm 2 | Arm 1 | Arm 2 |
| Fogelman et al., 2000 | 3 | 1 | 2 | 13 | 7 | 125 | 112 |
| Harris, 1999 | 3 | 1 | 2 | 52 | 33 | 815 | 812 |
| Reginster et al., 2000 | 2 | 1 | 2 | 51 | 36 | 406 | 406 |
| Hooper et al., 2005 | 1 | 1 | 2 | 6 | 5 | 125 | 129 |
| Ringe et al., 2006 | 4 | 1 | 2 | 17 | 10 | 158 | 158 |
| Black et al., 1996 | 3 | 1 | 3 | 148 | 122 | 1005 | 1022 |
| Cummings et al., 1998 | 4 | 1 | 3 | 294 | 261 | 2077 | 2057 |
| Orwoll et al., 2000 | 2 | 1 | 3 | 5 | 6 | 94 | 146 |
| Pols et al., 1999 | 1 | 1 | 3 | 37 | 19 | 958 | 950 |
| Bone et al., 2000 | 2 | 1 | 3 | 4 | 5 | 50 | 92 |
| Black et al., 2007 | 1 | 1 | 4 | 388 | 292 | 3861 | 3875 |
| Lyles et al., 2007 | 3 | 1 | 4 | 107 | 79 | 1062 | 1065 |
| Miller et al., 2008 | 1 | 3 | 5 | 12 | 14 | 859 | 874 |
| Popp et al., 2014 | 3 | 1 | 4 | 7 | 8 | 55 | 55 |
| Nakamura et al., 2017 | 2 | 1 | 4 | 37 | 20 | 331 | 330 |
| Zhang et al., 2015 | 1 | 1 | 3 | 2 | 1 | 108 | 107 |
| Grey et al., 2017 | 1 | 1 | 4 | 2 | 1 | 34 | 41 |
| Reid et al., 2018 | 6 | 1 | 4 | 148 | 101 | 1000 | 1000 |
| Cheung et al., 2020 | 2 | 1 | 4 | 0 | 1 | 37 | 39 |
| ^a^Treatments are coded as 1 = Placebo; 2 = Risedronate; 3 = Alendronate; 4 = Zoledronate; and 5 = Ibandronate 150 mg | | | | | | | |

**Table 31. Summary of trials included in the NMA of BMD femoral neck.**

| Author and year of  study publication | Study  duration  (years) | Treatments^a^ | | | % change in  BMD | | | SE %  change in  BMD | | | Number of  participants | | | Mean difference | |
| --- | --- | --- | --- | --- | --- | --- | --- | --- | --- | --- | --- | --- | --- | --- | --- |
|  |  | Arm 1 | Arm 2 | Arm 3 | Arm 1 | Arm 2 | Arm 3 | Arm 1 | Arm 2 | Arm 3 | Arm 1 | Arm 2 | Arm 3 | % change  in BMD | SE |
| Adami et al., 1995 | 2 | 1 | 2 | NA | -2.58 | 1.19 | NA | .89 | .88 | NA | 62 | 61 | NA | NA | NA |
| Bone et al., 2000 | 2 | 1 | 2 | NA | -0.6 | 2.9 | NA | .6 | .5 | NA | 46 | 87 | NA | NA | NA |
| Dursun et al., 2001 | 1 | 1 | 2 | NA | 2.33 | 3.75 | NA | .73 | 1 | NA | 35 | 38 | NA | NA | NA |
| Pols et al., 1999 | 1 | 1 | 2 | NA | -.2 | 2.3 | NA | .15 | .15 | NA | 884 | 863 | NA | NA | NA |
| Greenspan et al.,  2003 | 3 | 1 | 2 | NA | -.65 | 4.2 | NA | .53 | .59 | NA | 93 | 93 | NA | NA | NA |
| Orwoll et al., 2000 | 2 | 1 | 2 | NA | -.1 | 2.5 | NA | .5 | .4 | NA | 81 | 128 | NA | NA | NA |
| Saag et al., 1998 | 1 | 1 | 2 | NA | -1.2 | 1 | NA | .4 | .4 | NA | 142 | 145 | NA | NA | NA |
| Klotz et al., 2013 | 1 | 1 | 2 | NA | -2.06 | 1.65 | NA | .78 | 1.12 | NA | 53 | 45 | NA | NA | NA |
| Fogelman et al.,  2000 | 2 | 1 | 3 | NA | -1 | 1.3 | NA | .32 | .44 | NA | 180 | 175 | NA | NA | NA |
| Harris et al., 1999 | 3 | 1 | 3 | NA | -1.2 | 1.6 | NA | .45 | .6 | NA | 417 | 457 | NA | NA | NA |
| Leung et al., 2005 | 1 | 1 | 3 | NA | 1.1 | 1.8 | NA | .9 | .7 | NA | 34 | 31 | NA | NA | NA |
| Cohen et al., 1999 | 1 | 1 | 3 | NA | -2.94 | -1.04 | NA | .84 | .94 | NA | 36 | 34 | NA | NA | NA |
| Reid et al., 2000 | 1 | 1 | 3 | NA | -.29 | 1.63 | NA | .5 | .62 | NA | 43 | 52 | NA | NA | NA |
| Boonen et al., 2009 | 2 | 1 | 3 | NA | .73 | 1.71 | NA | .34 | .25 | NA | 93 | 191 | NA | NA | NA |
| Choo et al., 2011 | 2 | 1 | 3 | NA | -5.56 | -2.55 | NA | 2.92 | 2.89 | NA | 52 | 52 | NA | NA | NA |
| Taxel et al., 2010 | 1 | 1 | 3 | NA | -2 | 0 | NA | .61 | .61 | NA | 20 | 20 | NA | NA | NA |
| McClung et al.,  2009 | 2 | 1 | 4 | NA | -1.35 | 1.64 | NA | .29 | .31 | NA | 202 | 181 | NA | NA | NA |
| Boonen et al., 2012 | 2 | 1 | 4 | NA | .1 | 3.4 | NA | .58 | .6 | NA | 63 | 56 | NA | NA | NA |
| McClung et al.,  2009 | 1 | 1 | 5 | NA | -.73 | 1.09 | NA | .46 | .33 | NA | 83 | 77 | NA | NA | NA |
| Sarioglu et al.,  2006 | 1 | 2 | 3 | NA | 3.7 | 2.6 | NA | .96 | .6 | NA | 25 | 25 | NA | NA | NA |
| Miller et al., 2008 | 1 | 2 | 5 | NA | 2.3 | 2.1 | NA | .07 | .06 | NA | 822 | 836 | NA | NA | NA |
| Reid et al., 2009 | 1 | 3 | 4 | NA | .39 | 1.4 | NA | .25 | .26 | NA | 374 | 373 | NA | NA | NA |
| Black et al., 1996 | 3 | 1 | 2 | NA | -.31 | 3.54 | NA | .18 | .17 | NA | 1005 | 1022 | NA | 4.1 | .25 |
| Cummings et al.,  1998 | 4 | 1 | 2 | NA | -.8 | 3.6 | NA | .16 | .16 | NA | 2218 | 2214 | NA | 4.6 | .23 |
| Greenspan et al.,  2002 | 2 | 1 | 2 | NA | -.36 | 2.84 | NA | .06 | .35 | NA | 164 | 163 | NA | 3.4 | .5 |
| Liberman et al.,  1995 | 3 | 1 | 2 | NA | -1.28 | 4.65 | NA | .3 | .47 | NA | 397 | 196 | NA | 5.9 | .5 |
| Hooper et al., 2005 | 2 | 1 | 3 | NA | -2.43 | 2.29 | NA | .33 | .2 | NA | 125 | 125 | NA | 3.3 | .27 |
| Reginster et al.,  2000 | 3 | 1 | 3 | NA | -.97 | 2.09 | NA | .37 | .38 | NA | 407 | 407 | NA | 3.1 | .7 |
| Lyles et al., 2007 | 3 | 1 | 4 | NA | NA | NA | NA | NA | NA | NA | 1062 | 1065 | NA | 2.9 | 1.31 |
| Black et al., 2007 | 3 | 1 | 4 | NA | -.04 | 5.06 | NA | .16 | .15 | NA | 3083 | 3067 | NA | 5.06 | .15 |
| Rosen et al., 2005 | 1 | 2 | 3 | NA | 1.6 | .9 | NA | .21 | .21 | NA | 454 | 438 | NA | -.7 | .28 |
| Reid et al., 2006 | 1 | 2 | 3 | NA | 2.25 | 1.67 | NA | .18 | .18 | NA | 424 | 430 | NA | -.56 | .27 |
| Cosman et al., 2016 | 1 | 1 | 2 | NA | -1.23 | -.06 | NA | .43 | .43 | NA | 66 | 68 | NA | NA | NA |
| Eastell et al., 2011 | 1 | 1 | 2 | NA | .14 | 2.61 | NA | .43 | .45 | NA | 49 | 52 | NA | NA | NA |
| Greenspan, Perrera et al., 2015 | 1 | 1 | 4 | NA | -1.28 | 2.31 | NA | .65 | .79 | NA | 71 | 69 | NA | NA | NA |
| Greenspan, Vujevich et al., 2015 | 2 | 1 | 3 | NA | -2.15 | .37 | NA | .64 | .64 | NA | 47 | 48 | NA | NA | NA |
| McClung et al., 2014 | 1 | 1 | 2 | NA | -1.1 | 1.2 | NA | .45 | .45 | NA | 50 | 51 | NA | NA | NA |
| Nakamura et al., 2017 | 2 | 1 | 4 | NA | -.46 | 3.58 | NA | .32 | .39 | NA | 198 | 172 | NA | NA | NA |
| Popp et al., 2014 | 3 | 1 | 4 | NA | -.98 | 2.76 | NA | .85 | .89 | NA | 47 | 48 | NA | NA | NA |
| Shi et al., 2017 | 1 | 1 | 2 | NA | -.15 | 1.63 | NA | .02 | .07 | NA | 77 | 79 | NA | NA | NA |
| Shin et al., 2017 | 1 | 1 | 5 | NA | -1.2 | 1.7 | NA | .87 | .61 | NA | 73 | 76 | NA | NA | NA |
| Zhang et al., 2015 | 1 | 1 | 2 | NA | .4 | 3.3 | NA | .68 | .71 | NA | 101 | 95 | NA | NA | NA |
| Tan et al., 2016 | 3 | 2 | 4 | NA | 6.3 | 13.5 | NA | .31 | .94 | NA | 53 | 52 | NA | NA | NA |
| Paggiosi et al., 2014 | 2 | 2 | 5 | 3 | 3.97 | 3 | 1.91 | .61 | .51 | .65 | 31 | 32 | 31 | NA | NA |
| ***Note.*** NA: Not applicable; SE: Standard error  ^a^Treatments are coded as 1 = Placebo; 2 = Alendronate; 3 = Risedronate; 4 = Zoledronate; 5 = Ibandronic acid 150 mg | | | | | | | | | | | | | | | |

**Table 32. Summary of trials included in the NMA of wrist fractures**

| Author and year of study  publication | Study  duration  (years) | Treatments^a^ | | | Events | | | Number of participants | | |
| --- | --- | --- | --- | --- | --- | --- | --- | --- | --- | --- |
|  |  | Arm1 | Arm2 | Arm3 | Arm1 | Arm2 | Arm3 | Arm1 | Arm2 | Arm3 |
| Shin et al., 2017 | 1 | 1 | 4 | NA | 1 | 0 | NA | 76 | 81 | NA |
| Li et al., 2016 | 1 | 1 | 5 | NA | 2 | 0 | NA | 30 | 30 | NA |
| Harris et al., 1999 | 3 | 1 | 2 | NA | 22 | 14 | NA | 815 | 812 | NA |
| Reginster et al., 2000 | 3 | 1 | 2 | NA | 21 | 15 | NA | 406 | 406 | NA |
| Black et al., 1996 | 3 | 1 | 3 | NA | 41 | 22 | NA | 1005 | 1022 | NA |
| Cummings et al., 1998 | 4 | 1 | 3 | NA | 70 | 83 | NA | 2218 | 2214 | NA |
| McClung et al., 2009 | 1 | 1 | 4 | NA | 0 | 1 | NA | 83 | 77 | NA |
| Lester et al., 2008 | 2 | 1 | 4 | NA | 1 | 1 | NA | 19 | 21 | NA |
| Muscoco et al., 2004 | 1 | 2 | 3 | NA | 0 | 1 | NA | 100 | 1000 | NA |
| Paggiosi et al., 2014 | 1 | 3 | 4 | 2 | 2 | 1 | 0 | 57 | 57 | 58 |
| ^a^Treatments are coded as 1 = Placebo; 2 = Risedronate; 3 = Alendronate; 4 = Ibandronate 150 mg; 5 = Zoledronate | | | | | | | | | | |

**Table 33. Summary of trials included in the NMA of hip fractures**

| Author and year of study  publication | Study  duration  (years) | Treatments^a^ | | Events | | Number of participants | |
| --- | --- | --- | --- | --- | --- | --- | --- |
|  |  | Arm1 | Arm2 | Arm1 | Arm2 | Arm1 | Arm2 |
| Li et al., 2016 | 1 | 1 | 4 | 2 | 0 | 30 | 30 |
| Liu et al., 2019 | 2 | 1 | 4 | 11 | 21 | 129 | 353 |
| Nakamura et al., 2017 | 2 | 1 | 4 | 3 | 2 | 331 | 330 |
| Reid et al., 2018 | 6 | 1 | 4 | 12 | 8 | 1000 | 1000 |
| McClung et al., 2001 | 3 | 1 | 2 | 46 | 32 | 1821 | 1812 |
| Harris et al., 1999 | 3 | 1 | 2 | 15 | 12 | 815 | 812 |
| Reginster et al., 2009 | 3 | 1 | 2 | 11 | 9 | 406 | 406 |
| Black et al., 1996 | 3 | 1 | 3 | 22 | 11 | 1005 | 1022 |
| Cummings et al., 1998 | 4 | 1 | 3 | 24 | 19 | 2218 | 2214 |
| Greenspan et al., 2002 | 2 | 1 | 3 | 4 | 2 | 164 | 163 |
| Black et al., 2007 | 3 | 1 | 4 | 88 | 52 | 3861 | 3875 |
| Lyles et al., 2007 | 3 | 1 | 4 | 33 | 23 | 1062 | 1065 |
| Lester et al., 2008 | 2 | 1 | 5 | 0 | 1 | 19 | 21 |
| Muscoco et al., 2004 | 1 | 2 | 3 | 0 | 1 | 100 | 1000 |
| ^a^Treatments are coded as 1 = Placebo; 2 = Risedronate; 3 = Alendronate; 4 = Zoledronate; 5 = Ibandronate 150 mg | | | | | | | |

**Appendix 10. List of the excluded studies**

| **Table 34. List of excluded studies with reasons** | | |
| --- | --- | --- |
| **Author** | **Title** | **Reasons** |
| Rubin et al., 2017 | Efficacy of treatment with slow-release sodium fluoride versus alendronate on bone mineral density and fractures in postmenopausal women | //conference abstract - not enough information// |
| Abay et al., 2017 | Bone microarchitecture is preserved in men with prostate cancer on androgen deprivation therapy | //conference abstract//outcomes out of scope// |
| Aitken et al., 2018 | A protocol for a multicentre, randomised, double-blind, placebo-controlled trial to compare the effect of annual infusions of zoledronic acid to placebo on knee structural change and knee pain over 24 months in knee osteoarthritis patients – ZAP2 | //protocol// |
| Al-Bogami et al., 2015 | Favorable therapeutic response of osteoporosis patients to treatment with intravenous zoledronate compared with oral alendronate | //study design out of scope// |
| Aro et al., 2018 | A long-lasting bisphosphonate partially protects periprosthetic bone, but does not enhance initial stability of uncemented femoral stems: A randomized placebo-controlled trial of women undergoing total hip arthroplasty | //population out of scope// |
| Asaoka et al., 2016 | Efficacy of alfacalcidol and alendronate on lumbar bone mineral density in osteoporotic patients using proton pump inhibitors | //non-licenced use of drug// |
| Aviles et al., 2017 | Prolonged Use of Zoledronic Acid (4 Years) Did Not Improve Outcome in Multiple Myeloma Patients | //non-licenced use of drug // comparison out of scope// |
| Bala et al., 2014 | Risedronate slows or partly reverses cortical and trabecular microarchitectural deterioration in postmenopausal women | //outcomes out of scope// |
| Bell et al., 2017 | Potential Usefulness of BMD and Bone Turnover Monitoring of Zoledronic Acid Therapy Among Women With Osteoporosis: secondary Analysis of Randomized Controlled Trial Data | //outcomes out of scope// |
| Bell et al., 2018 | Serial measurement of BMD and bone turnover to detect a treatment response to zoledronic acid: the HORIZON-PFT Trial | //parallel presentation to the above//conference poster |
| Bilek et al., 2016 | Protocol for a randomized controlled trial to compare bone-loading exercises with risedronate for preventing bone loss in osteopenic postmenopausal women | //outcomes out of scope//non-licenced use of drug// |
| Black et al., 2017 | The incidence and predictors of acute phase response to zoledronic acid in Asian compared to non-Asian women in the horizon pivotal fracture trial | //outcomes out of scope// conference paper// |
| Cai et al., 2018 | A multicentre randomised controlled trial of zoledronic acid for osteoarthritis of the knee with bone marrow lesions | //conference abstract //parallel publication// |
| Cai et al., 2018 | Zoledronic acid plus prednisolone versus zoledronic acid alone or placebo in the treatment of knee osteoarthritis: a randomised trial | //conference abstract//outcomes out of scope// |
| Cecelja et al., 2015 | A pilot study to assess effects of alendronic acid on aortic calcification and stiffness in postmenopausal women | //conference abstract //outcomes out of scope// |
| Chen et al., 2016 | Effects of zoledronic acid on bone fusion in osteoporotic patients after lumbar fusion | //population out of scope// |
| Chen et al., 2019 | Effect of co-administration of alendronate and allan sodium phosphate for the management of osteoporosis | //comparison out of scope// |
| Coates et al., 2017 | Clinical efficacy of oral alendronate in ankylosing spondylitis: a randomised placebo-controlled trial | //non-licenced use of drug//outcomes out of scope// |
| Cosman et al., 2014 | Reassessment of fracture risk in women after 3 years of treatment with zoledronic acid: when is it reasonable to discontinue treatment? | //outcomes out of scope// |
| Cross et al., 2018 | Efficacy of zoledronate in treating osteonecrosis of the femoral head: a randomized controlled trial | //non-licenced use of the drug//outcomes out of scope// |
| Dalbeth et al., 2014 | Zoledronate for prevention of bone erosion in tophaceous gout: a randomised, double-blind, placebo-controlled trial | //non-licenced use of drug// |
| Duckworth et al., 2018 | Effect of early bisphosphonate treatment on fracture healing: the fracture and bisphosphonate (FAB) study | //parallel publication//conference abstract// |
| Duckworth 2019 | The FAB (fractures and bisphosphonates) trial: a multicenter, double-blind, randomized controlled trial on the effect of alendronic acid on healing and clinical outcomes of wrist fractures | //conference abstract// |
| Eastell 2015 | Relationship Between Pretreatment Rate of Bone Loss and Bone Density Response to Once‐Yearly ZOL: HORIZON‐PFT Extension Study | //outcomes out of scope// |
| García-Sanz, 2015 | Zoledronic acid as compared with observation in multiple myeloma patients at biochemical relapse: results of the randomized AZABACHE Spanish trial | //outcomes out of scope// |
| Goenka 2018 | Effect of early treatment with zoledronic acid on prevention of bone loss in patients with acute spinal cord injury: a randomized controlled trial | //comparison and outcomes out of scope// |
| Gossiel, 2017 | Offset of effect of oral bisphosphonates on tartrate-resistant acid phosphatase in postmenopausal osteoporosis: the TRIO Study | //conference abstract//outcomes out of scope//parallel publication// |
| Gossiel, 2019 | The effect of bisphosphonates on bone turnover and bonebalance in postmenopausalwomenwith osteoporosis: the T-score bonemarker approach in the trio study | //conference paper//outcomes out of scope// |
| Gossiel 2020 | The effect of bisphosphosphonates on bone turnover and bone balance in postmenopausal women with osteoporosis: the T-score bone marker approach in the TRIO study | //outcomes out of scope// |
| Gralow, 2020 | Phase III Randomized Trial of Bisphosphonates as Adjuvant Therapy in Breast Cancer: S0307 | //non-licenced use of drug// |
| Greenspan, 2014 | Zoledronic Acid in Frail Elders to Strengthen Bone: Three Year Results from ZEST Trial | //conference paper//parallel publication// |
| Greenspan, 2020 | Abaloparatide followed by alendronate in women 80 years with osteoporosis: post hoc analysis of ACTIVExtend | //comparison out of scope// |
| Greenspan, 2014 | Against all odds: results from the zest trial in long term care residents | //conference paper//parallel publication// |
| Hagino, 2018 | Monthly oral ibandronate 100 mg is as effective as monthly intravenous ibandronate 1 mg in patients with various pathologies in the MOVEST study | //non-licenced use of drug// |
| Hagino, 2017 | The effect of monthly intravenous (IV) ibandronate (IBN) 1mg on bone mineral density (BMD) gains in the mover study: additional treat-to-target analysis | //conference paper//non-licenced use of drug// |
| Hagino, 2017b | Association between total Hip bone mineral density at baseline and vertebral fracture incidence in the MOVER study | //non-licenced use of drug// |
| Hagino, 2019 | Effectiveness of Intravenous Ibandronate on Bone Mineral Density in Patient with Osteoporosis Treated with Oral Bisphosphonate Low-responders -MOVEMENT Study | //non-licenced use of drug// |
| Hagino, 2014 | Increased bone mineral density with monthly intravenous ibandronate contributes to fracture risk reduction in patients with primary osteoporosis: three-year analysis of the MOVER study | //non-licenced use of drug// |
| Hashimoto, 2019 | MONTHLY IBANDRONATE TREATMENTS RAPIDLY SUPPRESS BONE RESORPTION MARKERS WITHOUT EXCESS | //conference paper//outcomes out of scope//non-licenced use of drug// |
| Hassler, 2015 | Effects of long-term alendronate treatment on postmenopausal osteoporosis bone material properties | //outcomes out of scope// |
| Huang 2017 | Bone turnover and periprosthetic bone loss after cementless total hip arthroplasty can be restored by zoledronic acid: a prospective, randomized, open-label, controlled trial | //outcomes out of scope//non-licenced use of drug// |
| Hyuk, 2017 | Combination therapy with raloxifene and alendronate for treatment of osteoporosis in elderly women | //non-licenced use of drug// |
| Ikeda, 2020 | Comparison of once-weekly teriparatide and alendronate against new osteoporotic vertebral fractures at week 12 | //comparison out of scope// |
| Ito, 2018 | The effect of monthly oral ibandronate (IBN) on BMD gains in the movest study: additional treatto-target analysis | //conference paper//non-licenced use of drug// |
| Ito, 2015 | Monthly oral ibandronate 100mg is as effective as monthly intravenous ibandronate 1mg in Japanese patients with primary osteoporosis: the phase III MOVEST study | //conference paper//non-licenced use of drug// |
| Ito, 2015 | Clinical efficacy of monthly oral ibandronate 100 mg in Japanese patients with primary osteoporosis: the phase III movest study | //conference paper//non-licenced use of drug// |
| Ito, 2018 | The effect of once-yearly zoledronic acid on hip structural and biomechanical properties derived using computed tomography (CT) in Japanese women with osteoporosis | //outcomes out of scope// |
| Ito, 2017 | Effect of monthly intravenous ibandronate injections on vertebral or non-vertebral fracture risk in Japanese patients with high-risk osteoporosis in the MOVER study | //non-licenced use of drug// |
| Iwamoto, 2014 | The effectiveness of mono or combined osteoporosis drug therapy against bone mineral density loss around femoral implants after total hip arthroplasty | //non-licenced use of drug// |
| Jacobson, 2019 | Alendronate improves bone mineral density in perinatally HIV-infected children and adolescents with low bone mineral density for age | //study design and outcomes out of scope// |
| Gossiel,2014 | The effect of bisphosphonate treatment on bone turnover and bone balance in postmenopausal women with osteoporosis | //conference abstract//outcomes out of scope// |
| Jaroma, 2015 | Effect of one-year post-operative alendronate treatment on periprosthetic bone after total knee arthroplasty. A seven-year randomised controlled trial of 26 patients | //outcomes out of scope// |
| Jiang, 2015 | Intravenous Zoledronic Acid Versus Oral Alendronate in the Treatment of Osteoporosis in Older Adults | //conference paper//not enough information to decide eligibility//authors' emails are not provided// |
| Johansson, 2017 | The effect of alendronate on vertebral fracture risk is independent of baseline frax fracture probability: a post HOC analysis of the fit study | //post-hoc analysis//outcomes out of scope// |
| Kamal, 2017 | Evaluation of alendronate treatment in patients with aseptic necrosis of the femoral head | //population and outcomes out of scope// |
| Kamba, 2016 | A phase III multicenter, randomized, controlled study of combined androgen blockade with versus without zoledronic acid in prostate cancer patients with metastatic bone disease: results of the ZAPCA trial | //population and comparison out of scope// |
| Kamba, 2015 | A phase III, multicenter, randomized, controlled study of maximum androgen blockade with vs without zoledronic acid in prostate cancer patients with metastatic bone disease: results of main secondary endpoints in ZAPCA trial | //population and comparison out of scope// |
| Cai et al., 2020 | Once-yearly zoledronic acid and change in abdominal aortic calcification over 3 years in postmenopausal women with osteoporosis: results from the HORIZON Pivotal Fracture Trial | //outcomes out of scope// |
| Cosman, 2020 | T-Score as an Indicator of Fracture Risk During Treatment With Romosozumab or Alendronate in the ARCH Trial | //comparison out of scope// |
| Karimi, 2018 | Alendronate improves fasting plasma glucose and insulin sensitivity, and decreases insulin resistance in prediabetic osteopenic postmenopausal women: a randomized triple-blind clinical trial | //outcomes out of scope// |
| Khabbazi, 2014 | Alendronate for prevention of bone loss in ankylosing spondylitis | //population out of scope//conference abstract// |
| Khabbazi, 2014 | Alendronate effect on the prevention of bone loss in early stages of ankylosing spondylitis: a randomized, double-blind, placebo-controlled pilot study | //population out of scope// |
| Khan, 2014 | A randomized, double-blind, placebo-controlled study to evaluate the effects of alendronate on bone mineral density and bone remodelling in perimenopausal women with low bone mineral density | //population out of scope// |
| Khanizadeh, 2018 | Combination therapy of curcumin and alendronate modulates bone turnover markers and enhances bone mineral density in postmenopausal women with osteoporosis | //non-licenced use of drug// |
| Kilasonia, 2017 | TREATMENT OF OSTEOPOROSIS BY IBANDRONATE IN PATIENTS WITH RHEUMATOID ARTHRITIS | //outcomes out of scope// |
| Kim, 2019 | Comparison of BMD Changes and Bone Formation Marker Levels 3 Years After Bisphosphonate Discontinuation: FLEX and HORIZON-PFT Extension I Trials | //post-hoc cross-RCT comparison // outcomes out of scope// |
| Kobayashi, 2016 | Teriparatide Versus Alendronate for the Preservation of Bone Mineral Density After Total Hip Arthroplasty - A randomized Controlled Trial | //non-licenced use of drug// |
| Koivisto, 2017 | The effect of zoledronic acid on type and volume of Modic changes among patients with low back pain | //population out of scope// |
| Koivisto 2019 | The effect of zoledronic acid on serum biomarkers among patients with chronic low back pain and modic changes in lumbar magnetic resonance imaging | //population and outcomes out of scope// |
| Koivisto, 2014 | Efficacy of zoledronic acid for chronic low back pain associated with Modic changes in magnetic resonance imaging | //population out of scope// |
| Kuroda, 2017 | Acute phase reactions after intravenous infusion of zoledronic acid in Japanese patients with osteoporosis: sub-analysis of the phase III (ZONE) study | //outcomes out of scope//conference abstract// |
| Lee, 2015 | Does Zoledronate Prevent Femoral Head Collapse from Osteonecrosis? A Prospective, Randomized, Open-Label, Multicenter Study | //population out of scope// |
| Lee, 2019 | To withhold or to implement bisphosphonate after cementless hip arthroplasty: a dilemma in elderly hip fracture patients | //study design out of scope// |
| Lim, 2017 | Effect of osteoporosis medications on refracture and mortality following hip fracture surgery in postmenopausal women: a prospective randomized trial | //comparison out of scope// |
| Lin, 2019 | Efficacy of vitamin D plus calcium with/without alendronate on bone metabolism in immunologic thrombocytopenic purpura patients with steroid treatment: nine-month results of a randomized, double-blinded, controlled trial | //non-licenced use of drug//outcomes out of scope// |
| Livi, 2017 | BONADIUV trial: A single blind, randomized placebo controlled phase II study using oral ibandronate for osteopenic women receiving adjuvant aromatase inhibitors: Final safety analysis | //conference abstract//parallel publication// |
| Livi, 2017b | A single-blind, randomized, placebo-controlled phase II study to evaluate the impact of oral ibandronate on bone mineral density in osteopenic breast cancer patients receiving adjuvant aromatase inhibitors: Final results of the single-center BONADIUV trial | //conference abstract// |
| Magaziner, 2014 | Subgroup variations in bone mineral density response to zoledronic acid after hip fracture | //subgroup analysis// |
| Majithia, 2016 | Zoledronic acid for treatment of osteopenia and osteoporosis in women with primary breast cancer undergoing adjuvant aromatase inhibitor therapy: a 5-year follow-up | //non-licenced use of drug// |
| Majumber, 2015 | MANDIBULAR BONE CHANGES IN POSTMENOPAUSAL OSTEOPOROTIC PATIENTS AFTER TREATMENT WITH ZOLEDRONIC ACID | //study design and outcomes of the scope// |
| Malhotra, 2019 | COMPARATIVE STUDY OF WEEKLY ALENDRONATE VS. YEARLY ZOLEDRONIC ACID INJECTION IN TREATMENT OF POSTMENOPAUSAL OSTEOPOROSIS IN TERMS OF EFFICACY, COMPLIANCE AND BONE MARKERS ESTIMATION | //conference abstract – not enough information//outcomes out of scope// |
| Marquez, 2019 | A Randomized Trial of Zoledronic Acid to Prevent Bone Loss in the First Year after Kidney Transplantation | //population out of scope// |
| Meier, 2014 | Effect of ibandronate on spontaneous osteonecrosis of the knee: a randomized, double-blind, placebo-controlled trial | //non-licenced use of drug//population out of the scope// |
| Miguel, 2014 | Effect of Risedronate on Bone Mineral Density and Trabecular Bone Score in Liver Postransplantation patients after one year of follow-up | //conference abstract – not enough information// |
| Miller, 2020 | Efficacy and safety of denosumab vs. bisphosphonates in postmenopausal women previously treated with oral bisphosphonates | //comparison out of scope// |
| Morita, 2020 | Effect of switching administration of alendronate after teriparatide for the prevention of BMD loss around the implant after total hip arthroplasty, 2-year follow-up: a randomized controlled trial | //non-licenced use of drug// |
| Muren, 2015 | No effect of risedronate on femoral periprosthetic bone loss following total hip arthroplasty | //non-licenced use of drug//population out of scope// |
| Nagashima, 2017 | Optimizing a single monthly dose of risedronate based on its weekly dose in healthy Japanese postmenopausal women | //non-licenced use of drug// |
| Nakamura, 2015 | Clinical efficacy and safety of monthly oral ibandronate 100 mg versus monthly intravenous ibandronate 1 mg in Japanese patients with primary osteoporosis | //non-licenced use of drug// |
| Nakamura, 2014 | Clinical Trials Express: fracture risk reduction with denosumab in Japanese postmenopausal women and men with osteoporosis: denosumab fracture intervention randomized placebo controlled trial (DIRECT) | //non-licenced use of drug// |
| Nakano, 2014 | Higher response with bone mineral density (BMD) increase and bone turnover reduction following treatment with monthly injectable ibandronate (IBN) for patients (PTS) with osteoporosis in the mover study | //non-licenced use of drug// |
| Nakano, 2016 | Higher response with bone mineral density increase with monthly injectable ibandronate 1 mg compared with oral risedronate in the MOVER study | //non-licenced use of drug// |
| Natsag, 2016 | Vitamin D, osteoprotegerin/receptor activator of nuclear factor-kappaB ligand (OPG/RANKL) and inflammation with alendronate treatment in HIV-infected patients with reduced bone mineral density | //outcomes out of scope// |
| Naylor, 2016 | Response of bone turnover markers to three oral bisphosphonate therapies in postmenopausal osteoporosis: the TRIO study | //outcomes out of scope// |
| Naylor, 2017 | Offset of effect of oral bisphosphonates on bone in postmenopausal osteoporosis: the Trio study | //comparisons and outcomes out of scope// |
| Naylor, 2019 | Clinical utility of bone turnover markers in monitoring the withdrawal of treatment with oral bisphosphonates in postmenopausal osteoporosis | //conference paper//parallel publication// |
| Niimi, 2018 | Efficacy of Switching From Teriparatide to Bisphosphonate or Denosumab: a Prospective, Randomized, Open-Label Trial | //comparison out of scope// |
| Ofotokun, 2020 | Antiretroviral Therapy-induced Bone Loss is Durably Suppressed by a Single Dose of Zoledronic Acid in Treatment-naÃ¯ve Persons with HIV Infection: a Phase IIB Trial | //population out of scope// |
| Paggiosi, 2014 | A comparison of the effects of three oral bisphosphonates on the peripheral skeleton in postmenopausal osteoporosis: the trio study | //outcomes out of scope// |
| Pastore, 2014 | Sequentialtherapy in severe osteoporosis | //non-licenced use of drug// |
| Patel, 2017 | The Effects of Re-challenge in Patients with a History of Acute Anterior Uveitis Following Intravenous Zoledronate | //secondary analysis//outcomes out of scope// |
| Prasad, 2016 | Risedronate may preserve bone microarchitecture in breast cancer survivors on aromatase inhibitors: a randomized, controlled clinical trial | //wrong population//outcomes out of scope// |
| Prieto-Alhambra, 2014 | Fracture prevention in patients with cognitive impairment presenting with a hip fracture: secondary analysis of data from the HORIZON Recurrent Fracture Trial | //secondary analysis//outcomes out of scope// |
| Raj, 2019 | Effect of zoledronate in hepatic osteodystrophy: a double-blinded placebo-controlled trial | //conference paper – not enough info// |
| Ralston, 2017 | Effect of alendronic acid on fracture healing-a randomised clinical trial | //conference paper//outcomes out of scope// |
| Reid, 2018 | Fracture Prevention in Osteopenic Postmenopausal Women with Zoledronic Acid Every 18 Months, a Randomized Controlled Trial | //conference paper//parallel publication// |
| Reid, 2018 | Zoledronate every 18 months for 6 years in osteopenic postmenopausal women reduces non-vertebral fractures and height loss | //conference paper//parallel publication// |
| Reid, 2018 | Zoledronate every 18 months for 6 years in osteopenic postmenopausal women: effects on fractures and non-skeletal endpoints | //conference paper//parallel publication// |
| Reid, 2020 | Zoledronate Slows Weight Loss and Maintains Fat Mass in Osteopenic Older Women: secondary Analysis of a Randomized Controlled Trial | //outcomes out of scope// |
| Reid, 2021 | Predictors of Fracture in Older Women With Osteopenic Hip Bone Mineral Density Treated With Zoledronate | //outcomes out of scope// |
| Reid, 2019 | Anti-fracture efficacy of zoledronate in subgroups of osteopenic postmenopausal women: secondary analysis of a randomized controlled trial | //subgroup analysis//outcomes out of scope// |
| Roh, 2018 | Comparative adherence to weekly oral and quarterly intravenous bisphosphonates among patients with limited heath literacy who sustained distal radius fractures | //outcome out of scope// |
| Rooney, 2019 | The prevention and treatment of glucocorticoid-induced osteopaenia in juvenile rheumatic disease: a randomised double-blind controlled trial | //population out of scope// |
| Rooney, 2017 | Prevention and treatment of steroid induced osteopaenia in children and adolescents with rheumatic diseases: the pops study | //non-licenced use of drug// |
| Roux, 2014 | Vitamin D status and bone mineral density changes during alendronate treatment in postmenopausal osteoporosis | //comparison out of scope// |
| Sánchez-Escuredo, 2017 | Monthly ibandronate versus weekly risedronate treatment for low bone mineral density in stable renal transplant patients | //population out of scope// |
| Safer, 2016 | Effects of Bisphosphonates and Calcium plus Vitamin-D Supplements on Cognitive Function in Postmenopausal Osteoporosis | //conference paper-not enough information// |
| Saito, 2015 | Open-label randomized parallel controlled study comparing bone mineral density between alendronate plus alfacalcidol combination and single administration of alfacalcidol in postmenopausal women receiving aromatase inhibitor as adjuvant therapy | //conference paper – not enough information// |
| Scott, 2014 | A single-blind, randomized, placebo-controlled phase II study to evaluate the impact of oral bisphosphonate treatment on bone mineral density in osteopenic women receiving adjuvant aromatase inhibitors: interim analysis of "BONADIUV" trial | //interim analysis – parallel publication// |
| Seefried, 2017 | Prospective, randomized, double-blind, placebo-controlled trial to evaluate efficacy and safety of zoledronic acid for the treatment of bone marrow lesions-ZoMARS | //non-licenced use of drug// |
| Sestak, 2014 | Risedronate Prevents Anastrozole-Induced Bone Loss In The IBIS-II Prevention Trial | //conference paper// |
| Smith, 2014 | Randomized controlled trial of early zoledronic acid in men with castration-sensitive prostate cancer and bone metastases: results of CALGB 90202 (alliance) | //population out of scope// |
| Swafford, 2020 | Risedronate to Prevent Bone Loss After Sleeve Gastrectomy: Study Design and Feasibility Report of a Pilot Randomized Controlled Trial | //non-licenced use of drug// |
| Solling, 2020 | Treatment with zoledronic acid subsequent to treatment with denosumab | //comparison out of scope// |
| Songpatanasilp, 2018 | Open-label study of treatment with alendronate sodium plus vitamin D in men and women with osteoporosis in Thailand | //study design out of scope// |
| Tan, 2015 | FES-rowing training improves bone strength of the paralyzed legs in a dose-dependent fashion | //comparison out of scope// |
| Tanaka, 2016 | Analysis of bone metabolism during early stage and clinical benefits of early intervention with alendronate in patients with systemic rheumatic diseases treated with high-dose glucocorticoid: Early DIagnosis and Treatment of OsteopoRosis in Japan (EDITOR-J) study | //non-licenced use of drug// |
| Tanner, 2019 | Prevention of fractures in Parkinson's disease (PD) with a single infusion of zoledronic acid (ZA) | //population out of scope// |
| Tariq, 2015 | Effect of ibandronate therapy on serum homocysteine and leptin in postmenopausal osteoporotic females | //study design out of scope// |
| Tantiworawit, 2019 | Efficacy of alendronate in treatment of thalassemia-associated osteoporosis: a randomized controlled trial | //conference abstract - outcomes out of scope// |
| Thomas, 2014 | CHANGES IN LUMBAR SPINE QCT, DXA AND TBS FOLLOWING TREATMENT WITH DENOSUMAB (DINIAB), ALENDRONATE (ALN), OR PLACEBO (PBO) IN POSTMENOPAUSAL WOMEN WITH LOW BONE MASS | //outcomes out of scope//lead author unable to provide us with the full-text// |
| Vittinghoff, 2015 | Bisphosphonates reduce fracture risk in postmenopausal women with diabetes: results from FIT and HORIZON trials | //post-hoc 2ary analysis//outcomes out of scope// |
| Zhang, 2014 | Response in Subgroups based on Baseline 25-Hydroxyvitamin D and Bone Turnover Markers: Alendronate Sodium/Vitamin D-3 versus Calcitriol for Treatment of Osteoporosis in Chinese Women | //conference paper – not enough information// |
| Zhang, 2015 | Characteristics associated with bone mineral density increase by 1-year ALN/D5600 treatment in a randomized, controlled study on postmenopausal osteoporosis in Chinese women | //parallel publication//conference paper – not enough information// |
| Zhang, 2015 | A revisit to safety evaluation of calcium/phosphate metabolism in a 12-month, randomized, controlled study on alendronate/vitamin D3 versus calcitriol in Chinese osteoporotic women | //conference paper//outcomes out of scope// |
| Zebaze, 2014 | Differing effects of denosumab and alendronate on cortical and trabecular bone | //outcomes out of scope// |
| Yang, 2018 | Early initiation of zoledronic acid does not impact bone healing or clinical outcomes of hallux valgus orthomorphia | //non-licenced use of drug// |
| Yi, 2020 | Effects of percutaneous kyphoplasty combined with zoledronic acid injection on osteoporotic vertebral compression fracture and bone metabolism indices | //comparison out of scope// |
| van Spil, 2019 | The zodiak trial: protocol of a double-blind, placebo-controled, two-year clinical trial of zoledronic acid as a disease-modifying drug in knee osteoarthritis with bone marrow lesions | //non-licenced use of drug//outcomes out of scope// |
| Zhou, 2019 | Effects of zoledronic acid on bone mineral density around prostheses and bone metabolism markers after primary total hip arthroplasty in females with postmenopausal osteoporosis | //outcomes out of scope// |
| Cai, 2018 | Effect of Zoledronic Acid and Denosumab in Patients With Low Back Pain and Modic Change: A Proof-of-Principle Trial | //population out of scope//non-licenced use of drug// |
| Cai, 2020 | Effect of Intravenous Zoledronic Acid on Tibiofemoral Cartilage Volume Among Patients With Knee Osteoarthritis With Bone MarrowLesions A Randomized Clinical Trial | //non-licenced dose of drug// |
| Cai, 2019 | Zoledronic acid plus methylprednisolone versus zoledronic acid or placebo in symptomatic knee osteoarthritis: a randomized controlled trial | //population out of scope// |
| Liu, 2019a | Clinical effect observation of intravenous application of zoledronic acid in patients with cervical spondylosis and osteoporosis after anterior cervical discectomy and fusion: A randomized controlled study | //population out of scope//study design cannot be attested – no response from authors// |
| Duckworth, 2019 | Effect of Alendronic Acid on Fracture Healing: A Multicenter Randomized Placebo-Controlled Trial | //outcomes out of scope// |
| Schnitzer, 2016 | Zoledronic Acid Treatment After Acute Spinal Cord Injury: Results of a Randomized, Placebo-Controlled Pilot Trial | //non-licenced use of drug// |
| Flodin, 2014 | Additive effects of nutritional supplementation, together with bisphosphonates, on bone mineral density after hip fracture: a 12-month randomized controlled study | //outcomes out of scope// |
| Stuss, 2016 | Assessment of OPG, RANKL, bone turnover markers serum levels, and BMD after treatment with strontium ranelate and ibandronate in patients with postmenopausal osteoporosis | //outcomes out of scope – not enough information on BMD data// |
| Bell, 2016 | Potential Usefulness of BMD and Bone Turnover Monitoring of Zoledronic Acid Therapy Among Women With Osteoporosis: Secondary Analysis of Randomized Controlled Trial Data | //parallel publication//outcomes out of scope// |
| Tan, 2019 | Alendronate/Vitamin D for attenuating bone mineral density loss during antiretroviral initiation: a pilot randomized controlled trial | //outcomes out of scope// |
| Muschitz, 2014 | Overlapping and Continued Alendronate or Raloxifene Administration in Patients on Teriparatide: Effects on Areal and Volumetric Bone Mineral Density—The CONFORS Study | //study design out of scope// |
| McClung,2020 | A single dose of zoledronate preserves bone mineral density for up to 2 years after a second course of romosozumab | //study design out of scope// |
| Sestak, 2021 | Off-treatment bone mineral density changes in postmenopausal women receiving anastrozole for 5 years: 7-year results from the IBIS-II prevention trial | //outcomes out of scope// |
| Sestak, 2019 | Comparison of risedronate versus placebo in preventing anastrozole-induced bone loss in women at high risk of developing breast cancer with osteopenia | //comparison out of scope// |
| Negredo, 2015 | Comparison of two different strategies of treatment with zoledronate in HIV-infected patients with low bone mineral density: single dose versus two doses in 2 years | //data requested from the authors were not provided// |
| van Bodegraven, 2014 | Treatment of bone loss in osteopenic patients with Crohn’s disease: a double-blind, randomised trial of oral risedronate 35 mg once weekly or placebo, concomitant with calcium and vitamin D supplementation | //data requested from the authors were not provided// |
| Meattinni, 2019 | Abstract P4-16-04: Oral ibandronate for osteopenic breast cancer patients receiving adjuvant aromatase inhibitors: secondary 5-year survival outcomes analysis of the single-center phase 2 BONADIUV trial | //parallel publication of Livi’s 2019// |
| Ha, 2020 | Effect of bisphosphonate on the prevention of bone loss in patients with gastric cancer after gastrectomy: A randomized controlled trial | //population out of scope// |
| Borggard, 2020 | Alendronate suppresses initiation of bone formation in cortical bone in postmenopausal osteoporosis | //outcomes out of scope// |
| Greene, 2020 | Effects of Risedronate on Trabecular and Cortical Volumetric Bone Density of the Hip after Sleeve Gastrectomy: a Pilot Randomized Controlled Trial | //non licenced use of drug// conference poster// |
| Haider, 2020 | Effects of Zoledronic Acid and Ambulation on Hip Bone Mineral Density after Acute Spinal Cord Injury: Year 1 of a Randomized Controlled Trial | //population's eligibility cannot be defined - poster// |
| Marques, 2019 | A Randomized Trial of Zoledronic Acid to Prevent Bone Loss in the First Year after Kidney Transplantation | //population out of scope// |
| Oleson, 2020 | The effect of zoledronic acid on attenuation of bone loss at the hip and knee following acute traumatic spinal cord injury: a randomized-controlled study | //population out of scope// |
| Morita, 2020 | Effect of switching administration of alendronate after teriparatide for the prevention of BMD loss around the implant after total hip arthroplasty, 2-year followup: a randomized controlled trial | //outcomes out of scope// |
| Cummings, 2020 | Probability of Achieving T-scores Goals Above -2.5 With Alendronate or Romosozumab Followed by Alendronate or Denosumab | //comparison out of scope// |
| Zekavat, 2019 | Comparative effectiveness of alendronate and zoledronic acid on bone mass improvement in transfusion‑dependent thalassemia patients | //population out of scope//study design out of scope// |
| Grey, 2020 | Ten Years of Very Infrequent Zoledronate Therapy in Older Women: An Open-Label Extension of a Randomized Trial | //comparison out of scope// |
| Lu, 2020 | Efficacy of annual zoledronic acid in initial percutaneous kyphoplasty patients with osteoporotic vertebral compression fractures: a 3-year follow-up study | //population out of scope// |
| Zhang, 2020 | The effect of zoledronic acid combined with PKP in the treatment of osteoporotic vertebral body compression fractures | //population out of scope//study design out of scope// |
| Liao, 2018 | Clinical characteristics associated with bone mineral density improvement after 1-year alendronate/vitamin d3 or calcitriol treatment Exploratory results from a phase 3, randomized, controlled trial on postmenopausal osteoporotic women in China | //post-hoc analysis//outcomes out of scope// |

**Appendix 11. References of the studies drawn from the original review**

**References**

Adami S, Passeri M, Ortolani S, Broggini M, Carratelli L, Caruso I, Gandolini G, Gnessi L, Laurenzi M, Lombardi A, Norbiato G. Effects of oral alendronate and intranasal salmon calcitonin on bone mass and biochemical markers of bone turnover in postmenopausal women with osteoporosis. Bone. 1995 Oct 1;17(4):383-90.

Black DM, Delmas PD, Eastell R, Reid IR, Boonen S, Cauley JA, Cosman F, Lakatos P, Leung PC, Man Z, Mautalen C. Once-yearly zoledronic acid for treatment of postmenopausal osteoporosis. New England Journal of Medicine. 2007 May 3;356(18):1809-22.

Black DM, Thompson DE, Bauer DC, Ensrud K, Musliner T, Hochberg MC, Nevitt MC, Suryawanshi S, Cummings SR, FIT Research Group. Fracture risk reduction with alendronate in women with osteoporosis: the Fracture Intervention Trial. The Journal of Clinical Endocrinology & Metabolism. 2000 Nov 1;85(11):4118-24.

Black DM, Cummings SR, Karpf DB, Cauley JA, Thompson DE, Nevitt MC, Bauer DC, Genant HK, Haskell WL, Marcus R, Ott SM. Randomised trial of effect of alendronate on risk of fracture in women with existing vertebral fractures. The Lancet. 1996 Dec 7;348(9041):1535-41.

Bone HG, Greenspan SL, McKeever C, Bell N, Davidson M, Downs RW, Emkey R, Meunier PJ, Miller SS, Mulloy AL, Recker RR. Alendronate and estrogen effects in postmenopausal women with low bone mineral density. The Journal of Clinical Endocrinology & Metabolism. 2000 Feb 1;85(2):720-6.

Boonen S, Orwoll ES, Wenderoth D, Stoner KJ, Eusebio R, Delmas PD. Once‐weekly risedronate in men with osteoporosis: results of a 2‐year, placebo‐controlled, double‐blind, multicenter study. Journal of bone and mineral research. 2009 Apr;24(4):719-25.

Boonen S, Reginster JY, Kaufman JM, Lippuner K, Zanchetta J, Langdahl B, Rizzoli R, Lipschitz S, Dimai HP, Witvrouw R, Eriksen E. Fracture risk and zoledronic acid therapy in men with osteoporosis. New England Journal of Medicine. 2012 Nov 1;367(18):1714-23.

Carfora E, Sergio F, Bellini P, Sergio C, Falco D, Zarcone R. Effect of treatment of postmenopausal osteoporosis with continuous daily oral alendronate and the incidence of fractures. Gazzetta Medica Italiana Archivio per le Scienze Mediche. 1998 Aug;157:105-10.

Choo C, Lukka H, Kiss A, Danjoux C. Double-blinded, Placebo-controlled Randomized Study Evaluating the Efficacy of Risedronate to Prevent the Loss of Bone Mineral Density in Non-metastatic Prostate Cancer Patients Undergoing Radiotherapy Plus 2-3 Years of Androgen Ablation Therapy. International Journal of Radiation Oncology• Biology• Physics. 2011 Oct 1;81(2):S42.

Cohen S, Levy RM, Keller M, Boling E, Emkey RD, Greenwald M, Zizic TM, Wallach S, Sewell KL, Lukert BP, Axelrod DW. Risedronate therapy prevents corticosteroid‐induced bone loss: a twelve‐month, multicenter, randomized, double‐blind, placebo‐controlled, parallel‐group study. Arthritis & Rheumatism: Official Journal of the American College of Rheumatology. 1999 Nov;42(11):2309-18.

Cummings SR, Black DM, Thompson DE, Applegate WB, Barrett-Connor E, Musliner TA, Palermo L, Prineas R, Rubin SM, Scott JC, Vogt T. Effect of alendronate on risk of fracture in women with low bone density but without vertebral fractures: results from the Fracture Intervention Trial. JAMA. 1998 Dec 23;280(24):2077-82.

Dursun N, Dursun E, Yalcin S. Comparison of alendronate, calcitonin and calcium treatments in postmenopausal osteoporosis. International journal of clinical practice. 2001 Oct 1;55(8):505-9.

Fogelman I, Ribot C, Smith R, Ethgen D, Sod E, Reginster for the bmd-mn Study Group JY. Risedronate reverses bone loss in postmenopausal women with low bone mass: results from a multinational, double-blind, placebo-controlled trial. The Journal of Clinical Endocrinology & Metabolism. 2000 May 1;85(5):1895-900.

Greenspan SL, Resnick NM, Parker RA. Combination therapy with hormone replacement and alendronate for prevention of bone loss in elderly women: a randomized controlled trial. JAMA. 2003 May 21;289(19):2525-33.

Greenspan SL, Schneider DL, McClung MR, Miller PD, Schnitzer TJ, Bonin R, Smith ME, DeLucca P, Gormley GJ, Melton ME. Alendronate improves bone mineral density in elderly women with osteoporosis residing in long-term care facilities: a randomized, double-blind, placebo-controlled trial. Annals of internal medicine. 2002 May 21;136(10):742-6.

Harris ST, Watts NB, Genant HK, McKeever CD, Hangartner T, Keller M, Chesnut III CH, Brown J, Eriksen EF, Hoseyni MS, Axelrod DW. Effects of risedronate treatment on vertebral and nonvertebral fractures in women with postmenopausal osteoporosis: a randomized controlled trial. JAMA. 1999 Oct 13;282(14):1344-52.

Hooper MJ, Ebeling PR, Roberts AP, Graham JJ, Nicholson GC, D'Emden M, Ernst TF, Wenderoth D. Risedronate prevents bone loss in early postmenopausal women: a prospective randomized, placebo-controlled trial. Climacteric. 2005 Sep 1;8(3):251-62.

Klotz LH, McNeill IY, Kebabdjian M, Zhang L, Chin JL, Canadian Urology Research Consortium. A phase 3, double-blind, randomised, parallel-group, placebo-controlled study of oral weekly alendronate for the prevention of androgen deprivation bone loss in nonmetastatic prostate cancer: the Cancer and Osteoporosis Research with Alendronate and Leuprolide (CORAL) study. European urology. 2013 May 1;63(5):927-35.

Lester JE, Dodwell D, Purohit OP, Gutcher SA, Ellis SP, Thorpe R, Horsman JM, Brown JE, Hannon RA, Coleman RE. Prevention of anastrozole-induced bone loss with monthly oral ibandronate during adjuvant aromatase inhibitor therapy for breast cancer. Clinical Cancer Research. 2008 Oct 1;14(19):6336-42.

Leung JY, Ho AY, Ip TP, Lee G, Kung AW. The efficacy and tolerability of risedronate on bone mineral density and bone turnover markers in osteoporotic Chinese women: a randomized placebo-controlled study. Bone. 2005 Feb 1;36(2):358-64.

Liberman UA, Weiss SR, Bröll J, Minne HW, Quan H, Bell NH, Rodriguez-Portales J, Downs Jr RW, Dequeker J, Favus M, Seeman E. Effect of oral alendronate on bone mineral density and the incidence of fractures in postmenopausal osteoporosis. New England Journal of Medicine. 1995 Nov 30;333(22):1437-44.

Lyles KW, Colón-Emeric CS, Magaziner JS, Adachi JD, Pieper CF, Mautalen C, Hyldstrup L, Recknor C, Nordsletten L, Moore KA, Lavecchia C. Zoledronic acid and clinical fractures and mortality after hip fracture. New England Journal of Medicine. 2007 Nov 1;357(18):1799-809.

McClung MR, Bolognese MA, Sedarati F, Recker RR, Miller PD. Efficacy and safety of monthly oral ibandronate in the prevention of postmenopausal bone loss. Bone. 2009 Mar 1;44(3):418-22.

McClung MR, Geusens P, Miller PD, Zippel H, Bensen WG, Roux C, Adami S, Fogelman I, Diamond T, Eastell R, Meunier PJ. Effect of risedronate on the risk of hip fracture in elderly women. New England journal of medicine. 2001 Feb 1;344(5):333-40.

McClung M, Miller P, Recknor C, Mesenbrink P, Bucci-Rechtweg C, Benhamou CL. Zoledronic acid for the prevention of bone loss in postmenopausal women with low bone mass: a randomized controlled trial. Obstetrics & Gynecology. 2009 Nov 1;114(5):999-1007.

Miller PD, Epstein S, Sedarati F, Reginster JY. Once-monthly oral ibandronate compared with weekly oral alendronate in postmenopausal osteoporosis: results from the head-to-head MOTION study. Current medical research and opinion. 2008 Jan 1;24(1):207-13.

Muscoso E, Puglisi N, Mamazza C, Giudice FL, Testai M, Abbate S, Santangelo A, Panebianco P, Maugeri D. Antiresorption therapy and reduction in fracture susceptibility in the osteoporotic elderly patient: open study. EUROPEAN REVIEW FOR MEDICAL AND PHARMACOLOGICAL SCIENCES. 2004 Mar 1;8:97-102.

Orwoll E, Ettinger M, Weiss S, Miller P, Kendler D, Graham J, Adami S, Weber K, Lorenc R, Pietschmann P, Vandormael K. Alendronate for the treatment of osteoporosis in men. New England Journal of Medicine. 2000 Aug 31;343(9):604-10.

Pols HA, Felsenberg D, Hanley DA, Štepán J, Munoz-Torres M, Wilkin TJ, Qin-Sheng G, Galich AM, Vandormael K, Yates AJ, Stych B. Multinational, placebo-controlled, randomized trial of the effects of alendronate on bone density and fracture risk in postmenopausal women with low bone mass: results of the FOSIT study. Osteoporosis International. 1999 Apr;9(5):461-8.

Reginster JY, Minne HW, Sorensen OH, Hooper M, Roux C, Brandi ML, Lund B, Ethgen D, Pack S, Roumagnac I, Eastell R. Randomized trial of the effects of risedronate on vertebral fractures in women with established postmenopausal osteoporosis. Osteoporosis international. 2000 Jan;11(1):83-91.

Reid DM, Devogelaer JP, Saag K, Roux C, Lau CS, Reginster JY, Papanastasiou P, Ferreira A, Hartl F, Fashola T, Mesenbrink P. Zoledronic acid and risedronate in the prevention and treatment of glucocorticoid-induced osteoporosis (HORIZON): a multicentre, double-blind, double-dummy, randomised controlled trial. The Lancet. 2009 Apr 11;373(9671):1253-63.

Reid DM, Hosking D, Kendler D, Brandi ML, Wark JD, Weryha G, Marques-Neto JF, Gaines KA, Verbruggen N, Melton ME. Alendronic Acid Produces Greater Effects than Risedronic Acid on Bone Density and Turnover in Postmenopausal Women with Osteoporosis. Clinical drug investigation. 2006 Feb;26(2):63-74.

Reid DM, Hughes RA, Laan RF, Sacco‐Gibson NA, Wenderoth DH, Adami S, Eusebio RA, Devogelaer JP. Efficacy and safety of daily risedronate in the treatment of corticosteroid‐induced osteoporosis in men and women: a randomized trial. Journal of Bone and Mineral Research. 2000 Jun;15(6):1006-13.

Ringe JD, Faber H, Farahmand P, Dorst A. Efficacy of risedronate in men with primary and secondary osteoporosis: results of a 1-year study. Rheumatology international. 2006 Mar 1;26(5):427-31.

Rosen CJ, Hochberg MC, Bonnick SL, McClung M, Miller P, Broy S, Kagan R, Chen E, Petruschke RA, Thompson DE, de Papp AE. Treatment with once‐weekly alendronate 70 mg compared with once‐weekly risedronate 35 mg in women with postmenopausal osteoporosis: a randomized double‐blind study. Journal of bone and mineral research. 2005 Jan;20(1):141-51.

Saag KG, Emkey R, Schnitzer TJ, Brown JP, Hawkins F, Goemaere S, Thamsborg G, Liberman UA, Delmas PD, Malice MP, Czachur M. Alendronate for the prevention and treatment of glucocorticoid-induced osteoporosis. New England Journal of Medicine. 1998 Jul 30;339(5):292-9.

Sarioglu M, Tuzun C, Unlu Z, Tikiz C, Taneli F, Uyanik BS. Comparison of the effects of alendronate and risedronate on bone mineral density and bone turnover markers in postmenopausal osteoporosis. Rheumatology international. 2006 Jan 1;26(3):195-200.

Taxel P, Dowsett R, Richter L, Fall P, Klepinger A, Albertsen P. Risedronate prevents early bone loss and increased bone turnover in the first 6 months of luteinizing hormone‐releasing hormone‐agonist therapy for prostate cancer. BJU international. 2010 Nov;106(10):1473-6.

**Appendix 12. PRISMA NMA Checklist of Items to Include When Reporting A Systematic Review Involving a Network Meta-analysis**

| **Section/Topic** | **Item #** | **Checklist Item** | **Reported on Page #** |
| --- | --- | --- | --- |
| **TITLE** |  |  |  |
| Title | 1 | Identify the report as a systematic review *incorporating a network meta-analysis (or related form of meta-analysis).* | 1 |
|  |  |  |  |
| **ABSTRACT** |  |  |  |
| Structured summary | 2 | Provide a structured summary including, as applicable:  **Background:** main objectives  **Methods:** data sources; study eligibility criteria, participants, and interventions; study appraisal; and *synthesis methods, such as network meta-analysis.*  **Results:** number of studies and participants identified; summary estimates with corresponding confidence/credible intervals; *treatment rankings may also be discussed. Authors may choose to summarize pairwise comparisons against a chosen treatment included in their analyses for brevity.*  **Discussion/Conclusions:** limitations; conclusions and implications of findings.  **Other:** primary source of funding; systematic review registration number with registry name. | 2 (plus pages 22-23 for ‘Other’) |
|  |  |  |  |
| **INTRODUCTION** |  |  |  |
| Rationale | 3 | Describe the rationale for the review in the context of what is already known*, including mention of why a network meta-analysis has been conducted.* | 4 |
| Objectives | 4 | Provide an explicit statement of questions being addressed, with reference to participants, interventions, comparisons, outcomes, and study design (PICOS). | 4 |
|  |  |  |  |
| **METHODS** |  |  |  |
| Protocol and registration | 5 | Indicate whether a review protocol exists and if and where it can be accessed (e.g., Web address); and, if available, provide registration information, including registration number. | 2, 5 |
| Eligibility criteria | 6 | Specify study characteristics (e.g., PICOS, length of follow-up) and report characteristics (e.g., years considered, language, publication status) used as criteria for eligibility, giving rationale. *Clearly describe eligible treatments included in the treatment network, and note whether any have been clustered or merged into the same node (with justification).* | 5 |
| Information sources | 7 | Describe all information sources (e.g., databases with dates of coverage, contact with study authors to identify additional studies) in the search and date last searched. | 5,6 |
| Search | 8 | Present full electronic search strategy for at least one database, including any limits used, such that it could be repeated. | Appendix 1 |
| Study selection | 9 | State the process for selecting studies (i.e., screening, eligibility, included in systematic review, and, if applicable, included in the meta-analysis). | 7 |
| Data collection process | 10 | Describe method of data extraction from reports (e.g., piloted forms, independently, in duplicate) and any processes for obtaining and confirming data from investigators. | 7 |
| Data items | 11 | List and define all variables for which data were sought (e.g., PICOS, funding sources) and any assumptions and simplifications made. | 7 |
| **Geometry of the network** | **S1** | Describe methods used to explore the geometry of the treatment network under study and potential biases related to it. This should include how the evidence base has been graphically summarized for presentation, and what characteristics were compiled and used to describe the evidence base to readers. | 7-8 |
| Risk of bias within individual studies | 12 | Describe methods used for assessing risk of bias of individual studies (including specification of whether this was done at the study or outcome level), and how this information is to be used in any data synthesis. | 8 |
| Summary measures | 13 | State the principal summary measures (e.g., risk ratio, difference in means). *Also describe the use of additional summary measures assessed, such as treatment rankings and surface under the cumulative ranking curve (SUCRA) values, as well as modified approaches used to present summary findings from meta-analyses.* | 8-10 |
| Planned methods of analysis | 14 | Describe the methods of handling data and combining results of studies for each network meta-analysis. This should include, but not be limited to:   - *Handling of multi-arm trials;* - *Selection of variance structure;* - *Selection of prior distributions in Bayesian analyses; and* - *Assessment of model fit.* | 8-10 |
| **Assessment of Inconsistency** | **S2** | Describe the statistical methods used to evaluate the agreement of direct and indirect evidence in the treatment network(s) studied. Describe efforts taken to address its presence when found. | 10-11 |
| Risk of bias across studies | 15 | Specify any assessment of risk of bias that may affect the cumulative evidence (e.g., publication bias, selective reporting within studies). | 11 |
| Additional analyses | 16 | Describe methods of additional analyses if done, indicating which were pre-specified. This may include, but not be limited to, the following:   - Sensitivity or subgroup analyses; - Meta-regression analyses; - *Alternative formulations of the treatment network; and* - *Use of alternative prior distributions for Bayesian analyses (if applicable).* | 12 |
|  |  |  |  |
| **RESULTS†** |  |  |  |
| Study selection | 17 | Give numbers of studies screened, assessed for eligibility, and included in the review, with reasons for exclusions at each stage, ideally with a flow diagram. | 13 (plus Appendix 10) |
| **Presentation of network structure** | **S3** | Provide a network graph of the included studies to enable visualization of the geometry of the treatment network. | Appendix 3 |
| **Summary of network geometry** | **S4** | Provide a brief overview of characteristics of the treatment network. This may include commentary on the abundance of trials and randomized patients for the different interventions and pairwise comparisons in the network, gaps of evidence in the treatment network, and potential biases reflected by the network structure. | 13-14 |
| Study characteristics | 18 | For each study, present characteristics for which data were extracted (e.g., study size, PICOS, follow-up period) and provide the citations. | 14-15 (plus Appendix 2) |
| Risk of bias within studies | 19 | Present data on risk of bias of each study and, if available, any outcome level assessment. | 15 (plus Appendix 6) |
| Results of individual studies | 20 | For all outcomes considered (benefits or harms), present, for each study: 1) simple summary data for each intervention group, and 2) effect estimates and confidence intervals. *Modified approaches may be needed to deal with information from larger networks.* | Appendix 2 |
| Synthesis of results | 21 | Present results of each meta-analysis done, including confidence/credible intervals. *In larger networks, authors may focus on comparisons versus a particular comparator (e.g. placebo or standard care), with full findings presented in an appendix. League tables and forest plots may be considered to summarize pairwise comparisons.* If additional summary measures were explored (such as treatment rankings), these should also be presented. | 15-18 (plus Appendix 4 & 5) |
| **Exploration for inconsistency** | **S5** | Describe results from investigations of inconsistency. This may include such information as measures of model fit to compare consistency and inconsistency models, *P* values from statistical tests, or summary of inconsistency estimates from different parts of the treatment network. | 15-18 (plus Appendix 8) |
| Risk of bias across studies | 22 | Present results of any assessment of risk of bias across studies for the evidence base being studied. | 18-19 (plus Appendix 7) |
| Results of additional analyses | 23 | Give results of additional analyses, if done (e.g., sensitivity or subgroup analyses, meta-regression analyses*, alternative network geometries studied, alternative choice of prior distributions for Bayesian analyses,* and so forth). | 19-20 (plus Appendix 5) |
|  |  |  |  |
| **DISCUSSION** |  |  |  |
| Summary of evidence | 24 | Summarize the main findings, including the strength of evidence for each main outcome; consider their relevance to key groups (e.g., healthcare providers, users, and policy-makers). | 20-21 |
| Limitations | 25 | Discuss limitations at study and outcome level (e.g., risk of bias), and at review level (e.g., incomplete retrieval of identified research, reporting bias). *Comment on the validity of the assumptions, such as transitivity and consistency. Comment on any concerns regarding network geometry (e.g., avoidance of certain comparisons).* | 22 |
| Conclusions | 26 | Provide a general interpretation of the results in the context of other evidence, and implications for future research. | 22 |
|  |  |  |  |
| **FUNDING** |  |  |  |
| Funding | 27 | Describe sources of funding for the systematic review and other support (e.g., supply of data); role of funders for the systematic review. This should also include information regarding whether funding has been received from manufacturers of treatments in the network and/or whether some of the authors are content experts with professional conflicts of interest that could affect use of treatments in the network. | 22 |
